# Automated identification of *Salmonella* serotype using MALDI-TOF mass spectrometry and machine learning techniques

**DOI:** 10.1128/jcm.00037-25

**Published:** 2025-06-11

**Authors:** Jun Ren, Jintao Xia, Mengyu Zhang, Chunhong Liu, Yuanyuan Xu, Jianing Wu, Yingzhu Li, Mingming Zhou, Shengjie Li, Wenjun Cao

**Affiliations:** 1 Department of Clinical Laboratory, Eye & ENT Hospital, Fudan University 12478, Shanghai, China; 2 Department of Clinical Laboratory, Children’s Hospital, Zhejiang University School of Medicine, National Clinical Research Center for Child Health 26441 , Hangzhou, Zhejiang, China; 3 Department of Clinical Laboratory, Wanbei Coal Electric Group General Hospital 645365 , Suzhou, Anhui Province, China; Endeavor Health, Evanston, Illinois, USA

**Keywords:** *Salmonella *serotype, machine learning, MALDI-TOF MS, SHAP, Streamlit

## Abstract

*Salmonella* serotyping is essential for epidemiological studies and clinical treatment guidance. However, traditional serological agglutination methods are time-consuming, technically complex, and difficult to adopt at scale. Matrix-assisted laser desorption ionization-time of flight mass spectrometry (MALDI-TOF MS) is a rapid and cost-effective microbial identification technique, but it cannot be used to differentiate *Salmonella* serotypes. This study aims to integrate MALDI-TOF MS with machine learning algorithms to develop and validate a model for *Salmonella* serotype identification, improving efficiency and simplifying workflows. A total of 692 *Salmonella* isolates from Children’s Hospital, Zhejiang University School of Medicine (ZUCH) and Wanbei Coal-Electricity Group General Hospital (WCGH) were analyzed using MALDI-TOF MS, generating 2,048 spectra. The ZUCH data were randomly divided into training and internal validation sets. The WCGH data were used as an external validation set. Ten machine learning algorithms were evaluated for their ability to identify eight *Salmonella* serotypes (B, C1, C2/3, D, E, Not A-F, *Salmonella* Typhimurium, and *Salmonella* Enteritidis). From 192 initial features, 16 features were selected for the final model construction. XGBoost demonstrated the best discriminative ability (area under the receiver operating characteristic curve [AUC] = 0.9898, sensitivity = 0.88, and specificity = 0.98) for the training set. The streamlined XGBoost model achieved AUCs of 0.9662 and 0.9778 for the internal and external validation sets, respectively, accurately identifying *Salmonella* serotypes. To enhance usability, the model was deployed as a Streamlit-based application, facilitating interaction and broader application. MALDI-TOF MS combined with XGBoost provides a fast and accurate method for *Salmonella* serotype identification, offering an efficient solution for laboratory diagnostics and epidemiological studies.

**IMPORTANCE:**

*Salmonella* serotyping is vital for outbreak tracking and clinical guidance, but traditional methods are slow and laborious. This study combines matrix-assisted laser desorption ionization-time of flight mass spectrometry with machine learning (XGBoost) to enable rapid, accurate, and cost-effective serotyping. The streamlined model performed excellently in validation and was deployed as a user-friendly Streamlit app, enhancing usability. This innovation simplifies workflows, reduces diagnostic time, and supports scalable use in clinical and public health settings, improving outbreak response and epidemiological research.

## INTRODUCTION

*Salmonella* is a major pathogen responsible for a wide range of infections in both humans and animals, posing substantial threats to public health and economic stability worldwide (1, 2). *Salmonella enterica* is a leading cause of community-acquired bloodstream infections in many low- and middle-income countries (3, 4). The International Vaccine Institute estimated that, in 2010, there were 11.9 million cases of typhoid fever and approximately 129,000 related deaths in low- and middle-income countries (5). Effective treatment, prevention, and control of *Salmonella* outbreak infections rely on rapid and accurate identification of pathogenic agents (6).

Bacterial subtyping within the same genus and species is a critical tool for disease surveillance, outbreak investigations, and epidemiological research (7–9). The primary methods for *Salmonella* subtyping include serotyping, molecular biology techniques, and genomic sequencing, with serotyping serving as the principal screening approach in laboratories (10–13). According to the White–Kauffmann–Le Minor scheme, *Salmonella* serotyping involves the biochemical identification of somatic O antigens, flagellar H antigens, and capsular Vi antigens in combination (14, 15). There are more than 150 commonly used diagnostic sera for *Salmonella*, making the identification process both time-consuming and labor-intensive (16). Furthermore, conducting the tests and interpreting the results require trained specialists. Given the limitations of serological testing, there is a pressing need for an alternative *Salmonella* typing method that is simpler, faster, and more cost-effective.

Numerous studies have shown that matrix-assisted laser desorption ionization-time of flight mass spectrometry (MALDI-TOF MS) is an effective method for the rapid, accurate, and cost-efficient identification of bacterial strains and has been widely implemented in clinical laboratories (17–19). Although this technology has proven useful for species and subspecies identification, its ability to detect bacteria below the subspecies level, such as differentiating serotypes, remains limited (13). A study utilizing a self-constructed mass spectrometry data library successfully identified 28 strains of *Salmonella enterica* subsp. *enterica* ser. Typhimurium (*Salmonella* Typhimurium) and 5 strains of *Salmonella enterica* subsp. *enterica* ser. Enteritidis (*Salmonella* Enteritidis), with accuracy rates of 68% and 20%, respectively (20). Machine learning techniques provide a versatile set of tools for identifying patterns and relationships in complex data and making decisions on the basis of that data. Studies have successfully combined machine learning techniques with MALDI-TOF MS for typing *Enterobacter cloacae* and *Mycobacterium abscessus* (21, 22).

*Salmonella* serotypes A and F are relatively uncommon in clinical practice, whereas *Salmonella* Enteritidis and *Salmonella* Typhimurium are the main serotypes responsible for foodborne infections in humans (23). In this study, eight subtypes were included: B, C1, C2/3, D, E, Not A-F, *Salmonella* Typhimurium, and *Salmonella* Enteritidis. This study aimed to establish and validate an identification model for the automatic differentiation of *Salmonella* serotypes using MALDI-TOF MS and machine learning algorithms. Feature selection was performed using the SHapley Additive exPlanations (SHAP) method, followed by the construction of a streamlined web-based model, providing convenience for clinical laboratories and disease control centers.

## MATERIALS AND METHODS

### Bacterial isolates

**Fig 1:**
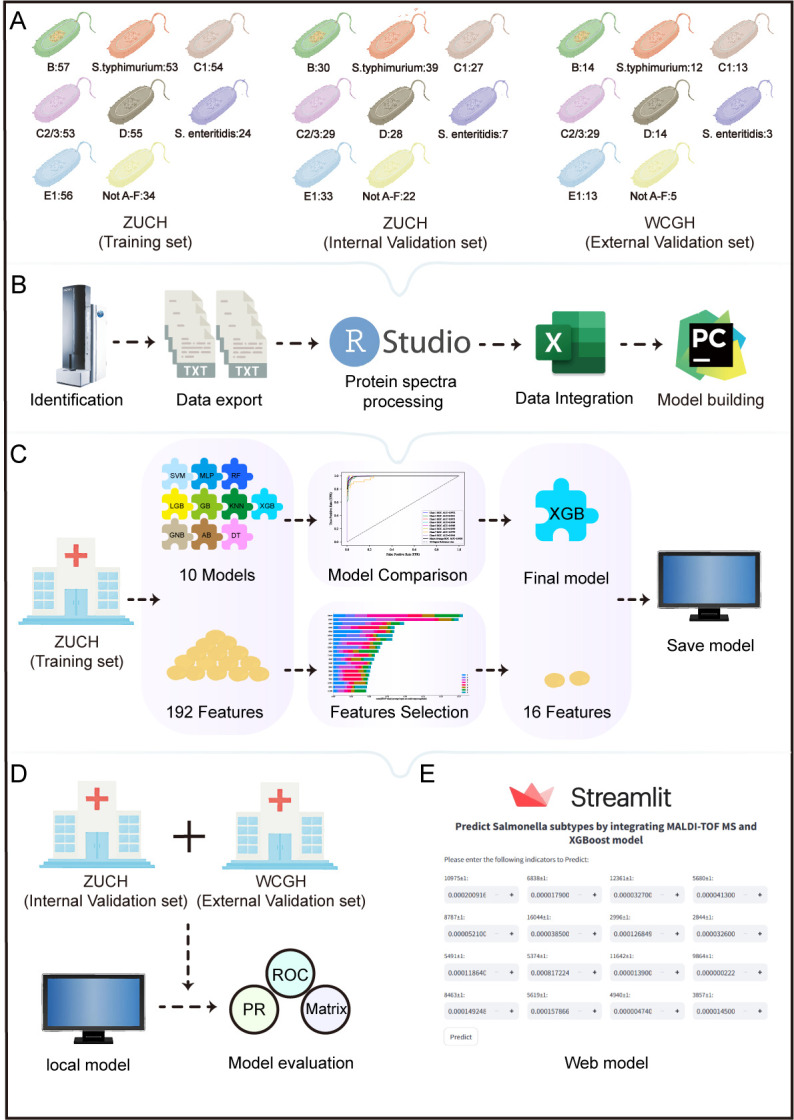
Workflow for *Salmonella* serotype identification via MALDI-TOF MS combined with machine learning. (**A**) Strain sources and study cohort design. (**B**) Acquisition and preprocessing of *Salmonella* mass spectrometry data. (**C**) Development of 10 machine learning models using the training set, followed by feature selection and performance comparison to determine the optimal model. (**D**) Comprehensive evaluation of the model’s performance and generalizability using both internal and external validation sets. (**E**) Deployment of the final model as a web-based application to enhance its applicability.

A total of 692 clinically isolated *Salmonella* strains were included in this study, of which 601 were from the Children’s Hospital, Zhejiang University School of Medicine (ZUCH), and 91 were from the Wanbei Coal-Electricity Group General Hospital (WCGH). The number of strains for the eight *Salmonella* subtypes is detailed in Fig. 1A. All *Salmonella* isolates from ZUCH and WCGH were incubated overnight at 37°C, metabolically activated after one consecutive subculture on *Salmonella*-Shigella agar, and then analyzed using MALDI-TOF MS at their respective hospitals.

### Spectra acquisition by MALDI-TOF MS

The protein extraction procedure for MALDI-TOF MS analysis was carried out as previously described (24). The isolates were identified using a MALDI Biotyper system (Bruker Daltonics, Germany). We applied three spots for each colony to the MALDI target plate. Owing to the limited number of non-A-F subtype samples, we applied four spots for these cases. After the colony was applied to each spot, 1 µL of formic acid was added. Once the sample was dried at room temperature, the matrix solution was applied. Each spot was coated with an HCCA matrix (1 µL, 10 mg/mL in ACN/H_2_O [1:1, vol/vol] with 2% TFA) on the target plate and allowed to air dry at room temperature. The acquisition range for MALDI-TOF MS was set to 2,000–20,000 Da. To build a machine learning model using high-quality mass spectrometry data, we excluded data with identification scores below 2.0.

### Data processing of MALDI-TOF MS protein spectra

Given the variations in protein spectra processing software used by different MALDI-TOF MS manufacturers, we selected the R packages “MALDIquant (version 1.21)” and “MALDIquantForeign (version 1.2)” for analysis (Fig. 1B). This approach makes the protein spectra processing steps more convenient and easier to replicate. First, the data were exported in TXT format and imported into R for processing. A square root transformation (method = “sqrt”) was applied to stabilize the variance of the signal intensities, reducing the influence of extreme values on the analysis. The Savitzky-Golay filter (with a window size of 90) was used to smooth the data, removing noise while preserving the main spectral peaks. Baseline correction was performed using the SNIP method, with 100 iterations to remove systematic baseline drift. Finally, the spectra were aligned using the LOWESS method with a signal-to-noise ratio of 2 and a tolerance of 0.008. Ultimately, 192 protein feature peaks were retained for analysis. The specific data preprocessing code can be found in the supplemental code.

### Isolate identification via serology

*Salmonella* serotyping was performed through serological testing, which was conducted after successful identification of the bacteria using MALDI-TOF. We used a *Salmonella* diagnostic kit (Tian Run, Ningbo) containing 60 different diagnostic sera. One to two drops of serum were placed on a clean glass slide, followed by a small amount of the test colony, which was mixed with the serum. The slide was gently rocked, and the result was observed with the naked eye within 1 min. Sodium chloride solution was used as a negative control. Agglutination within 1 min was considered positive, whereas uniform turbidity indicated a negative result.

### Model development and comparison

The first batch of 1,144 peak lists (*S*. Group B [163], *S*. Group C1 [154], *S*. Group C2/3 [153], *Salmonella* Enteritidis subtype [71], *S*. Group D [159], *S*. Group E1 [162], Non-A-F group [130], and *Salmonella* Typhimurium subtype [152]) was collected from ZUCH. *S*. Group B (86), *S*. Group C1 (78), *S*. Group C2/3 (83), *Salmonella* Enteritidis subtype (20), *S*. Group D (81), *S*. Group E1 (96), Non-A-F group (85), and *Salmonella* Typhimurium subtype (112) were used as the training set, while the second batch of 640 peak lists served as the internal validation set. External validation was conducted using 264 peak lists collected from WCGH (*S*. Group B [41], *S*. Group C1 [39], *S*. Group C2/3 [50], *Salmonella* Enteritidis subtype [9], *S*. Group D [40], *S*. Group E1 [37], Non-A-F group [18], and *Salmonella* Typhimurium subtype [36]).

An identification model was constructed using the 192 protein feature peaks (Fig. 1C). Using median imputation does not artificially reduce variance and helps preserve the original distribution characteristics of the data. Missing data were imputed using the median, and data with more than 10% missing values were excluded. The pandas (version 2.2.3) package was used for imputing missing values in feature peaks, with detailed information provided in the supplemental code. Ten machine learning models were employed for identifying *Salmonella* serotypes, including adaptive boosting (AB), multilayer perceptron (MLP), decision tree (DT), light gradient boost (LGB), k-nearest neighbors (KNN), gradient boosting (GB), random forest (RF), support vector machine (SVM), extreme gradient boosting (XGB), and Gaussian naive Bayes (GNB) methods. The packages (scikit-learn version 1.4.2) used for the 10 models and the detailed code can be found in the supplemental code. To optimize the identification model, a combination of grid search and manual tuning was employed. The average area under the receiver operating characteristic curve (AUC) value from fivefold cross-validation was used as the evaluation metric to determine the optimal hyperparameters for the model. Model performance was evaluated using metrics such as the AUC, sensitivity, specificity, positive predictive value (PPV), negative predictive value (NPV), accuracy, and F1 score. The DeLong nonparametric method was used to compare the models in terms of AUC, further narrowing model selection.

### Feature reduction and model determination

Mass spectrometry data often contain numerous complex features. Feature reduction helps eliminate noise-containing features, reduces model complexity, and enhances model interpretability. SHAP, which is based on game theory, provides the contribution value of each feature to the model output. For the eight-class machine learning model constructed in this study, SHAP can provide insights into the contribution of each feature to the importance of each category. On the basis of feature importance ranking, the identification model was progressively reduced from 192 features to a single feature. As the number of features decreased, the AUC value of the selected model gradually decreased. The DeLong nonparametric method was used to compare the AUC between the full set of 192 features and the reduced feature sets. A *P* value < 0.05 was considered indicative of a statistically significant difference, and the previous reduced feature set was selected as the optimal set for constructing the final identification model. Using the final model, we conducted identifiable analyses for two independent sets, ZUCH (internal validation) and WCGH (external validation), to comprehensively assess the model’s generalizability (Fig. 1D).

### Web-based model deployment

To enhance the model’s usability for clinical *Salmonella* serotyping, we deployed the final identification model as a web application using the Streamlit Python framework (Fig. 1E). In the web app, users interact with the model by inputting values for each feature. These feature values are directly entered into the provided input fields, with each feature corresponding to a specific mass spectrometry data point (the relative intensity of feature peaks). After entering the data, users click the “predict” button, and the application processes the input to calculate the probability for each *Salmonella* serotype, displaying the most likely category along with the probabilities for all possible categories.

### Statistical analysis

Protein spectra processing was performed with R (4.3.2) and RStudio (2023.03.1+446). Machine learning models, ROC curves, decision curve analysis (DCA), precision‒recall (PR) curves, heatmaps, correlation bubble charts, violin plots, and volcano plots were processed using Python (3.11) and PyCharm (2023.3.5). The DeLong test was used to compare the AUC values between different models. The Streamlit application was used for visualizing machine learning models. A two-tailed *P* value < 0.05 was considered statistically significant.

## RESULTS

### Differences in protein spectra among serological subtypes

**Fig 2:**
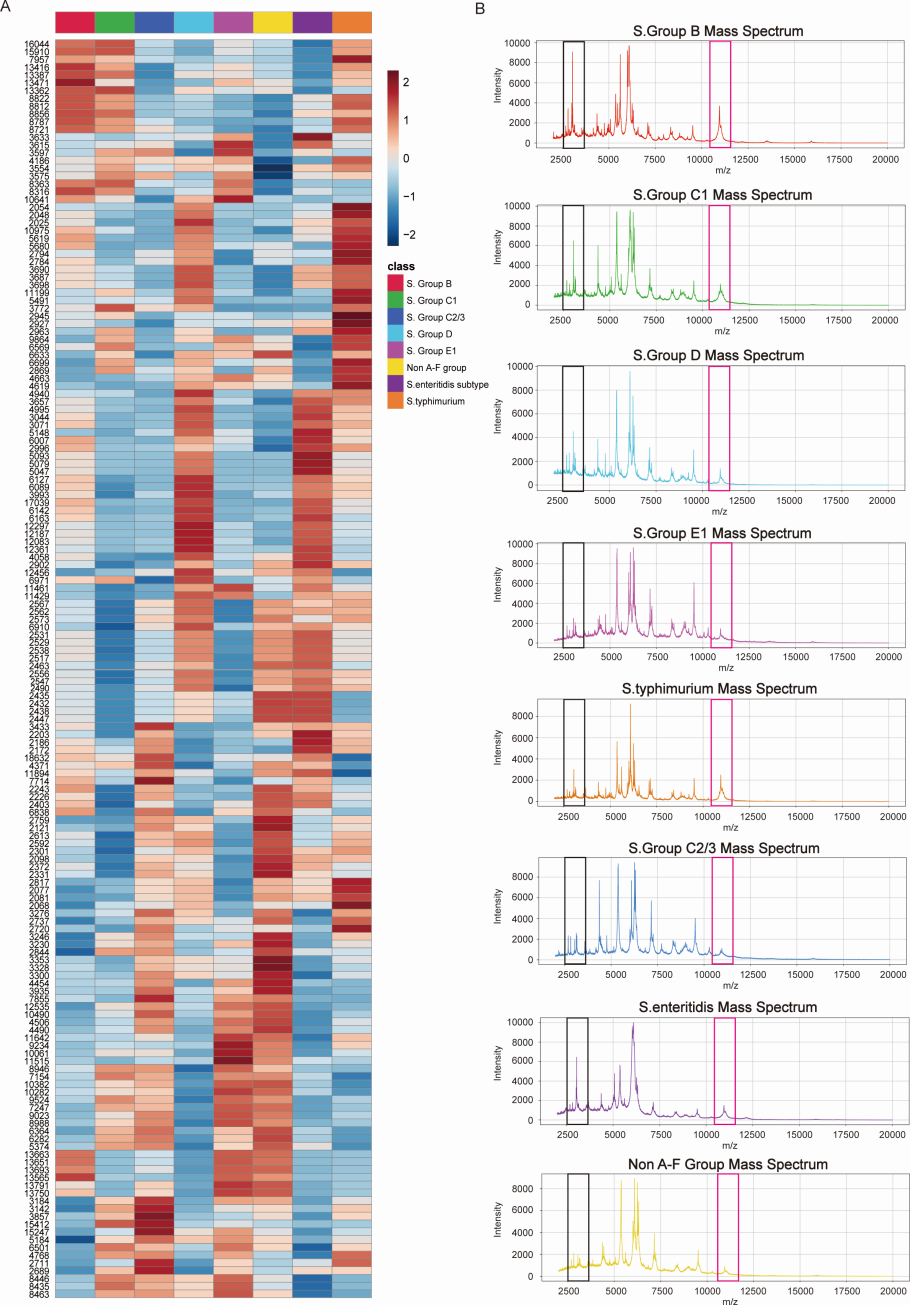
Analysis of the characteristic peak intensities for the eight *Salmonella* serotypes (B, C1, C2/3, D, E, Not A-F, *Salmonella* Typhimurium, and *Salmonella* Enteritidis). (**A**) Heatmap of the intensity distribution of 192 features across the eight *Salmonella* serotypes. The color scale represents the relative intensities of the features. (**B**) MALDI-TOF MS spectra of the eight *Salmonella* serotypes.

A total of 1,144 training set data points, 640 internal validation set data points, and 264 external validation set data points were used to generate the heatmap (Fig. 2A). The data were standardized to eliminate the influence of varying concentration ranges and units of different metabolites. After standardization, the data were transformed into *Z* scores, where the color intensity represents the changes in feature peak intensities: red indicates higher intensity, and blue indicates lower intensity. In the heatmap, the rows represent different *Salmonella* serotypes, and the columns represent different protein feature peaks. By examining the heatmap, significant differences in protein feature peak intensities between different *Salmonella* serotypes can be clearly observed, indicating specific changes in protein expression levels across serotypes. As shown in Fig. 1B, we generated mass spectrometry profiles for the eight *Salmonella* serotypes and highlighted feature peaks with significant intensity variations. To further analyze these differences, we performed unsupervised principal component analysis (PCA) and t-distributed stochastic neighbor embedding (t-SNE) on the basis of the intensity changes in the feature peaks. The results showed that the points for different serotypes in the PCA (Fig. S1A) and t-SNE (Fig. S1B) plots formed distinct clusters, highlighting specific changes in protein expression across different *Salmonella* serotypes. The above results demonstrate the potential of protein feature peaks in differentiating *Salmonella* serotypes.

### Model development and performance comparison

**Fig 3:**
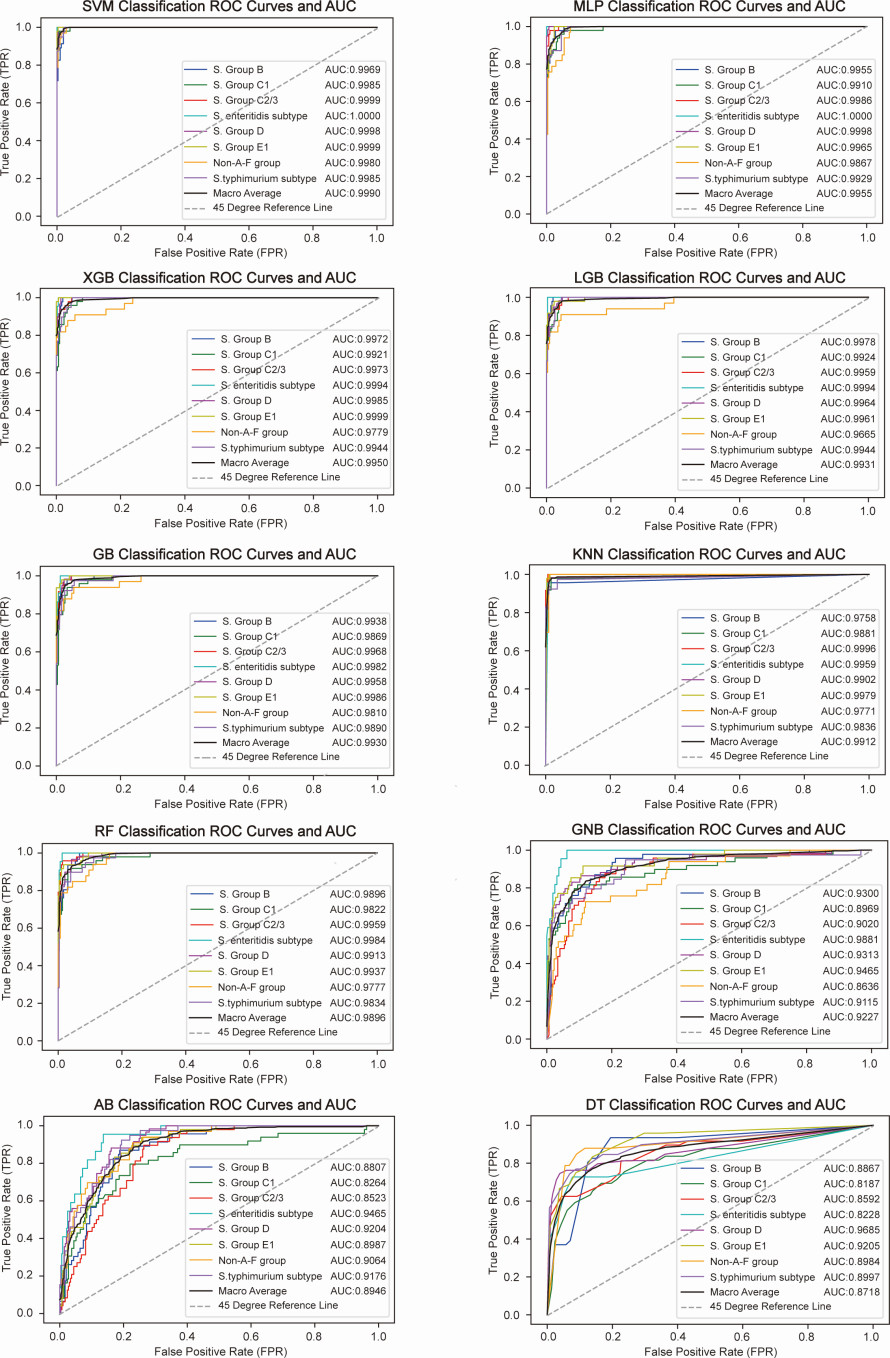
ROC curves for identifying *Salmonella* serotypes using 10 machine learning models (AB, MLP, DT, LGB, KNN, GB, RF, SVM, XGBoost, and GNB).

The training set data (192 features) were used to develop 10 machine learning models to identify *Salmonella* serotypes (Fig. 3). The PR curves and confusion matrix heatmaps for the 10 models are shown in Fig. S2 and S3. The average AUC, sensitivity, specificity, PPV, NPV, accuracy, and F1 score for the eight serotypes are shown in Table 1. The parameters of the 10 models are provided in Table S1. The evaluation metrics for each individual serotype can be found in Table S2. To optimize model selection, we performed DeLong’s test to compare the AUC values of each model and calculated the *P* values for the differences between the model AUCs (Table S3). Through this process, models that were statistically significantly inferior were eliminated, retaining only the SVM, MLP, and XGB models, which demonstrated the best identification performance.

**TABLE 1:** The identifiable performance of machine learning models across different data sets^*a*^

	Models	AUC	Sensitivity	Specificity	PPV	NPV	Accuracy	F1 score
Train set (192 features)	RF	0.9895	0.89	0.98	0.88	0.98	0.89	0.88
	AB	0.8946	0.60	0.95	0.71	0.95	0.71	0.61
	DT	0.8718	0.63	0.95	0.64	0.95	0.64	0.62
	GB	0.993	0.90	0.98	0.89	0.98	0.90	0.89
	KNN	0.9912	0.92	0.99	0.92	0.99	0.92	0.92
	NB	0.9227	0.67	0.95	0.65	0.95	0.67	0.66
	MLP	0.9955	0.90	0.99	0.90	0.99	0.90	0.89
	XGB	0.995	0.92	0.99	0.92	0.99	0.92	0.92
	LGB	0.9931	0.90	0.99	0.90	0.99	0.90	0.90
	SVM	0.999	0.96	0.99	0.96	0.99	0.96	0.96
Train set (16 features)	XGB	0.9898	0.88	0.98	0.88	0.98	0.88	0.88
	SVM	0.9981	0.87	0.98	0.87	0.87	0.87	0.87
	MLP	0.9293	0.68	0.95	0.68	0.95	0.68	0.68
Internal validation set (16 features)	XGB	0.9662	0.8	0.97	0.82	0.97	0.95	0.82
	SVM	0.9398	0.65	0.95	0.7	0.95	0.91	0.63
	MLP	0.8769	0.53	0.93	0.55	0.93	0.55	0.52
External validation set (16 features)	XGB	0.9778	0.79	0.97	0.77	0.96	0.94	0.77
	SVM	0.9735	0.83	0.97	0.85	0.97	0.96	0.84
	MLP	0.9466	0.72	0.93	0.72	0.96	0.72	0.71

^*a*^ The evaluation metrics are the averages calculated based on the identification results of eight *Salmonella* serotypes.

### Feature reduction and final model determination

**Fig 4:**
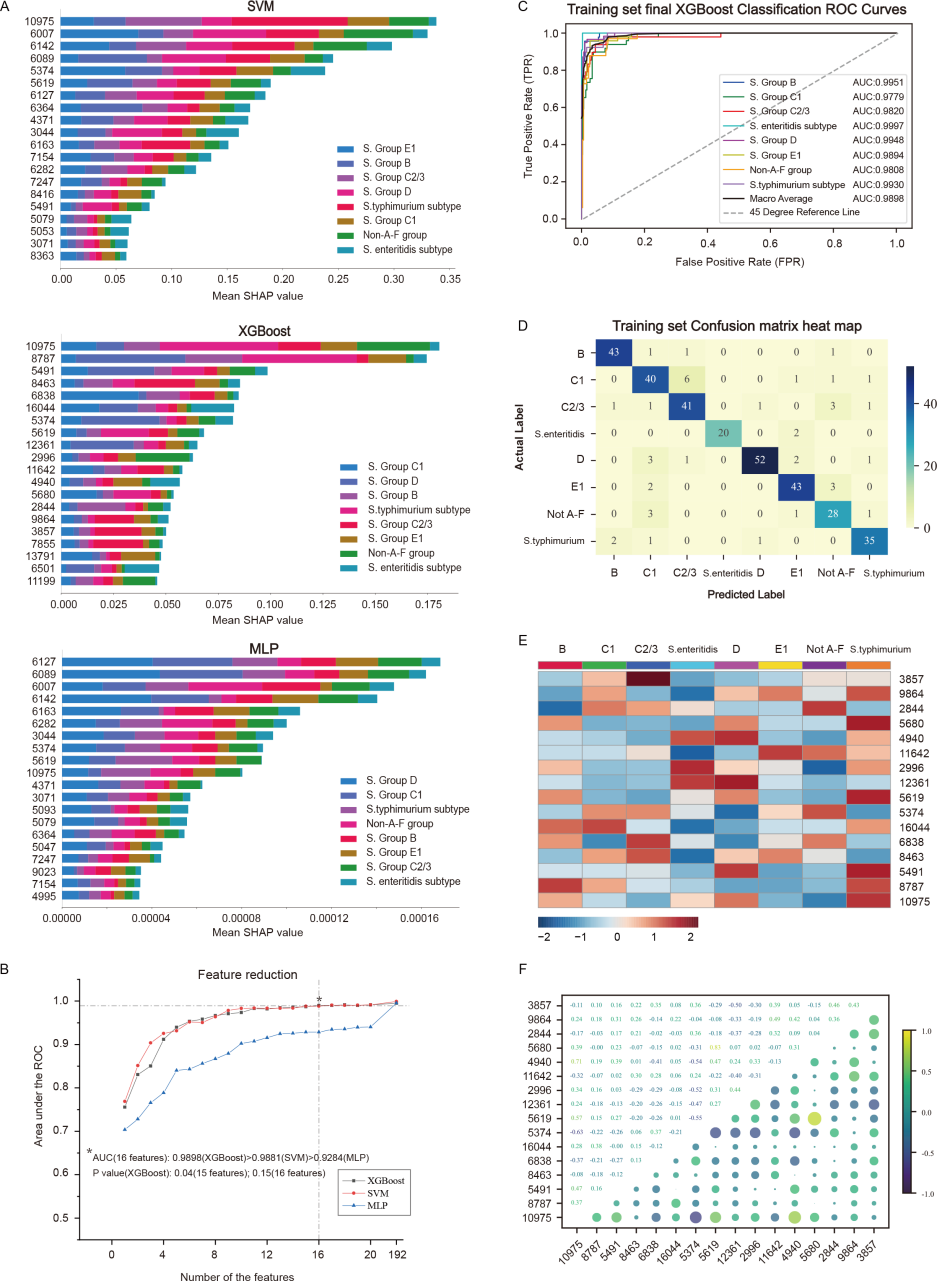
Model comparison and feature selection. (**A**) SHAP summary bar plots for the SVM, XGB, and MLP models. For each feature, the varying lengths and colors of the bars represent the contributions to different serotypes. (**B**) AUCs of the SVM, XGB, and MLP models with various numbers of features. The ROC curves (**C**) and confusion matrix heatmaps (**D**) of the final XGB model with 16 features in the training set. (**E**) Heatmap of the intensities of 16 characteristic peaks across different *Salmonella* serotypes for the training set. The color scale represents the relative intensities of the features. (**F**) Bubble plot of correlations among the 16 features in the training set.

The SHAP summary bar plots for the top 20 features of the best-performing SVM, MLP, and XGB models are shown in Fig. 4A. Each feature is represented using eight different colors to illustrate the contribution of each of the eight serotypes, providing a clear visualization of the impact of various features on different serotypes. For example, in the XGB model, 10,975 and 8,787 *m*/*z* contributed the most to the classification of *Salmonella* Typhimurium, 5,491 *m*/*z* contributed the most to serotype D, 8,463 *m*/*z* contributed the most to serotypes C2/3, and 6,838 *m*/*z* contributed the most to serotype C1. Different features play key roles in distinguishing between different serotypes. During the feature reduction process based on feature importance, the AUC changes of these three models were observed, showing that the SVM and XGB models maintained good identification performance even with reduced features, demonstrating their stability and robustness in the feature selection process (Fig. 4B).

For the XGB model, when the number of features was reduced from 192 to 15, there was a statistically significant difference in the AUC values between the two models (AUC: 0.9995 vs 0.9869, *P* = 0.04). Therefore, the model with 16 features was considered the optimal feature model (AUC: 0.9995 vs 0.9898, *P* = 0.15). Compared with the 192-feature model, the 16-feature model demonstrated better net benefit and a higher threshold probability. The ROC curve and confusion matrix heatmap of the training set for the 16-feature XGB model are shown in Fig. 4C and D. The area under the PR curve for the training set for the 16-feature model was only slightly lower than that of the 192-feature model, indicating that both models have similar and high clinical utility (Fig. S4A). For the SVM model, when the number of features was reduced from 192 to 20, a statistically significant difference in AUC values was observed between the two models (AUC: 0.9995 vs 0.9913, *P* = 0.001). When the number of features was 16, the AUC value of the XGB model (0.9898) was greater than that of the SVM model (0.9881). For detailed information, see Table S4. The ROC curves, PR curves, and confusion matrix heatmaps from the training set for the 16-feature SVM and MLP models are shown in Fig. S5 and S6. Therefore, we selected the 16-feature XGB model, which consists of features 10,975, 8,787, 5,491, 8,463, 6,838, 16,044, 5,374, 5,619, 12,361, 2,996, 11,642, 4,940, 5,680, 2,844, 9,864, and 3,857 *m*/*z*, for further analysis.

In the training set, the expression levels of 16 features exhibited significant differences across different serotypes, and the observed variations were generally consistent with the contribution levels identified in the SHAP feature importance analysis (Fig. 4E). These findings indicate that the expression levels of these features not only play a role in distinguishing between different serotypes but are also closely related to the importance of these features for model identification, as reflected by the SHAP analysis. This consistency confirms the interpretability of the model. Figure 4F shows the correlation between the 16 features, with some features having higher correlations due to similar *m*/*z* values. These features may exhibit similar variation patterns or interdependent relationships in the validation set. The violin plot shows the overall distribution of 16 features across eight serotypes in the training set, providing a visual representation of the data distribution and variation in each feature across different serotypes (Fig. S7A). A volcano plot was used to display the statistically significant upregulated and downregulated features in each serotype, highlighting the features that exhibited significant changes across serotypes (Fig. S7B).

### Internal and external validation of the final model

For the internal validation set, the final model achieved an AUC of 0.9662, which was similar to that for the training set (*Δ*AUC = 0.0236). For the external validation set, the final model achieved an AUC of 0.9778, which was also similar to that for the training set (*Δ*AUC = 0.012). These results indicate that the final model demonstrated strong performance across both internal and external validations.

**Fig 5:**
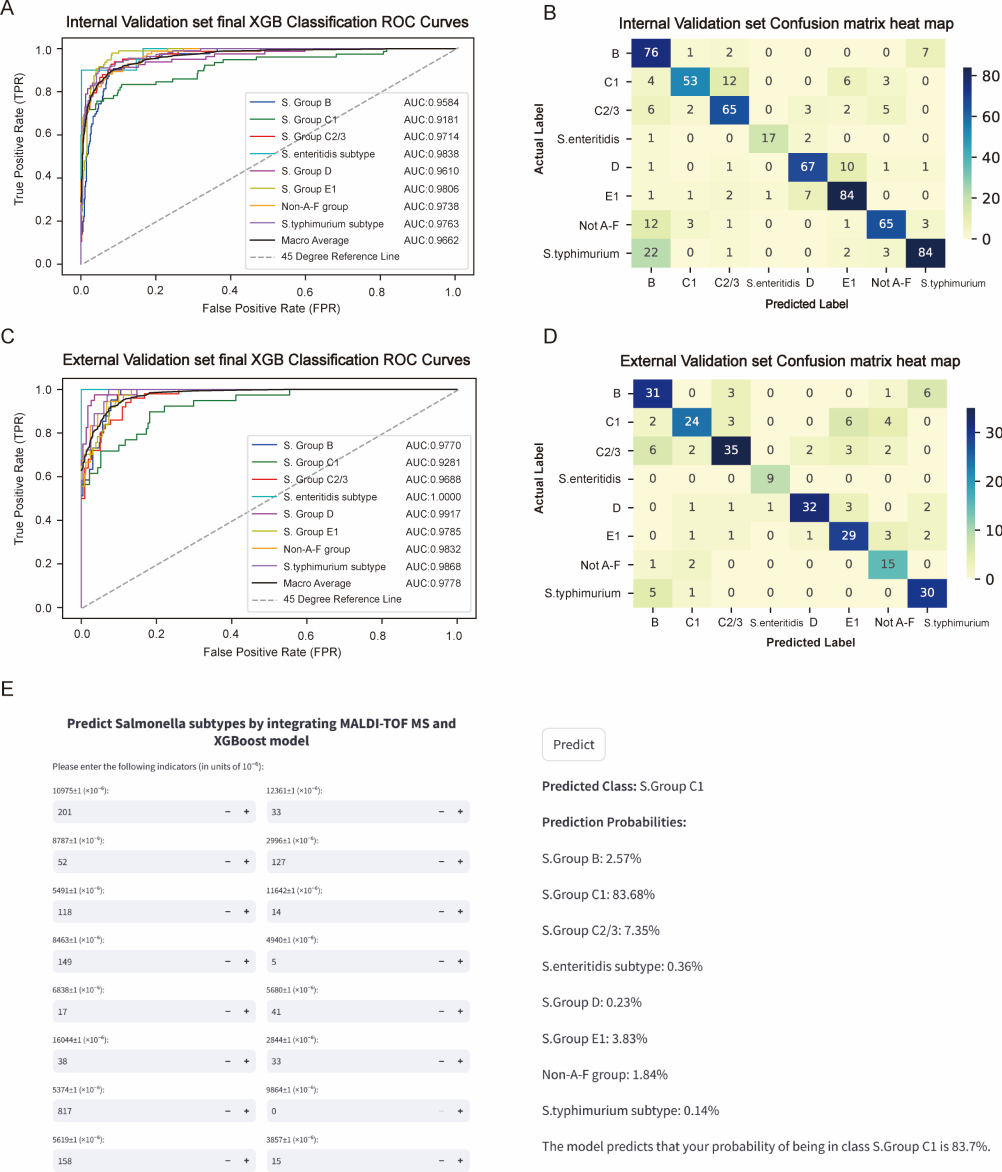
Final model’s generalizability and visualization analysis. The ROC curve (**A**) and confusion matrix heatmap (**B**) of the final XGB model with 16 features for the internal validation set. The ROC curve (**C**) and confusion matrix heatmap (**D**) of the final XGB model with 16 features for the external validation set. (**E**) A convenient application of the final XGB model with 16 features is available for *Salmonella* serotype identification.

The ROC curves and confusion matrix heatmaps for the internal and external validation sets are shown in Fig. 5A through D. The PR curve is presented in Fig. S4. Although the feature strength heatmaps, bubble plots, and violin plots for the internal and external validation sets were generally consistent with the results from the training set, reflecting the stability and importance of the features, there were differences in the feature rankings (Fig. S8A through C and Fig. S9A through C). Specifically, the volcano plots revealed differential features in the internal and external validation sets that differed from those in the training set, further indicating the model’s generalizability and adaptability to different data sets (Fig. S8D and S9D). This difference also indirectly suggests that, unlike volcano plots, the SHAP feature ranking method more accurately ranks features, making it more suitable for feature selection in machine learning models.

The ROC curves, PR curves, and confusion matrix heatmaps for SVM and MLP using the internal and external validation sets are shown in Fig. S5 and S6. The results indicate that the performance of the XGB model was superior to that of the SVM and MLP models. In the evaluation of the 16 features across the training, internal validation, and external validation sets for the eight serotypes, the average AUC, sensitivity, specificity, PPV, NPV, accuracy, and F1 score for XGB, SVM, and MLP are presented in Table 1. The detailed evaluation metrics for each category can be found in Tables S5 to S7.

DCA for the final model revealed consistent net benefits across the threshold probability range in the training, internal validation, and external validation sets. In all three cohorts, the performance of the XGB model (16 features) was superior to that of both the “no treatment” and “treat all” strategies, further demonstrating the model’s effectiveness and practical value in decision-making (Fig. S10 to 12).

### Model visualization

We implemented the final identification model in a web application, as shown in Fig. 5E. When users input the actual values of the 16 features required by the model, the application automatically identifies the *Salmonella* serotype and outputs the identification probabilities for each serotype, including *S*. Group B, *S*. Group C1, *S*. Group C2/3, *Salmonella* Enteritidis subtype, *S*. Group D, *S*. Group E1, Non-A-F group, and *Salmonella* Typhimurium subtype. Additionally, the application displays the category with the highest identification probability as the final result. This web application is now live and can be accessed online via the following link: https://predict-salmonella-subtypes.streamlit.app/. The web application URL may occasionally display a “sleeping” status. Please wait for approximately 30 seconds, and the link will automatically reactivate.

## DISCUSSION

Identification of *Salmonella* serotypes is crucial for clinical decision-making, especially for personalized treatment and antibiotic selection. While most *Salmonella* serogroups cause gastroenteritis, some nontyphoidal serotypes, such as *S*. cholerae-suis (C1) and *S*. dublin (D), are more likely to cause bacteremia (25). Early identification is key for systemic infection detection and treatment. Additionally, significant differences in antibiotic resistance exist between serotypes. For example, *Salmonella* Enteritidis showed 88.0% moderate resistance to ciprofloxacin in Korea (26). In China, a unique multidrug-resistant *Salmonella* Enteritidis clone has emerged, which may impact treatment protocols (27). In summary, serotype determination aids clinicians in selecting appropriate treatments and antibiotics.

To our knowledge, this study is the first cross-sectional, multicenter study that focuses on investigating and comparing 10 machine learning models for *Salmonella* serotype identification analysis. On the basis of the MALDI-TOF MS data, we selected 16 protein features using the SHAP method and constructed the final identification model using the filtered XGB model. Among the 16 features, a few have relatively high correlations (e.g., 10,975 and 4,940 have a correlation of 0.71, and 5,619 and 5,680 have a correlation of 0.83), but owing to the small number of correlated features, they were not removed. Although highly correlated features could increase redundancy, they make significant contributions to the model’s prediction, and removing them could affect the model’s accuracy and generalizability. The XGB model accurately identified isolates of eight *Salmonella* serotypes (*S*. Group B, *S*. Group C1, *S*. Group C2/3, *Salmonella* Enteritidis subtype, *S*. Group D, *S*. Group E1, Non-A-F group, and *Salmonella* Typhimurium subtype). For the data from the ZUCH training set cohort, the model achieved an AUC of 0.9898; for the data from the ZUCH internal validation cohort, the AUC was 0.9662; and for the data from the WCGH external validation cohort, the AUC was 0.9778, indicating that the model demonstrated high accuracy across different data sets. We determined the optimal thresholds for the *Salmonella* serotypes by balancing sensitivity and specificity, with the following values: B serotype (optimal cutoff: 0.46, sensitivity: 0.9370, and specificity: 0.8865), C1 serotype (optimal cutoff: 0.47, sensitivity: 0.8034, and specificity: 0.8942), C2/3 serotype (optimal cutoff: 0.48, sensitivity: 0.9398, and specificity: 0.8869), *Salmonella* Enteritidis serotype (optimal cutoff: 0.59, sensitivity: 0.9310, and specificity: 0.9989), D serotype (optimal cutoff: 0.36, sensitivity: 0.9256, and specificity: 0.9354), E1 serotype (optimal cutoff: 0.47, sensitivity: 0.9774, and specificity: 0.9049), Non-A-F serotype (optimal cutoff: 0.42, sensitivity: 0.9223, and specificity: 0.9010), and *Salmonella* Typhimurium serotype (optimal cutoff: 0.47, sensitivity: 0.9459, and specificity: 0.9148). In actual clinical practice, these thresholds must be adjusted on the basis of expert advice to balance sensitivity and specificity, thus avoiding over-screening and missed diagnoses. This ensures the practical application of the model in different clinical settings.

The reliability of MALDI-TOF MS detection depends on the quality and completeness of the reference spectra in the database (28). Commercial databases have limited identification capabilities for subspecies or low classifications (29). Using self-built databases and adding new reference spectra enhanced the detection reliability and improved the identification accuracy for more detailed classifications (30). Studies have used self-built databases to identify 82 clinical isolates of *Salmonella*, and the results indicated that the identification accuracy of MALDI-TOF MS at the species level reached 98%, but the accuracy at the serotype level was only 20%–68% (20). Although self-built databases increase the accuracy of *Salmonella* serotype identification, their operation is complex and requires experienced personnel, with accuracy often falling short of clinical requirements. Therefore, integrating machine learning models as an innovative tool has become an effective solution that further improves identification accuracy and automation, meeting the demands of clinical applications.

Machine learning technology is a powerful computational method used to handle complex and large-scale data sets (31). It facilitates the handling of highly variable data and makes it easier to understand the complex relationships between variables in a flexible and trainable way. Among the 10 machine learning models evaluated, the XGB model performed the best, with significant net benefits and high stability in threshold probability for feature reduction. The XGB model is an ensemble learning method that involves constructing multiple decision trees and combining them to progressively optimize the identification results (32). Numerous studies have demonstrated the excellent predictive value of the XGB model in the medical field, particularly in disease prediction, risk assessment, and classification tasks (32, 33). Owing to the large number of bacterial protein peaks, determining how many features should be included in the model is difficult. While more features can provide more information for the identification model, an excessive number of redundant features may reduce the model’s identification accuracy and limit its practical application in clinical settings. Therefore, we employed the SHAP method for feature selection to identify the most contributive features, thereby improving the model’s identification performance and ensuring its clinical interpretability and operability. Different machine learning models have varying architectures and feature selection methods, leading to differences in feature importance rankings. For example, XGB, a tree-based model, prioritizes features on the basis of data splits, whereas the SVM and MLP models rely on linear separability or neural network weights, which can explain why peak 6,007 ranks highly in SVM and MLP but not in XGB. In this study, we developed a final model with 16 features using the XGB model, which achieved high identification accuracy and stability and can be effectively applied to the serotyping analysis of *Salmonella*.

Owing to the existence of batch effects in mass spectrometry, the reproducibility and generalizability of the model could potentially be compromised. To address this concern, we employed data alignment and baseline correction using the R package maldiquant, ensuring that the feature extraction process was consistent across different batches. This preprocessing step minimized the technical variation between samples from different batches and helped reduce batch-specific bias. To further enhance the generalizability and reliability of the model, we not only collected ZUCH *Salmonella* data for model training but also independently collected an additional batch of data from ZUCH for internal validation, while using WCGH data as an external validation set. The results of the validation indicated that the AUCs for both the internal and external validation sets were similar to those of the training set, demonstrating good reproducibility and stability across different batches from the same center as well as between centers. These findings suggest that the XGBoost model is capable of mitigating batch effects, maintaining strong cross-center generalizability. Notably, the AUC of the MLP model for the external validation set was slightly greater than that for the training set. This may be attributed to the relatively small size of the external validation set, where the model could generalize better at a lower complexity, resulting in improved performance.

In this study, the preliminary model showed poorer performance for non-A-F serotypes because of their heterogeneity and limited representation in the training set. These serotypes are more diverse with greater feature variability, making them harder to identify. However, after feature selection, the final XGB model identified 16 key features that improved robustness and generalizability, including non-A-F serotypes. Future studies could expand the training set with more diverse non-A-F samples to further enhance the predictive capability for these groups.

Misclassification is a common issue in machine learning models and can arise from multiple factors. First, overlapping spectral features may be a primary cause of misclassification, especially when the MS peaks of different serotypes are similar. For example, *Salmonella* Typhimurium belongs to group B, and some mass spectrometry peaks between it and other B group *Salmonella* may be similar, leading to errors in distinguishing these serotypes. Second, insufficient training data may also affect the model’s performance, as it might not learn enough distinguishing features. For example, the C1 and C2/3 serotypes may be misclassified as multiple other serotypes. To reduce misclassification and improve model accuracy, incorporating more samples for training in the future is essential.

We acknowledge several limitations in this study. First, since the model was built using *Salmonella* data from China, its generalizability for distinguishing *Salmonella* serotypes globally remains unclear, and further studies are needed to evaluate its performance in different regions and strains. Second, the data from ZUCH and WCGH were obtained using Bruker’s microbial MALDI-TOF MS technology. Therefore, the applicability of this model to microbial MALDI-TOF MS data generated by other systems, such as bioMérieux, requires validation. Finally, the model in this study shows good discriminative ability for *Salmonella* serotypes in both the training and validation sets but may have limitations with other serotypes (serotype A, *S. typhi*) not included in the training data. These serotypes not included in the training set are typically misclassified with low probability values as one of the eight serogroups in this study. Future work will focus on expanding the training data set by including more *Salmonella* strains from diverse serotypes, especially those not covered in this study, to improve model accuracy and reliability.

In conclusion, we successfully developed an interpretable XGB model for identifying *Salmonella* serotypes on the basis of easily extractable feature data from MALDI-TOF MS. The final model demonstrated a strong ability to identify *Salmonella* serotypes via both internal and external validation, indicating high accuracy and stability. Additionally, we deployed an open web application available for free use by clinicians and relevant professionals, aiming to support rapid diagnosis of *Salmonella* serotypes and promote the application of this technology in clinical practice. Compared with other studies using MALDI-TOF and machine learning, this study provides detailed steps and codes for MALDI-TOF MS data preprocessing and machine learning, employs SHAP for model interpretation, and offers a framework for building a web-based prediction model. These innovations significantly enhance the transparency and practicality of this work.

## Data Availability

The MALDI-TOF MS data of Salmonella are available upon reasonable request from the corresponding author. The analysis code associated with this article is available on GitHub (https://github.com/fudanRenjun/shamen/tree/master).

## SUPPLEMENTAL MATERIAL

Supplemental material jcm.00037-25-s0001.docx Tables S1 to S7, Fig. S1 to S12, and Code.

ASM does not own the copyrights to Supplemental Material that may be linked to, or accessed through, an article. The authors have granted ASM a non-exclusive, world-wide license to publish the Supplemental Material files. Please contact the corresponding author directly for reuse.
