# Supplementary material for: Automated identification of Salmonella serotype using MALDI-TOF mass spectrometry and machine learning techniques
Source: J Clin Microbiol. 2025 Jun 11;63(7):e00037-25. doi: 10.1128/jcm.00037-25 (PMC12239726; doi:10.1128/jcm.00037-25)
Supplement: Supplemental material — Tables S1 to S7, Fig. S1 to S12, and Code. [file jcm.00037-25-s0001.docx]

**Supplementary Material**

1. Table S1: List of parameters for 10 models.

2. Table S2: Evaluation of identifiable performance for 10 machine learning models on the training set (containing 192 features). The identification s is specific to 8 *Salmonella* serotypes. — Pages 4-5

33. Table S3: The differences in AUC among the 10 models were compared using Delong's nonparametric method. — Pages 6-7

4. Table S4: The performance of XGB, SVM, and MLP models with varying numbers of features for identifying *Salmonella* serotypes on the validation set. — Pages 8-9

5. Table S5: The performance of the XGB model (16 features) in identifying *Salmonella* serotypes on the training set, internal validation set, and external validation set. — Page 10

6. Table S6: The performance of the MLP model (16 features) in identifying *Salmonella* serotypes on the training set, internal validation set, and external validation set. — Page 11

7. Table S7: The performance of the SVM model (16 features) in identifying *Salmonella* serotypes on the training set, internal validation set, and external validation set. — Page 12

8. Figure S1：PCA(A) and t-SNE(B) were used for the feasibility study of *Salmonella* serotype classification based on mass spectrometry data. — Page 13

9. Figure S2: PR curves for identifying *Salmonella* serotypes using 10 machine learning models. — Page 14

10. Figure S3: Confusion matrix heat maps for identifying *Salmonella* serotypes using 10 machine learning models. — Page 15

11. Figure S4: The PR curves of the final XGB model with 16 features on the training set. — Page 16

12. Figure S5: The ROC curves, PR curves and confusion matrix heat maps of the MLP model with 16 features on the training set, internal validation set, and external validation set. — Page 17

13. Figure S6: The ROC curves, PR curves and confusion matrix heat maps of the SVM model with 16 features on the training set, internal validation set, and external validation set. — Page 18

14. Figure S7: Description of features in the training set. The violin plot showed the overall distribution of 16 features across 8 serotypes in the training set (A). The volcano plot was used to display statistically significant upregulated and downregulated features in each serotype (B). — Page 19

15. Figure S8: Description of features in the internal validation set. Heatmap of the intensities of 16 characteristic peaks across different *Salmonella* serotypes in the internal validation set (A). Bubble chart of the correlations among the 16 features in the internal validation set (B). The violin plot showed the overall distribution of 16 features across 8 serotypes in the internal validation set (C). The volcano plot was used to display statistically significant upregulated and downregulated features in each serotype (D). — Page 20

16. Figure S9: Description of features in the external validation set. Heatmap of the intensities of 16 characteristic peaks across different *Salmonella* serotypes in the external validation set (A). Bubble chart of the correlations among the 16 features in the external validation set (B). The violin plot showed the overall distribution of 16 features across 8 serotypes in the external validation set (C). The volcano plot was used to display statistically significant upregulated and downregulated features in each serotype (D). — Page 21

17. Figure S10: The DCA of the final XGB model (16 features) in the training set, showing the identification performance for 8 *Salmonella* serotypes. — Page 22

18. Figure S11: The DCA of the final XGB model (16 features) in the internal validation set, showing the identification performance for 8 *Salmonella* serotypes. — Page 23

19. Figure S12: The DCA of the final XGB model (16 features) in the external validation set, showing the identification performance for 8 *Salmonella* serotypes. — Page 24

20. Code

(1) Data processing of MALDI-TOF MS protein spectra— Page 25-26

(2) Missing value imputation — Page 27

(3) Ten machine learning models — Page 28-67

(4) Model interpretability— Page 68

1. Table S1: List of parameters for 10 models.

|  | Parameter | Settings |
| --- | --- | --- |
| AB | learning_rate | 0.2 |
|  | n_estimators | 100 |
|  | algorithm | SAMME.R |
| MLP | activation | relu |
|  | alpha | 0.0001 |
|  | hidden_layer_sizes | (50,) |
|  | learning_rate | constant |
|  | solver | Sgd |
| DT | criterion | entropy |
|  | max_depth | 5 |
|  | min_samples_leaf4 | 4 |
|  | min_samples_split | 2 |
| LGB | num_leaves | 31 |
|  | learning_rate | 0.05 |
|  | feature_fraction | 0.9 |
|  | bagging_fraction | 0.8 |
|  | bagging_freq | 5 |
| KNN | n_neighbors | 3 |
|  | weights | distance |
| GB | max_depth | 5 |
|  | min_samples_leaf | 10 |
|  | min_samples_split | 5 |
|  | n_estimators | 50 |
| RF | max_depth | 7 |
|  | min_samples_split | 2 |
|  | min_samples_leaf | 1 |
|  | random_state | 42 |
| SVM | C | 100 |
|  | gamma | Scale |
|  | kernel | rbf |
| XGB | colsample_bytree | 0.5 |
|  | learning_rate | 0.2 |
|  | max_depth | 33 |
|  | n_estimators | 100 |
| GNB | var_smoothing | 1e-07 |

Table S2: Evaluation of identifiable performance for 10 machine learning models on the training set (containing 192 features). The identification s is specific to 8 *Salmonella* serotypes.

|  | Evaluation indexes | S. Group B | S. Group C1 | S. Group C2/3 | *Salmonella* Enteritidis | S. Group D | S. Group E1 | Non-A-F group | *Salmonella* Typhimurium |
| --- | --- | --- | --- | --- | --- | --- | --- | --- | --- |
| RF | AUC | 0.9896 | 0.9822 | 0.9959 | 0.9984 | 0.9913 | 0.9937 | 0.9777 | 0.9834 |
|  | Sensitivity | 0.96 | 0.88 | 0.94 | 0.95 | 0.90 | 0.85 | 0.82 | 0.79 |
|  | specificity | 0.96 | 0.98 | 0.99 | 0.99 | 0.99 | 1.00 | 0.98 | 0.98 |
|  | PPV | 0.80 | 0.88 | 0.92 | 0.91 | 0.95 | 0.95 | 0.82 | 0.84 |
|  | NPV | 0.99 | 0.98 | 0.99 | 1.00 | 0.98 | 0.98 | 0.98 | 0.97 |
|  | accuracy | 0.80 | 0.88 | 0.92 | 0.91 | 0.95 | 0.98 | 0.82 | 0.84 |
|  | F1 score | 0.87 | 0.88 | 0.94 | 0.95 | 0.90 | 0.85 | 0.82 | 0.79 |
| AdaBoost | AUC | 0.8807 | 0.8264 | 0.8523 | 0.9465 | 0.9204 | 0.8987 | 0.9064 | 0.9176 |
|  | Sensitivity | 0.80 | 0.57 | 0.83 | 0.55 | 0.80 | 0.44 | 0.18 | 0.67 |
|  | specificity | 0.90 | 0.93 | 0.84 | 1.00 | 0.98 | 0.97 | 1.00 | 0.96 |
|  | PPV | 0.55 | 0.57 | 0.45 | 0.92 | 0.87 | 0.72 | 0.86 | 0.70 |
|  | NPV | 0.97 | 0.93 | 0.97 | 0.97 | 0.96 | 0.91 | 0.92 | 0.96 |
|  | accuracy | 0.55 | 0.57 | 0.45 | 0.92 | 0.87 | 0.72 | 0.86 | 0.70 |
|  | F1 score | 0.80 | 0.57 | 0.83 | 0.55 | 0.80 | 0.44 | 0.18 | 0.67 |
| DT | AUC | 0.8867 | 0.8187 | 0.8592 | 0.8228 | 0.8685 | 0.9205 | 0.8984 | 0.8997 |
|  | Sensitivity | 0.50 | 0.55 | 0.54 | 0.50 | 0.71 | 0.67 | 0.85 | 0.74 |
|  | specificity | 0.92 | 0.94 | 0.98 | 0.97 | 0.97 | 0.96 | 0.91 | 0.93 |
|  | PPV | 0.48 | 0.60 | 0.84 | 0.52 | 0.84 | 0.74 | 0.50 | 0.58 |
|  | NPV | 0.92 | 0.93 | 0.93 | 0.97 | 0.94 | 0.95 | 0.98 | 0.97 |
|  | accuracy | 0.48 | 0.60 | 0.84 | 0.52 | 0.84 | 0.74 | 0.50 | 0.58 |
|  | F1 score | 0.49 | 0.57 | 0.66 | 0.51 | 0.77 | 0.70 | 0.63 | 0.65 |
| GBoost | AUC | 0.9938 | 0.9869 | 0.9968 | 0.9982 | 0.9958 | 0.9986 | 0.9810 | 0.9890 |
|  | Sensitivity | 0.91 | 0.99 | 0.94 | 0.95 | 0.88 | 0.92 | 0.82 | 0.82 |
|  | specificity | 0.97 | 0.96 | 0.99 | 0.99 | 0.99 | 1.00 | 0.99 | 0.98 |
|  | PPV | 0.84 | 0.80 | 0.92 | 0.88 | 0.95 | 1.00 | 0.93 | 0.84 |
|  | NPV | 0.99 | 0.98 | 0.99 | 1.00 | 0.98 | 0.99 | 0.98 | 0.98 |
|  | accuracy | 0.84 | 0.80 | 0.92 | 0.88 | 0.95 | 1.00 | 0.93 | 0.84 |
|  | F1 score | 0.88 | 0.85 | 0.93 | 0.91 | 0.91 | 0.96 | 0.87 | 0.83 |
| KNN | 0.9758 | 0.9758 | 0.9881 | 0.9996 | 0.9959 | 0.9902 | 0.9979 | 0.9971 | 0.9836 |
|  | 0.913 | 0.91 | 0.98 | 0.92 | 0.95 | 0.95 | 0.98 | 0.76 | 0.92 |
|  | 0.9933 | 0.99 | 0.98 | 0.99 | 0.99 | 0.99 | 0.99 | 0.99 | 0.99 |
|  | 0.9545 | 0.95 | 0.91 | 0.96 | 0.84 | 0.95 | 0.94 | 0.89 | 0.92 |
|  | 0.9867 | 0.99 | 1.00 | 0.99 | 1.00 | 0.99 | 1.00 | 0.97 | 0.99 |
|  | 0.95 | 0.95 | 0.91 | 0.96 | 0.84 | 0.95 | 0.94 | 0.89 | 0.92 |
|  | 0.93 | 0.93 | 0.94 | 0.94 | 0.89 | 0.95 | 0.96 | 0.82 | 0.92 |
| NB | 0.9300 | 0.8969 | 0.9020 | 0.9881 | 0.9313 | 0.9465 | 0.8636 | 0.9115 | 0.9300 |
|  | 0.65 | 0.78 | 0.48 | 0.82 | 0.68 | 0.77 | 0.55 | 0.67 | 0.65 |
|  | 0.92 | 0.92 | 0.96 | 0.97 | 0.98 | 0.95 | 0.92 | 0.96 | 0.92 |
|  | 0.60 | 0.60 | 0.66 | 0.64 | 0.89 | 0.71 | 0.43 | 0.70 | 0.60 |
|  | 0.96 | 0.96 | 0.92 | 0.99 | 0.94 | 0.96 | 0.95 | 0.96 | 0.96 |
|  | 0.71 | 0.60 | 0.66 | 0.64 | 0.89 | 0.71 | 0.43 | 0.70 | 0.71 |
|  | 0.68 | 0.68 | 0.55 | 0.72 | 0.77 | 0.74 | 0.48 | 0.68 | 0.68 |
| MLP | 0.9955 | 0.991 | 0.9986 | 1.0000 | 0.9998 | 0.9965 | 0.9867 | 0.9929 | 0.9955 |
|  | 0.91 | 0.88 | 0.94 | 1.00 | 0.95 | 0.88 | 0.79 | 0.82 | 0.91 |
|  | 0.98 | 0.97 | 0.99 | 1.00 | 1.00 | 0.98 | 0.97 | 0.99 | 0.98 |
|  | 0.88 | 0.81 | 0.96 | 1.00 | 0.98 | 0.89 | 0.74 | 0.91 | 0.88 |
|  | 0.99 | 0.98 | 0.99 | 1.00 | 0.99 | 0.98 | 0.98 | 0.98 | 0.99 |
|  | 0.88 | 0.81 | 0.96 | 1.00 | 0.98 | 0.89 | 0.74 | 0.91 | 0.88 |
|  | 0.89 | 0.84 | 0.95 | 1.00 | 0.97 | 0.88 | 0.76 | 0.86 | 0.89 |
| XGBoost | 0.9972 | 0.9921 | 0.9973 | 0.9994 | 0.9985 | 0.9999 | 0.9779 | 0.9944 | 0.9972 |
|  | 0.96 | 0.92 | 0.90 | 1.00 | 0.92 | 1.00 | 0.82 | 0.85 | 0.96 |
|  | 0.98 | 0.98 | 0.98 | 0.99 | 0.99 | 1.00 | 1.00 | 0.98 | 0.98 |
|  | 0.90 | 0.87 | 0.90 | 0.92 | 0.96 | 0.98 | 0.96 | 0.87 | 0.90 |
|  | 0.99 | 0.99 | 0.98 | 1.00 | 0.98 | 1.00 | 0.98 | 0.98 | 0.99 |
|  | 0.90 | 0.87 | 0.90 | 0.92 | 0.96 | 0.98 | 0.96 | 0.87 | 0.90 |
|  | 0.93 | 0.89 | 0.90 | 0.96 | 0.94 | 0.99 | 0.89 | 0.86 | 0.93 |
| LGBoost | 0.9978 | 0.9924 | 0.9959 | 0.9994 | 0.9964 | 0.9961 | 0.9665 | 0.9944 | 0.9978 |
|  | 0.89 | 0.90 | 0.92 | 1.00 | 0.90 | 0.90 | 0.82 | 0.90 | 0.89 |
|  | 0.98 | 0.97 | 0.98 | 0.99 | 0.99 | 0.99 | 0.99 | 0.98 | 0.98 |
|  | 0.89 | 0.85 | 0.90 | 0.92 | 0.96 | 0.96 | 0.87 | 0.83 | 0.89 |
|  | 0.98 | 0.98 | 0.99 | 1.00 | 0.98 | 0.98 | 0.98 | 0.99 | 0.98 |
|  | 0.89 | 0.85 | 0.90 | 0.92 | 0.96 | 0.96 | 0.87 | 0.83 | 0.89 |
|  | 0.89 | 0.87 | 0.91 | 0.96 | 0.93 | 0.92 | 0.84 | 0.86 | 0.89 |
| SVM | 0.9969 | 0.9985 | 0.9999 | 1.0000 | 0.9998 | 0.9999 | 0.998 | 0.9985 | 0.9969 |
|  | 0.93 | 0.96 | 1.00 | 1.00 | 0.98 | 0.98 | 0.91 | 0.90 | 0.93 |
|  | 0.99 | 0.99 | 1.00 | 1.00 | 1.00 | 1.00 | 0.99 | 0.99 | 0.99 |
|  | 0.93 | 0.94 | 0.98 | 0.96 | 0.98 | 1.00 | 0.91 | 0.95 | 0.93 |
|  | 0.99 | 0.99 | 1.00 | 1.00 | 1.00 | 1.00 | 0.99 | 0.99 | 0.99 |
|  | 0.93 | 0.94 | 0.98 | 0.96 | 0.98 | 1.00 | 0.91 | 0.95 | 0.93 |
|  | 0.93 | 0.95 | 0.99 | 0.98 | 0.98 | 0.99 | 0.91 | 0.92 | 0.93 |

Table S3: The differences in AUC among the 10 models were compared using Delong's nonparametric method.

| Model1 | AUC1 | Model2 | AUC2 | P-value |
| --- | --- | --- | --- | --- |
| RF | 0.9895 | AB | 0.8946 | <0.01 |
| RF | 0.9895 | DT | 0.8718 | <0.01 |
| RF | 0.9895 | GB | 0.993 | 0.37 |
| RF | 0.9895 | KNN | 0.9912 | 0.68 |
| RF | 0.9895 | SVM | 0.9991 | <0.01 |
| RF | 0.9895 | NB | 0.9227 | <0.01 |
| RF | 0.9895 | MLP | 0.9955 | 0.10 |
| RF | 0.9895 | XGB | 0.995 | 0.13 |
| RF | 0.9895 | LGB | 0.9931 | 0.35 |
| AB | 0.8946 | DT | 0.8718 | 0.09 |
| AB | 0.8946 | GB | 0.993 | <0.01 |
| AB | 0.8946 | KNN | 0.9912 | <0.01 |
| AB | 0.8946 | SVM | 0.9991 | <0.01 |
| AB | 0.8946 | NB | 0.9227 | 0.02 |
| AB | 0.8946 | MLP | 0.9955 | <0.01 |
| AB | 0.8946 | XGB | 0.995 | <0.01 |
| AB | 0.8946 | LGB | 0.9931 | <0.01 |
| DT | 0.8718 | GB | 0.993 | <0.01 |
| DT | 0.8718 | KNN | 0.9912 | <0.01 |
| DT | 0.8718 | SVM | 0.9991 | <0.01 |
| DT | 0.8718 | NB | 0.9227 | <0.01 |
| DT | 0.8718 | MLP | 0.9955 | <0.01 |
| DT | 0.8718 | XGB | 0.995 | <0.01 |
| DT | 0.8718 | LGB | 0.9931 | <0.01 |
| GB | 0.993 | KNN | 0.9912 | 0.63 |
| GB | 0.993 | SVM | 0.9991 | 0.02 |
| GB | 0.993 | NB | 0.9227 | <0.01 |
| GB | 0.993 | MLP | 0.9955 | 0.43 |
| GB | 0.993 | XGB | 0.995 | 0.54 |
| GB | 0.993 | LGB | 0.9931 | 0.98 |
| KNN | 0.9912 | SVM | 0.9991 | 0.01 |
| KNN | 0.9912 | NB | 0.9227 | <0.01 |
| KNN | 0.9912 | MLP | 0.9955 | 0.21 |
| KNN | 0.9912 | XGB | 0.995 | 0.27 |
| KNN | 0.9912 | LGB | 0.9931 | 0.61 |
| SVM | 0.9991 | NB | 0.9227 | <0.01 |
| SVM | 0.9991 | MLP | 0.9955 | 0.10 |
| SVM | 0.9991 | XGB | 0.995 | 0.07 |
| SVM | 0.9991 | LGB | 0.9931 | 0.02 |
| NB | 0.9227 | MLP | 0.9955 | <0.01 |
| NB | 0.9227 | XGB | 0.995 | <0.01 |
| NB | 0.9227 | LGB | 0.9931 | <0.01 |
| MLP | 0.9955 | XGB | 0.995 | 0.86 |
| MLP | 0.9955 | LGB | 0.9931 | 0.45 |
| XGB | 0.995 | LGB | 0.9931 | 0.55 |

Table S4: The performance of XGB, SVM, and MLP models with varying numbers of features for identifying *Salmonella* serotypes on the validation set.

|  | Number of features | AUC | Number of features | AUC | P-value |
| --- | --- | --- | --- | --- | --- |
| XGB | 162 | 0.995 | 1 | 0.7557 | <0.01 |
|  | 162 | 0.995 | 2 | 0.8308 | <0.01 |
|  | 162 | 0.995 | 3 | 0.8503 | <0.01 |
|  | 162 | 0.995 | 4 | 0.9117 | <0.01 |
|  | 162 | 0.995 | 5 | 0.9397 | <0.01 |
|  | 162 | 0.995 | 6 | 0.9533 | <0.01 |
|  | 162 | 0.995 | 7 | 0.9582 | <0.01 |
|  | 162 | 0.995 | 8 | 0.9663 | <0.01 |
|  | 162 | 0.995 | 9 | 0.9708 | <0.01 |
|  | 162 | 0.995 | 10 | 0.9736 | <0.01 |
|  | 162 | 0.995 | 11 | 0.9829 | 0.01 |
|  | 162 | 0.995 | 12 | 0.9817 | <0.01 |
|  | 162 | 0.995 | 13 | 0.9844 | 0.01 |
|  | 162 | 0.995 | 14 | 0.9858 | 0.02 |
|  | 162 | 0.995 | 15 | 0.9869 | 0.04 |
|  | 162 | 0.995 | 16 | 0.9898 | 0.15 |
|  | 162 | 0.995 | 17 | 0.9899 | 0.16 |
|  | 162 | 0.995 | 18 | 0.9913 | 0.28 |
|  | 162 | 0.995 | 19 | 0.9899 | 0.16 |
|  | 162 | 0.995 | 20 | 0.9911 | 0.26 |
| SVM | 162 | 0.9991 | 1 | 0.769 | <0.01 |
|  | 162 | 0.9991 | 2 | 0.8515 | <0.01 |
|  | 162 | 0.9991 | 3 | 0.9035 | <0.01 |
|  | 162 | 0.9991 | 4 | 0.9253 | <0.01 |
|  | 162 | 0.9991 | 5 | 0.9316 | <0.01 |
|  | 162 | 0.9991 | 6 | 0.952 | <0.01 |
|  | 162 | 0.9991 | 7 | 0.9506 | <0.01 |
|  | 162 | 0.9991 | 8 | 0.9639 | <0.01 |
|  | 162 | 0.9991 | 9 | 0.9783 | <0.01 |
|  | 162 | 0.9991 | 10 | 0.983 | <0.01 |
|  | 162 | 0.9991 | 11 | 0.9839 | <0.01 |
|  | 162 | 0.9991 | 12 | 0.9836 | <0.01 |
|  | 162 | 0.9991 | 13 | 0.9836 | <0.01 |
|  | 162 | 0.9991 | 14 | 0.9838 | <0.01 |
|  | 162 | 0.9991 | 15 | 0.9882 | <0.01 |
|  | 162 | 0.9991 | 16 | 0.9881 | <0.01 |
|  | 162 | 0.9991 | 17 | 0.9906 | <0.01 |
|  | 162 | 0.9991 | 18 | 0.9904 | <0.01 |
|  | 162 | 0.9991 | 19 | 0.9906 | <0.01 |
|  | 162 | 0.9991 | 20 | 0.9913 | 0.01 |
| MLP | 162 | 0.9955 | 1 | 0.7036 | <0.01 |
|  | 162 | 0.9955 | 2 | 0.7282 | <0.01 |
|  | 162 | 0.9955 | 3 | 0.7658 | <0.01 |
|  | 162 | 0.9955 | 4 | 0.7887 | <0.01 |
|  | 162 | 0.9955 | 5 | 0.84 | <0.01 |
|  | 162 | 0.9955 | 6 | 0.8432 | <0.01 |
|  | 162 | 0.9955 | 7 | 0.856 | <0.01 |
|  | 162 | 0.9955 | 8 | 0.967 | <0.01 |
|  | 162 | 0.9955 | 9 | 0.8793 | <0.01 |
|  | 162 | 0.9955 | 10 | 0.9024 | <0.01 |
|  | 162 | 0.9955 | 11 | 0.9074 | <0.01 |
|  | 162 | 0.9955 | 12 | 0.9157 | <0.01 |
|  | 162 | 0.9955 | 13 | 0.9246 | <0.01 |
|  | 162 | 0.9955 | 14 | 0.9257 | <0.01 |
|  | 162 | 0.9955 | 15 | 0.9285 | <0.01 |
|  | 162 | 0.9955 | 16 | 0.9284 | <0.01 |
|  | 162 | 0.9955 | 17 | 0.9347 | <0.01 |
|  | 162 | 0.9955 | 18 | 0.9355 | <0.01 |
|  | 162 | 0.9955 | 19 | 0.9397 | <0.01 |
|  | 162 | 0.9955 | 20 | 0.9404 | <0.01 |

Table S5: The performance of the XGB model (16 features) in identifying *Salmonella* serotypes on the training set, internal validation set, and external validation set.

| XGB | Evaluation indexes | S. Group B | S. Group C1 | S. Group C2/3 | *Salmonella* Enteritidis | S. Group D | S. Group E1 | Non-A-F group | *Salmonella* Typhimurium |
| --- | --- | --- | --- | --- | --- | --- | --- | --- | --- |
| Train set  (16 features) | AUC | 0.9951 | 0.9779 | 0.982 | 0.9997 | 0.9948 | 0.9894 | 0.9808 | 0.993 |
|  | Sensitivity | 0.93 | 0.82 | 0.85 | 0.91 | 0.88 | 0.90 | 0.85 | 0.90 |
|  | specificity | 0.99 | 0.96 | 0.97 | 1.00 | 0.99 | 0.98 | 0.97 | 0.99 |
|  | PPV | 0.93 | 0.78 | 0.84 | 1.00 | 0.96 | 0.88 | 0.78 | 0.90 |
|  | NPV | 0.99 | 0.97 | 0.98 | 0.99 | 0.98 | 0.98 | 0.98 | 0.99 |
|  | accuracy | 0.93 | 0.78 | 0.84 | 1.00 | 0.96 | 0.88 | 0.78 | 0.90 |
|  | F1 score | 0.93 | 0.80 | 0.85 | 0.95 | 0.92 | 0.89 | 0.81 | 0.90 |
| Internal validation set  (16 features) | AUC | 0.9584 | 0.9181 | 0.9714 | 0.9838 | 0.961 | 0.9806 | 0.9738 | 0.9763 |
|  | Sensitivity | 0.88 | 0.68 | 0.78 | 0.85 | 0.83 | 0.88 | 0.76 | 0.75 |
|  | specificity | 0.92 | 0.99 | 0.97 | 1.00 | 0.98 | 0.96 | 0.98 | 0.98 |
|  | PPV | 0.62 | 0.88 | 0.77 | 0.94 | 0.85 | 0.80 | 0.84 | 0.88 |
|  | NPV | 0.98 | 0.96 | 0.97 | 1.00 | 0.98 | 0.98 | 0.96 | 0.95 |
|  | accuracy | 0.91 | 0.95 | 0.94 | 0.99 | 0.96 | 0.95 | 0.95 | 0.94 |
|  | F1 score | 0.73 | 0.77 | 0.78 | 0.89 | 0.96 | 0.84 | 0.80 | 0.81 |
| External validation set  (16 features) | AUC | 0.977 | 0.9281 | 0.9688 | 1 | 0.9917 | 0.9785 | 0.9832 | 0.9868 |
|  | Sensitivity | 0.76 | 0.62 | 0.70 | 1.00 | 0.80 | 0.78 | 0.83 | 0.83 |
|  | specificity | 0.94 | 0.97 | 0.96 | 1.00 | 0.99 | 0.95 | 0.96 | 0.96 |
|  | PPV | 0.69 | 0.77 | 0.81 | 0.90 | 0.91 | 0.71 | 0.60 | 0.75 |
|  | NPV | 0.96 | 0.94 | 0.93 | 1.00 | 0.97 | 0.97 | 0.99 | 0.97 |
|  | accuracy | 0.91 | 0.92 | 0.91 | 1.00 | 0.96 | 0.93 | 0.95 | 0.94 |
|  | F1 score | 0.72 | 0.69 | 0.75 | 0.95 | 0.85 | 0.74 | 0.70 | 0.79 |

Table S6: The performance of the MLP model (16 features) in identifying *Salmonella* serotypes on the training set, internal validation set, and external validation set.

| MLP | Evaluation indexes | S. Group B | S. Group C1 | S. Group C2/3 | *Salmonella* Enteritidis | S. Group D | S. Group E1 | Non-A-F group | *Salmonella* Typhimurium |
| --- | --- | --- | --- | --- | --- | --- | --- | --- | --- |
| Train set  (16 features) | AUC | 0.9081 | 0.8851 | 0.9063 | 0.996 | 0.9475 | 0.9051 | 0.9155 | 0.9591 |
|  | Sensitivity | 0.59 | 0.49 | 0.56 | 0.91 | 0.85 | 0.73 | 0.61 | 0.74 |
|  | specificity | 0.94 | 0.95 | 0.94 | 0.99 | 0.98 | 0.92 | 0.96 | 0.95 |
|  | PPV | 0.61 | 0.63 | 0.59 | 0.87 | 0.89 | 0.58 | 0.59 | 0.67 |
|  | NPV | 0.94 | 0.92 | 0.93 | 0.99 | 0.97 | 0.95 | 0.96 | 0.97 |
|  | accuracy | 0.61 | 0.63 | 0.59 | 0.87 | 0.89 | 0.58 | 0.59 | 0.67 |
|  | F1 score | 0.60 | 0.55 | 0.57 | 0.89 | 0.87 | 0.65 | 0.60 | 0.71 |
| Internal validation set  (16 features) | AUC | 0.8801 | 0.8416 | 0.7728 | 0.9239 | 0.9538 | 0.8697 | 0.8479 | 0.9231 |
|  | Sensitivity | 0.76 | 0.33 | 0.4 | 0.8 | 0.72 | 0.56 | 0.41 | 0.27 |
|  | specificity | 0.79 | 0.95 | 0.89 | 0.99 | 0.97 | 0.92 | 0.94 | 0.97 |
|  | PPV | 0.36 | 0.48 | 0.35 | 0.76 | 0.75 | 0.57 | 0.5 | 0.63 |
|  | NPV | 0.95 | 0.91 | 0.91 | 0.99 | 0.96 | 0.92 | 0.91 | 0.86 |
|  | accuracy | 0.36 | 0.48 | 0.35 | 0.76 | 0.75 | 0.57 | 0.5 | 0.62 |
|  | F1 score | 0.48 | 0.39 | 0.38 | 0.78 | 0.73 | 0.57 | 0.45 | 0.38 |
| External validation set  (16 features) | AUC | 0.9442 | 0.8745 | 0.9067 | 0.9966 | 0.9624 | 0.982 | 0.9292 | 0.9758 |
|  | Sensitivity | 0.73 | 0.59 | 0.5 | 0.78 | 0.75 | 0.92 | 0.72 | 0.78 |
|  | specificity | 0.72 | 0.93 | 0.97 | 1 | 0.98 | 0.95 | 0.95 | 0.96 |
|  | PPV | 0.61 | 0.58 | 0.81 | 0.88 | 0.88 | 0.76 | 0.5 | 0.76 |
|  | NPV | 0.95 | 0.93 | 0.9 | 0.99 | 0.96 | 0.99 | 0.98 | 0.97 |
|  | accuracy | 0.61 | 0.57 | 0.81 | 0.88 | 0.88 | 0.76 | 0.5 | 0.76 |
|  | F1 score | 0.67 | 0.58 | 0.62 | 0.82 | 0.81 | 0.83 | 0.59 | 0.77 |

Table S7: The performance of the SVM model (16 features) in identifying *Salmonella* serotypes on the training set, internal validation set, and external validation set.

| SVM | Evaluation indexes | S. Group B | S. Group C1 | S. Group C2/3 | *Salmonella* Enteritidis | S. Group D | S. Group E1 | Non-A-F group | *Salmonella* Typhimurium |
| --- | --- | --- | --- | --- | --- | --- | --- | --- | --- |
| Train set  (16 features) | AUC | 0.9838 | 0.9775 | 0.9878 | 0.9984 | 0.9937 | 0.9987 | 0.9793 | 0.9794 |
|  | Sensitivity | 0.87 | 0.84 | 0.92 | 1.00 | 0.88 | 0.96 | 0.73 | 0.79 |
|  | specificity | 0.96 | 0.97 | 0.98 | 0.99 | 0.99 | 0.99 | 0.99 | 0.98 |
|  | PPV | 0.77 | 0.84 | 0.88 | 0.85 | 0.95 | 0.94 | 0.92 | 0.84 |
|  | NPV | 0.98 | 0.97 | 0.99 | 1.00 | 0.98 | 0.99 | 0.07 | 0.98 |
|  | accuracy | 0.77 | 0.84 | 0.88 | 0.85 | 0.95 | 0.94 | 0.92 | 0.84 |
|  | F1 score | 0.82 | 0.84 | 0.90 | 0.92 | 0.91 | 0.95 | 0.81 | 0.82 |
| Internal validation set  (16 features) | AUC | 0.9211 | 0.9075 | 0.8944 | 0.9605 | 0.9874 | 0.9455 | 0.9498 | 0..9466 |
|  | Sensitivity | 0.88 | 0.53 | 0.64 | 0.75 | 0.85 | 0.82 | 0.46 | 0.30 |
|  | specificity | 0.81 | 0.96 | 0.91 | 1.00 | 0.98 | 0.97 | 0.97 | 0.98 |
|  | PPV | 0.42 | 0.63 | 0.52 | 0.83 | 0.83 | 0.84 | 0.74 | 0.77 |
|  | NPV | 0.98 | 0.94 | 0.94 | 0.99 | 0.98 | 0.97 | 0.92 | 0.87 |
|  | accuracy | 0.82 | 0.90 | 0.88 | 0.99 | 0.96 | 0.95 | 0.91 | 0.86 |
|  | F1 score | 0.57 | 0.57 | 0.57 | 0.79 | 0.84 | 0.83 | 0.57 | 0.30 |
| External validation set  (16 features) | AUC | 0.9736 | 0.9002 | 0.955 | 0.9987 | 0.9945 | 0.992 | 0.9731 | 0.99 |
|  | Sensitivity | 0.76 | 0.67 | 0.84 | 0.89 | 0.85 | 1.00 | 0.78 | 0.86 |
|  | specificity | 0.95 | 0.94 | 0.97 | 1.00 | 0.99 | 0.97 | 1.00 | 0.98 |
|  | PPV | 0.74 | 0.63 | 0.88 | 0.89 | 0.94 | 0.82 | 1.00 | 0.89 |
|  | NPV | 0.96 | 0.94 | 0.96 | 1.00 | 0.97 | 1.00 | 0.98 | 0.98 |
|  | accuracy | 0.92 | 0.90 | 0.95 | 0.99 | 0.97 | 0.97 | 0.99 | 0.97 |
|  | F1 score | 0.75 | 0.65 | 0.86 | 0.89 | 0.89 | 0.90 | 0.88 | 0.87 |

Figure S1：PCA(A) and t-SNE(B) were used for the feasibility study of *Salmonella* serotype classification based on mass spectrometry data.


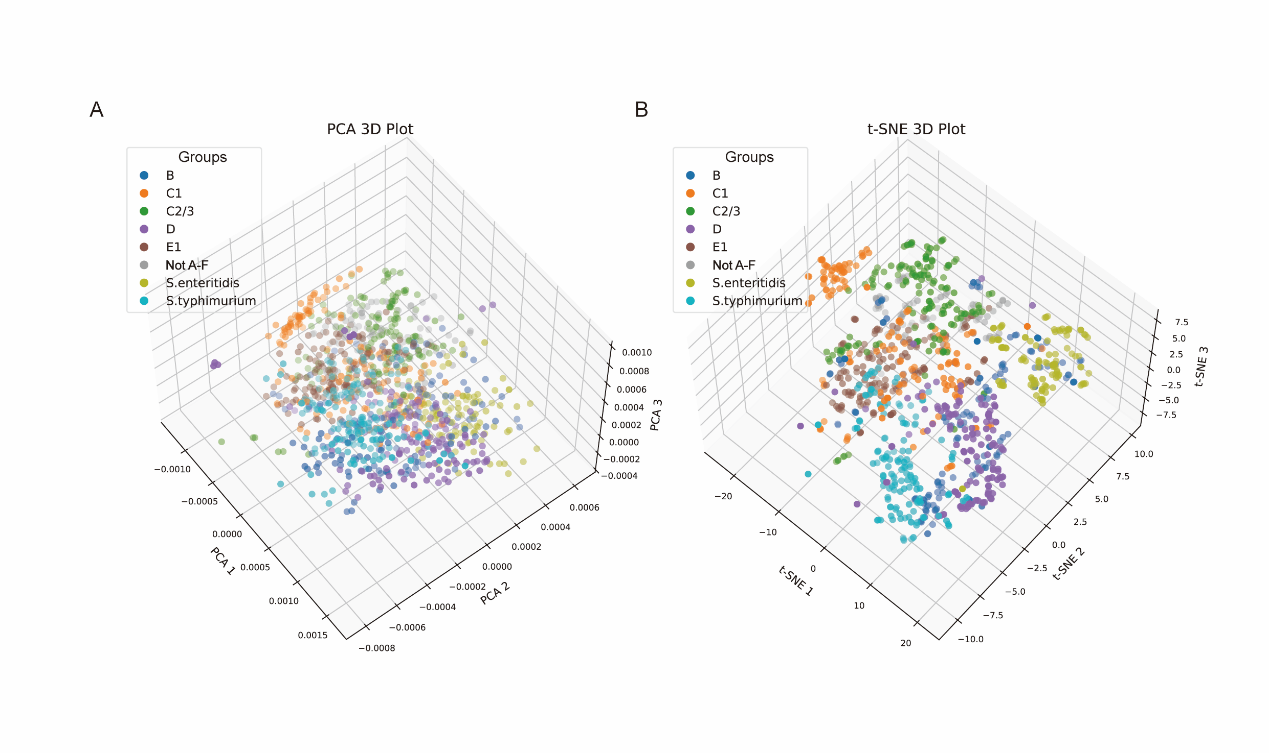


Figure S2: PR curves for identifying *Salmonella* serotypes using 10 machine learning models.


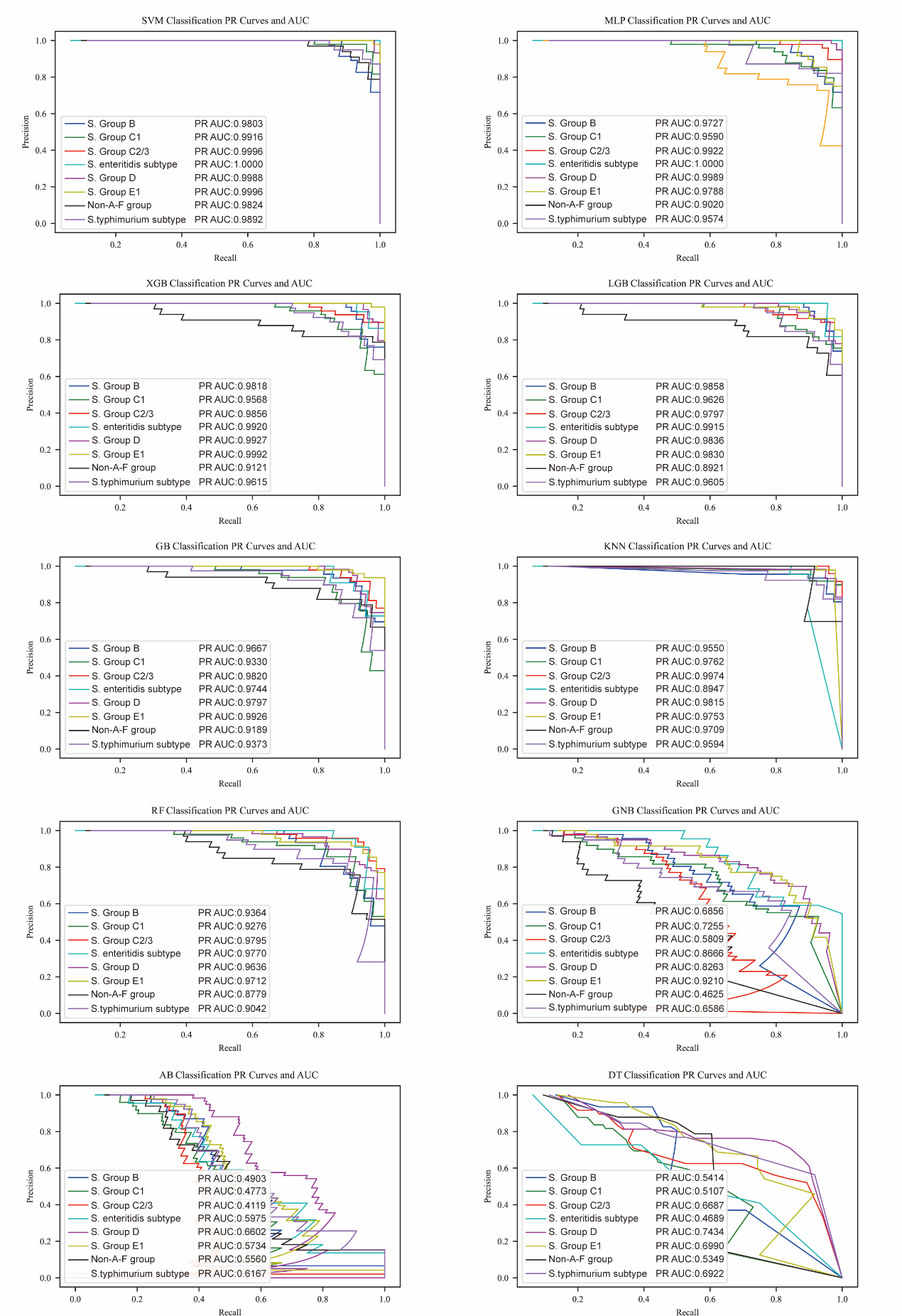


Figure S3: Confusion matrix heat maps for identifying *Salmonella* serotypes using 10 machine learning models.


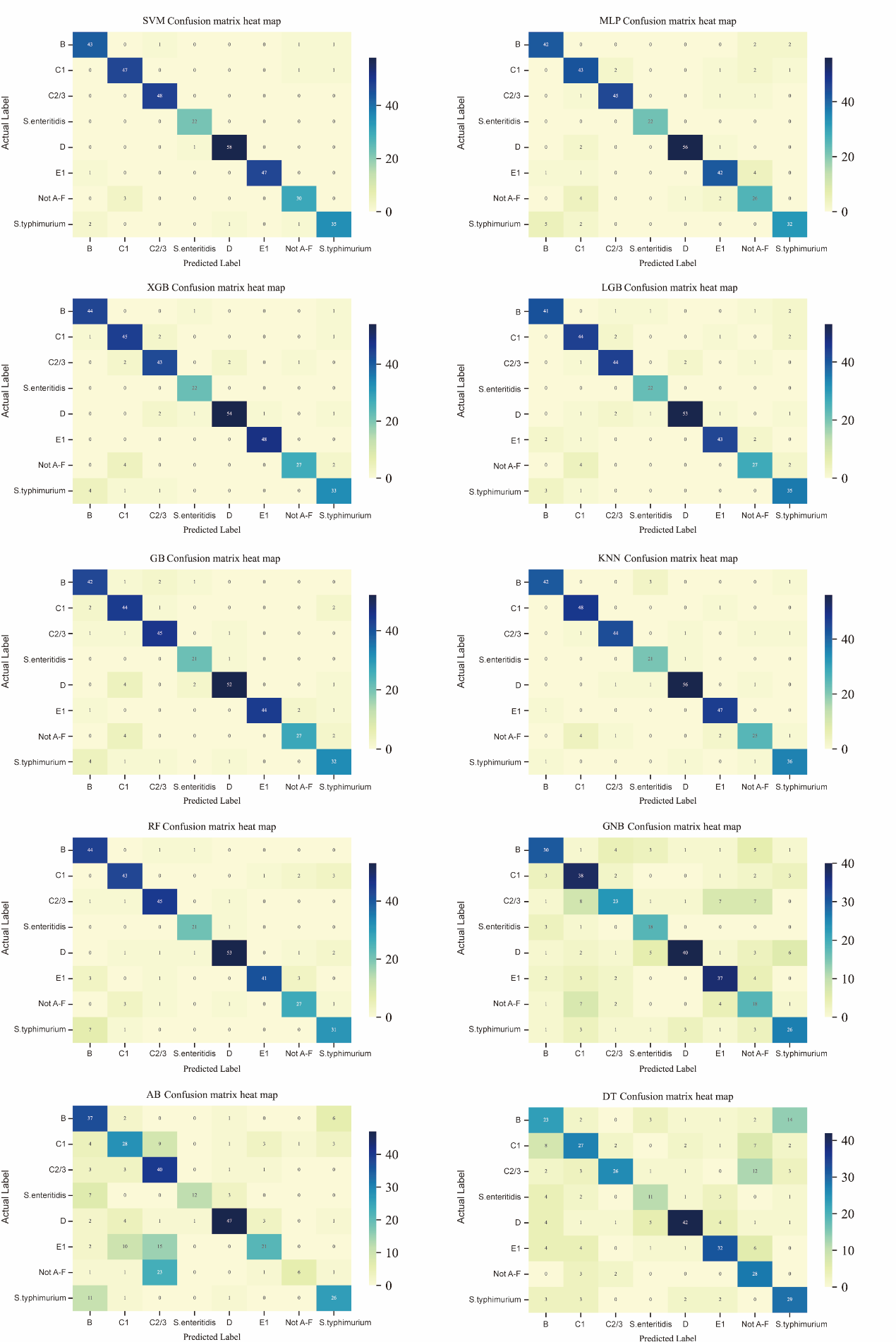


Figure S4: The PR curves of the final XGB model with 16 features on the training set.


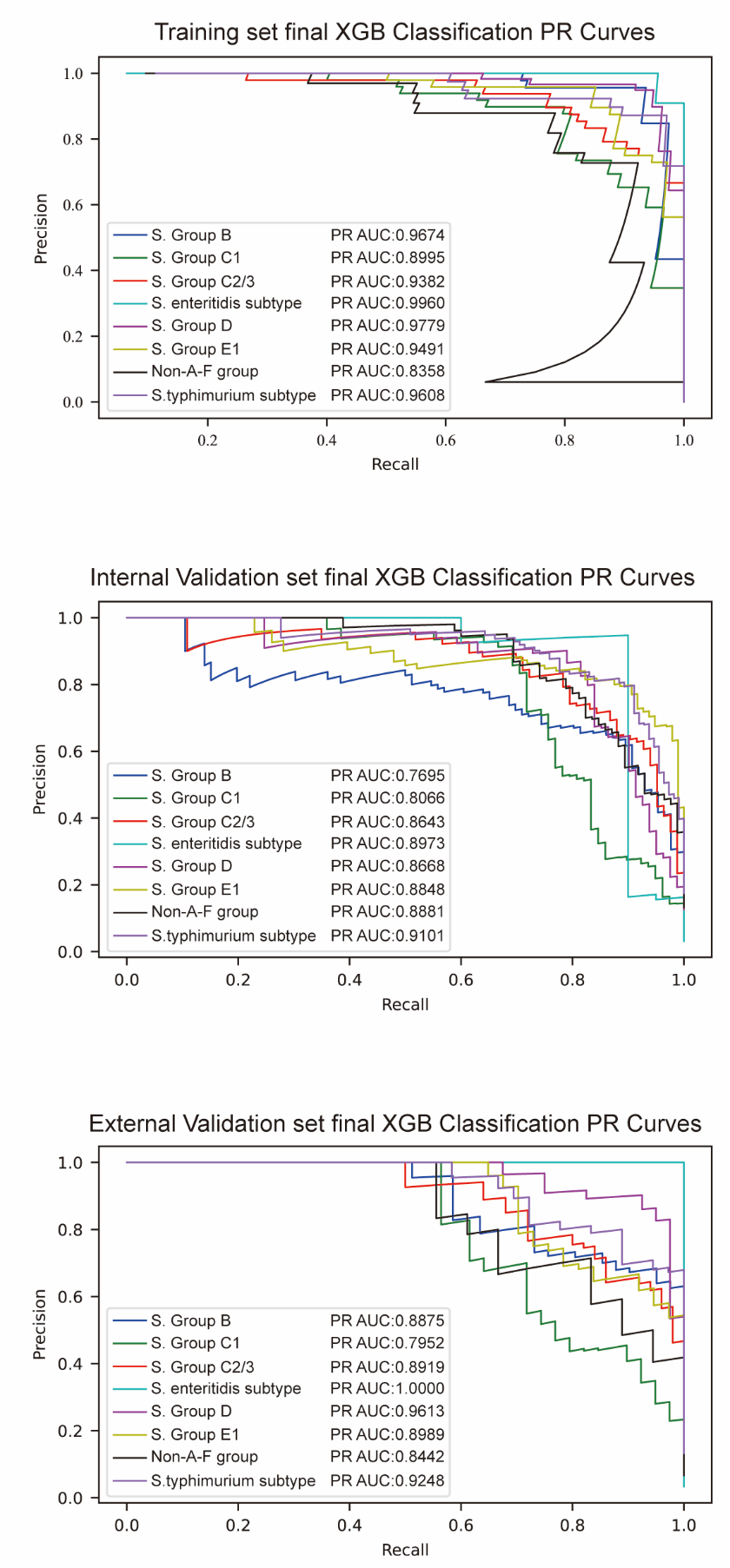


Figure S5: The ROC curves, PR curves and confusion matrix heat maps of the MLP model with 16 features on the training set, internal validation set, and external validation set.


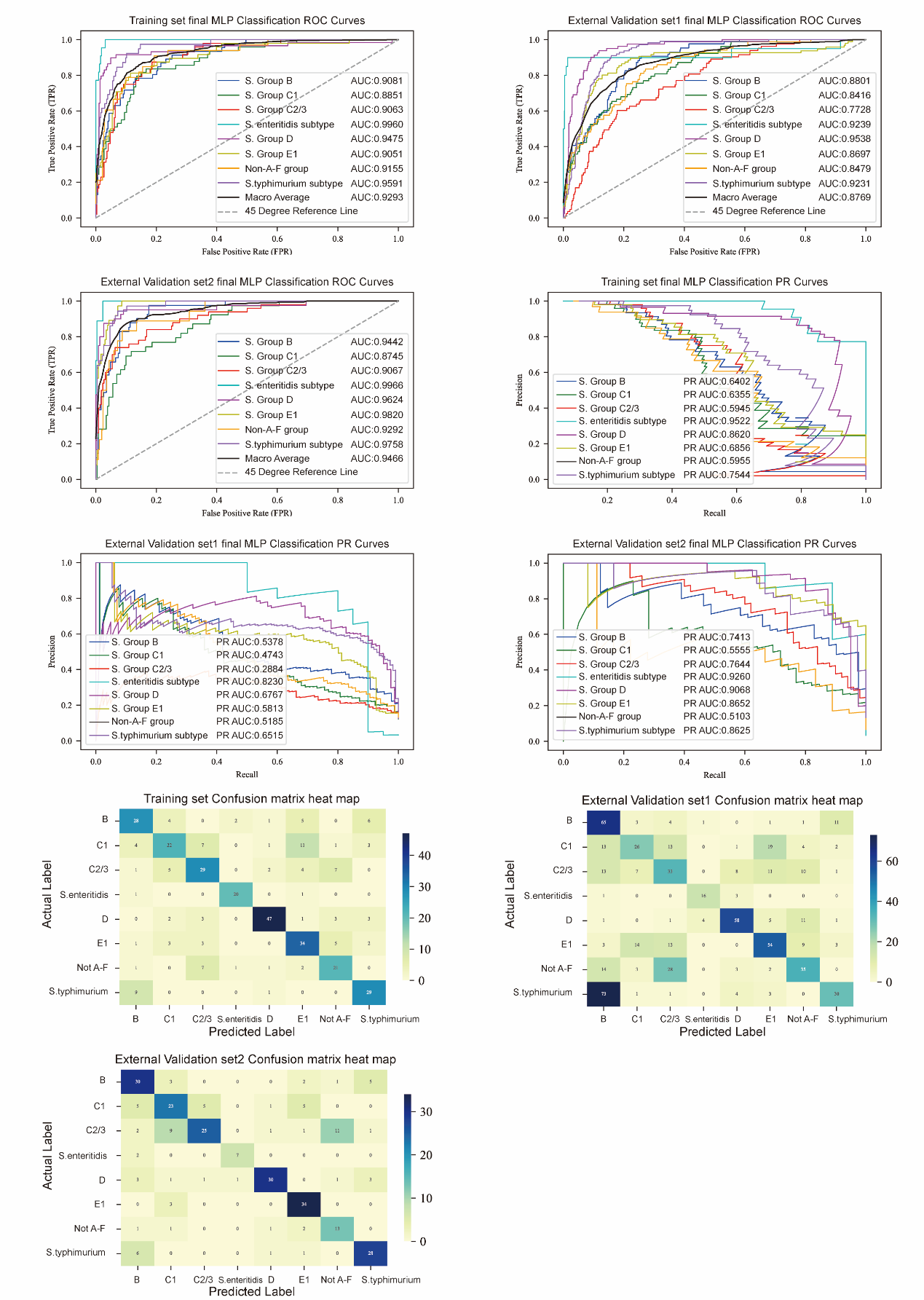


Figure S6: The ROC curves, PR curves and confusion matrix heat maps of the SVM model with 16 features on the training set, internal validation set, and external validation set.


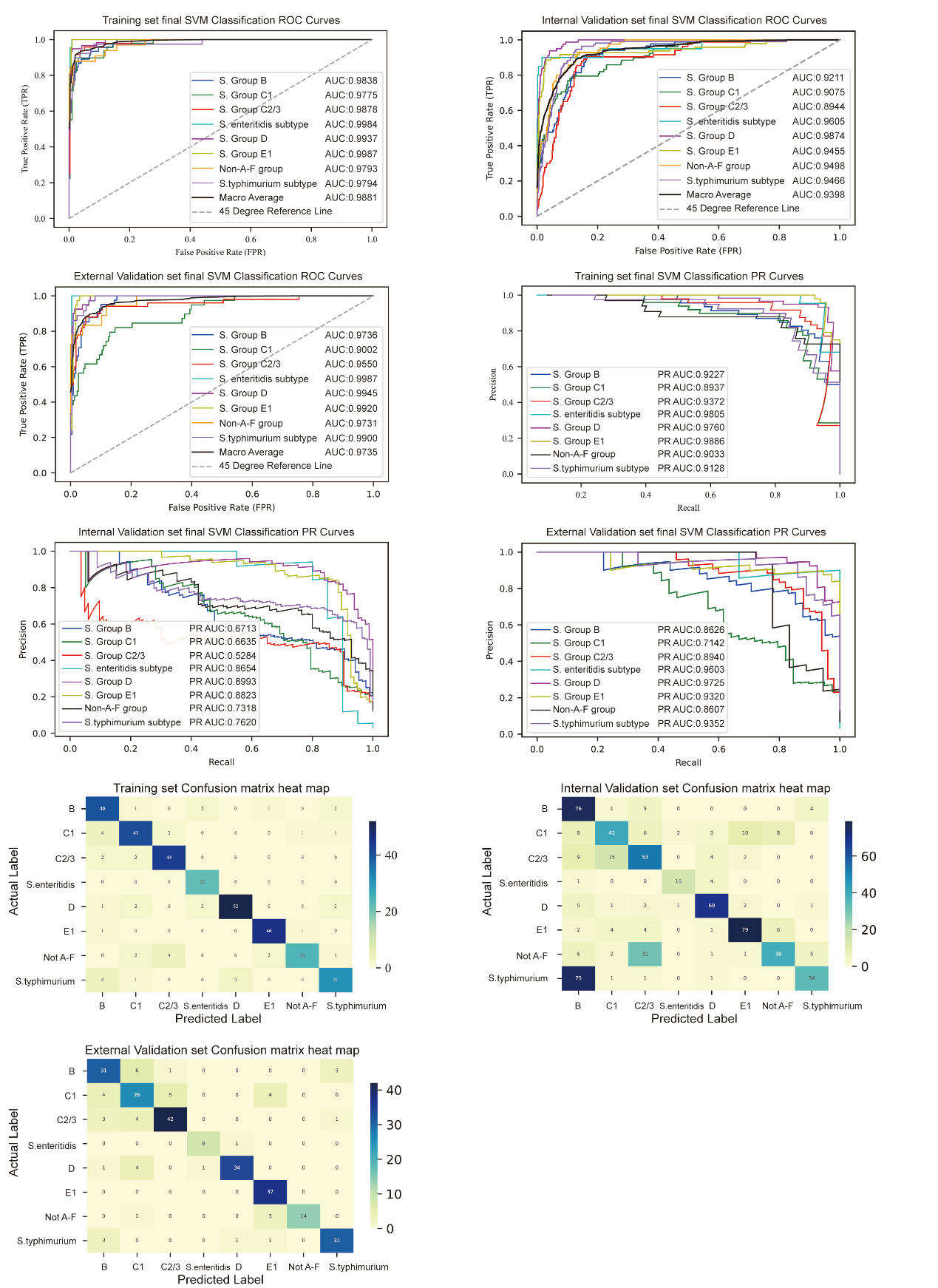


Figure S7: Description of features in the training set. The violin plot showed the overall distribution of 16 features across 8 serotypes in the training set (A). The volcano plot was used to display statistically significant upregulated and downregulated features in each serotype (B).


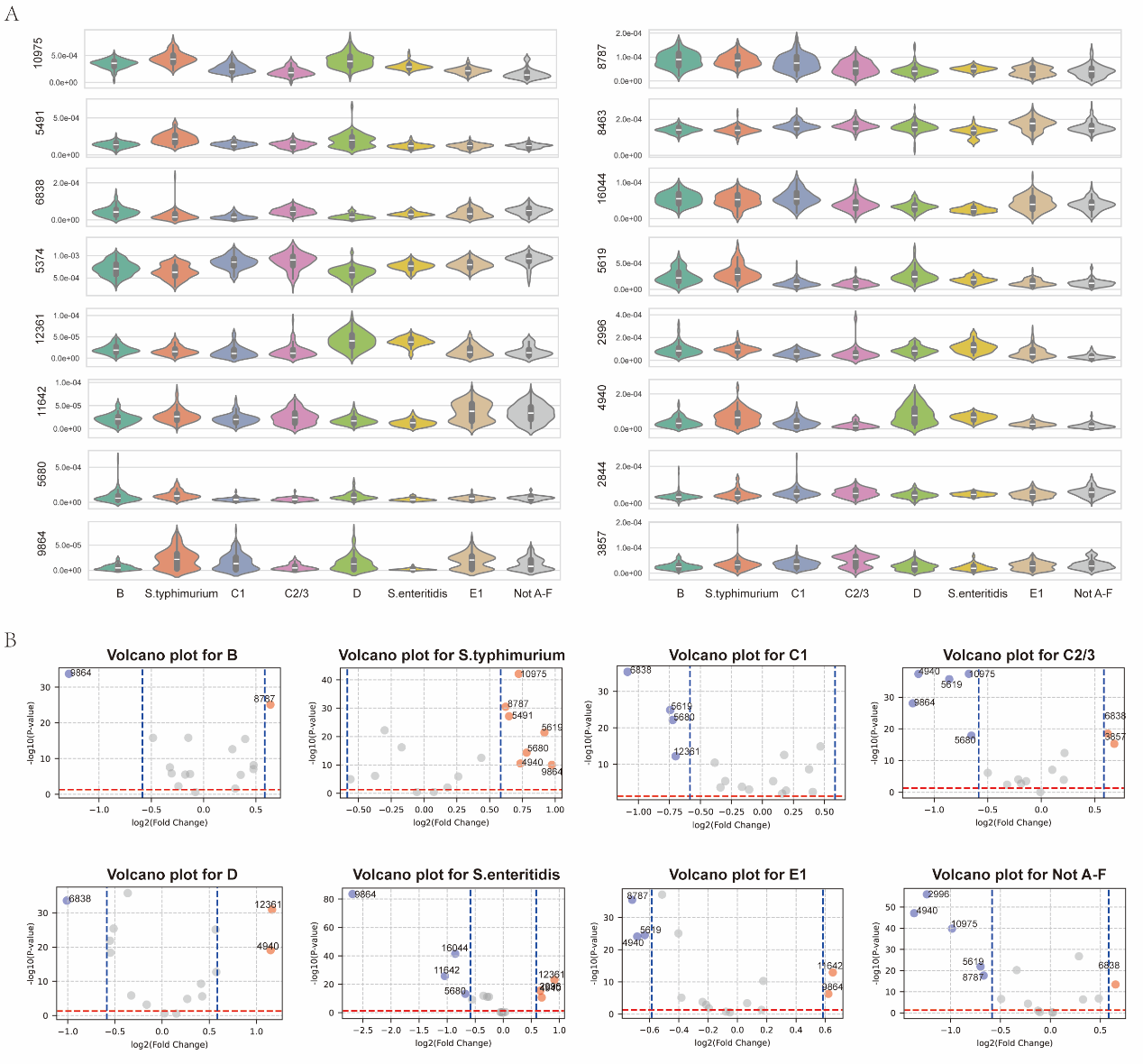


Figure S8: Description of features in the internal validation set. Heatmap of the intensities of 16 characteristic peaks across different *Salmonella* serotypes in the internal validation set (A). Bubble chart of the correlations among the 16 features in the internal validation set (B). The violin plot showed the overall distribution of 16 features across 8 serotypes in the internal validation set (C). The volcano plot was used to display statistically significant upregulated and downregulated features in each serotype (D).


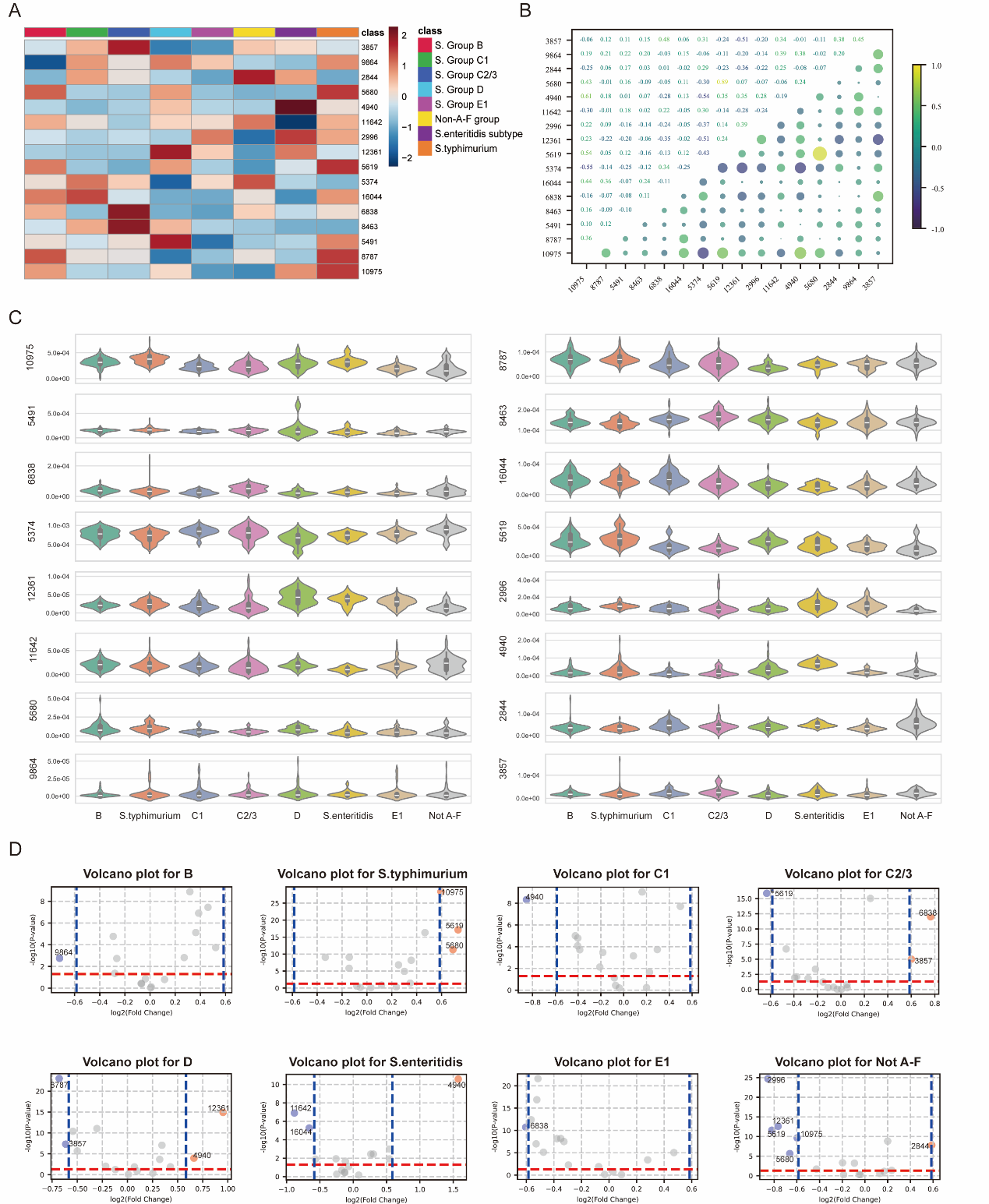


Figure S9: Description of features in the external validation set. Heatmap of the intensities of 16 characteristic peaks across different *Salmonella* serotypes in the external validation set (A). Bubble chart of the correlations among the 16 features in the external validation set (B). The violin plot showed the overall distribution of 16 features across 8 serotypes in the external validation set (C). The volcano plot was used to display statistically significant upregulated and downregulated features in each serotype (D).


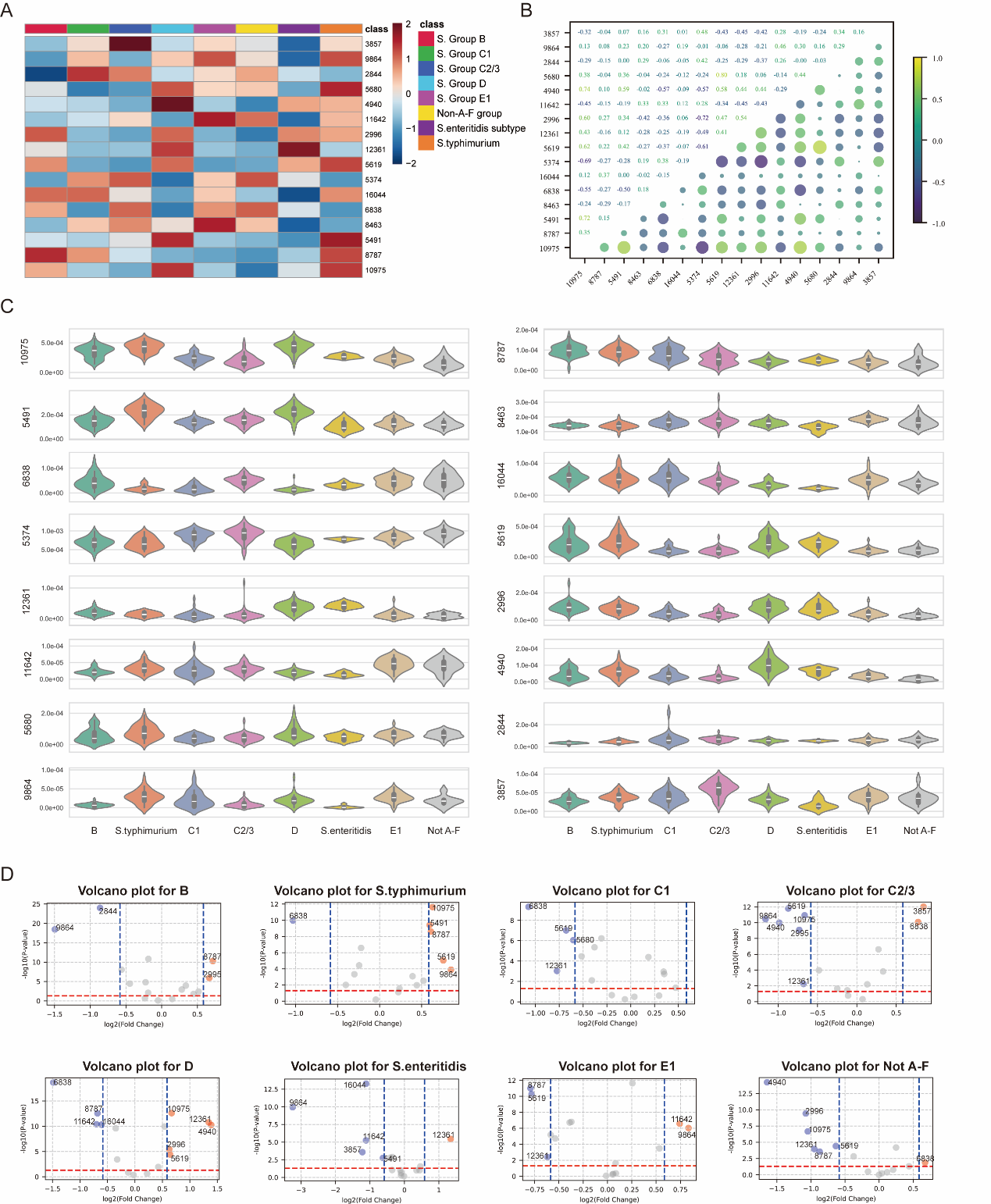


Figure S10: The DCA of the final XGB model (16 features) in the training set, showing the identification performance for 8 *Salmonella* serotypes.


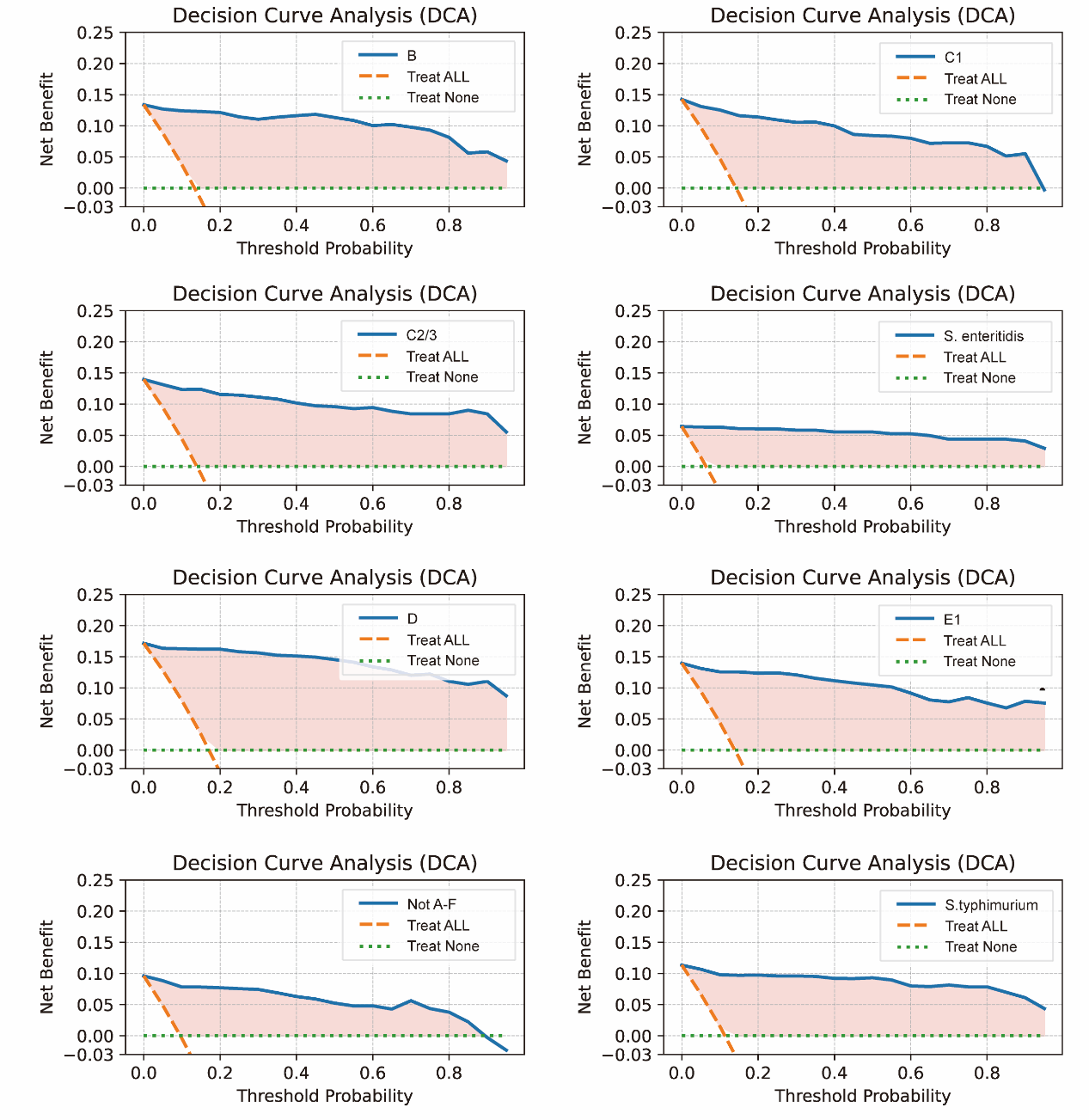


Figure S11: The DCA of the final XGB model (16 features) in the internal validation set, showing the identification performance for 8 *Salmonella* serotypes.


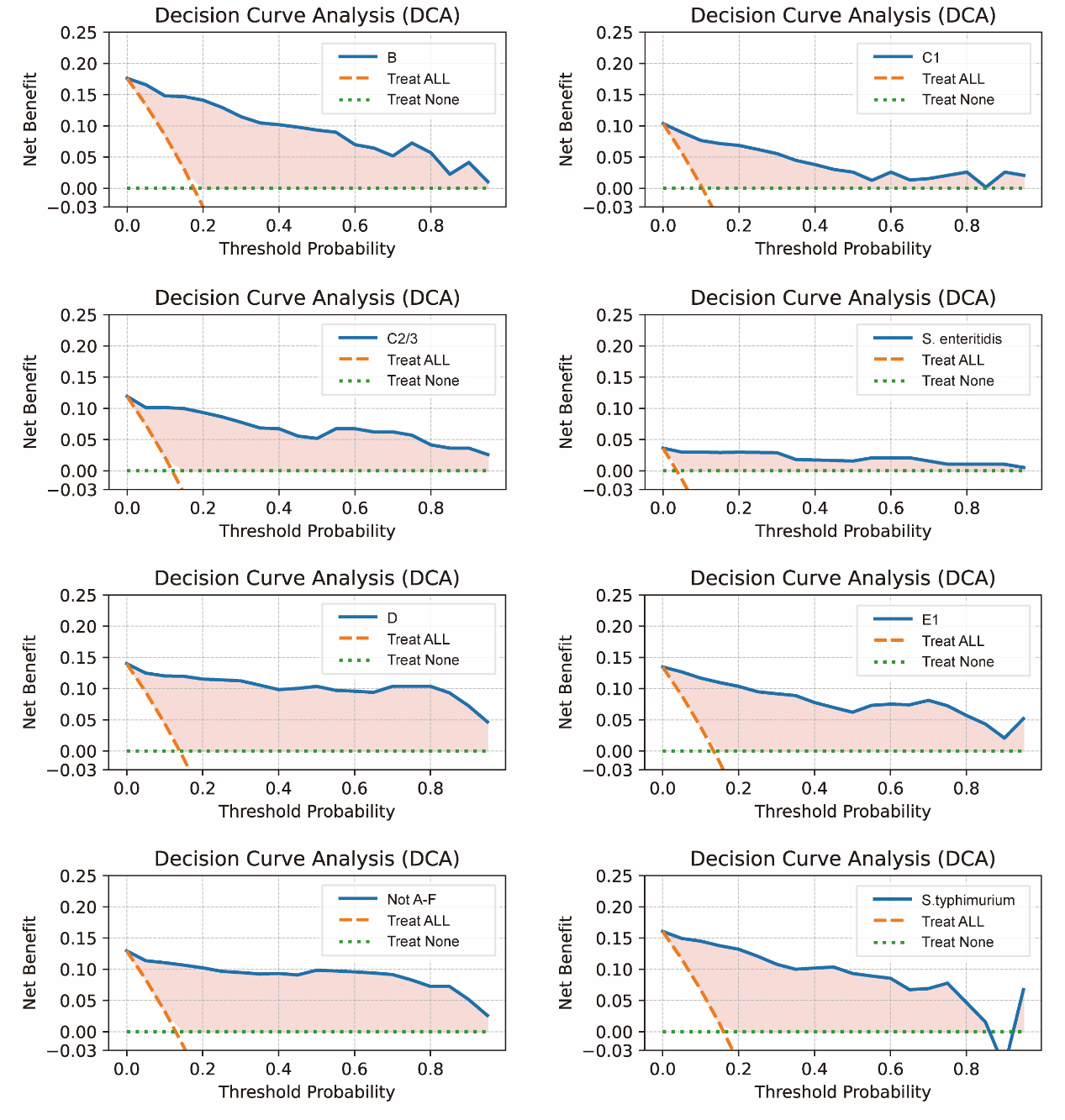


Figure S12: The DCA of the final XGB model (16 features) in the external validation set, showing the identification performance for 8 *Salmonella* serotypes.


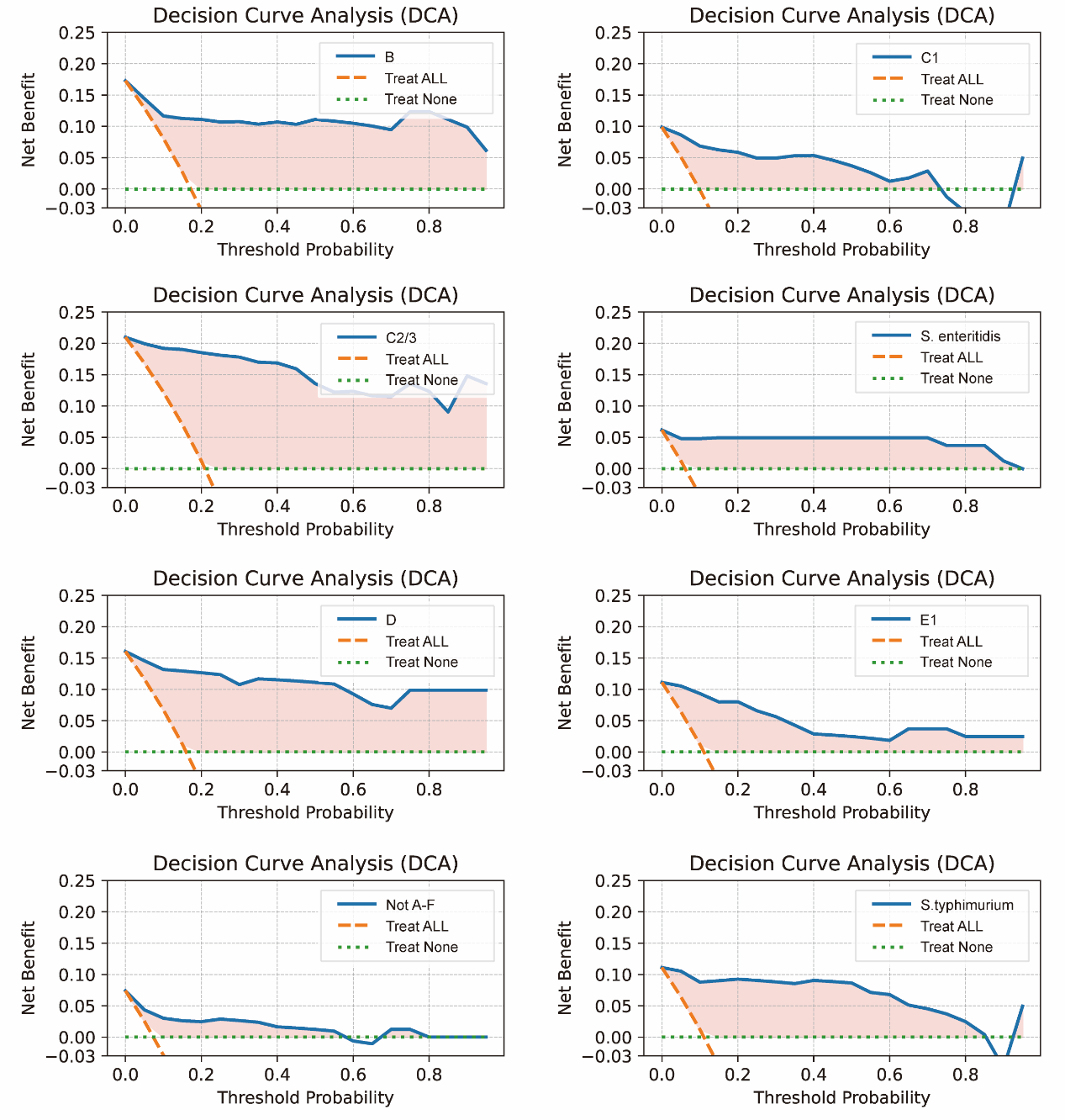


20. Code

(1) Data processing of MALDI-TOF MS protein spectra

library('MALDIquant')

library('MALDIquantForeign')

library('readxl')

samples = read_excel('')

spectra = importTxt(getwd())

spectra = transformIntensity(spectra, method = "sqrt")

spectra = smoothIntensity(spectra, method = "SavitzkyGolay", halfWindowSize = #)

spectra = removeBaseline(spectra, method = "SNIP", iterations = #)

spectra = calibrateIntensity(spectra, method = "TIC")

avgSpectra = averageMassSpectra(

spectra,

labels = samples$group[match(

samples$file,

sapply(spectra, function(s) {

tail(strsplit(s@metaData$file, '[\\/]')[[1]], 1)

})

)]

)

avgSpectra = alignSpectra(

avgSpectra,

halfWindowSize = #, SNR = #,

tolerance = #, warpingMethod = "lowess"

)

peaks = detectPeaks(avgSpectra, method = "MAD", halfWindowSize = #, SNR = #)

peaks = binPeaks(peaks, tolerance = #)

intensity = intensityMatrix(peaks, avgSpectra)

intensity = t(intensity)

colnames(intensity) = samples$group[match(

sapply(peaks, function(s) {

tail(strsplit(s@metaData$file, '[\\/]')[[1]], 1)

}),

samples$file

)]

write.csv(intensity, '')

(2) Missing value imputation

import pandas as pd

file_path = ''

df = pd.read_csv(file_path)

missing_values = df.isnull().sum()

print("缺失值数量：")

print(missing_values)

numeric_cols = df.select_dtypes(include=['int64', 'float64']).columns

for col in numeric_cols:

median_value = df[col].median()

df[col].fillna(median_value, inplace=True)

print("\n填充后缺失值数量：")

print(df.isnull().sum())

output_file_path = ''

df.to_csv(output_file_path, index=False)

print(f"处理后的数据已保存至{output_file_path}")

(3)

**AB**

import numpy as np

import pandas as pd

import matplotlib.pyplot as plt

from sklearn.ensemble import AdaBoostClassifier

from sklearn.model_selection import train_test_split

from sklearn.metrics import classification_report, confusion_matrix, roc_curve, auc, precision_recall_curve, \

average_precision_score, roc_auc_score

from sklearn.preprocessing import MinMaxScaler, label_binarize

import seaborn as sns

plt.rcParams['font.family'] = 'Times New Roman'

plt.rcParams['font.size'] = 10

plt.rcParams['axes.unicode_minus'] = False

data = pd.read_csv('')

X = data.iloc[:, 1:]

y = data.iloc[:, 0]

xtrain, xtest, ytrain, ytest = train_test_split(X, y, test_size=0.3, random_state=42)

scaler = MinMaxScaler()

xtrain_s = scaler.fit_transform(xtrain)

xtest_s = scaler.transform(xtest)

model_rf = AdaBoostClassifier()

model_rf.fit(xtrain_s, ytrain)

y_pred = model_rf.predict(xtest_s)

y_pred_proba = model_rf.predict_proba(xtest_s)

print("Classification Report:")

print(classification_report(ytest, y_pred))

conf_matrix = confusion_matrix(ytest, y_pred)

plt.figure(figsize=(10, 5), dpi=300)

sns.heatmap(conf_matrix, annot=True, annot_kws={'size':4},

fmt='d', cmap='YlGnBu', cbar_kws={'shrink': 0.75})

plt.tick_params(axis='both', which='major', labelsize=7)

plt.xlabel('Predicted Label', fontsize=7)

plt.ylabel('True Label', fontsize=7)

plt.title('Confusion matrix heat map', fontsize=8)

plt.show()

num_classes = conf_matrix.shape[0]

for i in range(num_classes):

tp = conf_matrix[i, i] # True Positive for class i

fp = conf_matrix[:, i].sum() - tp # False Positive for class i

fn = conf_matrix[i, :].sum() - tp # False Negative for class i

tn = conf_matrix.sum() - (tp + fp + fn) # True Negative for class i

specificity = tn / (tn + fp) if (tn + fp) != 0 else 0

positive_predictive_value = tp / (tp + fp) if (tp + fp) != 0 else 0

negative_predictive_value = tn / (tn + fn) if (tn + fn) != 0 else 0

sensitivity = recall = tp / (tp + fn) if (tp + fn) != 0 else 0

print(f"Class {i + 1}:")

print(f"Specificity: {specificity:.4f}")

print(f"Positive Predictive Value: {positive_predictive_value:.4f}")

print(f"Negative Predictive Value: {negative_predictive_value:.4f}")

print(f"Sensitivity (Recall for class {i + 1}): {sensitivity:.4f}")

ytest_one_rf = label_binarize(ytest, classes=np.unique(y))

rf_AUC = {}

rf_FPR = {}

rf_TPR = {}

for i in range(ytest_one_rf.shape[1]):

rf_FPR[i], rf_TPR[i], _ = roc_curve(ytest_one_rf[:, i], y_pred_proba[:, i])

rf_AUC[i] = auc(rf_FPR[i], rf_TPR[i])

print("ROC AUC for each class:", rf_AUC)

rf_FPR_final = np.unique(np.concatenate([rf_FPR[i] for i in range(ytest_one_rf.shape[1])]))

rf_TPR_all = np.zeros_like(rf_FPR_final)

for i in range(ytest_one_rf.shape[1]):

rf_TPR_all += np.interp(rf_FPR_final, rf_FPR[i], rf_TPR[i])

rf_TPR_final = rf_TPR_all / ytest_one_rf.shape[1]

rf_AUC_final = auc(rf_FPR_final, rf_TPR_final)

print(f"Macro Average AUC with XGBoost: {rf_AUC_final}")

plt.figure(figsize=(10, 5), dpi=300)

plt.plot(rf_FPR[0], rf_TPR[0], color='b', linestyle='-', label='Class 1 ROC AUC={:.4f}'.format(rf_AUC[0]), lw=0.8)

plt.plot(rf_FPR[1], rf_TPR[1], color='g', linestyle='-', label='Class 2 ROC AUC={:.4f}'.format(rf_AUC[1]), lw=0.8)

plt.plot(rf_FPR[2], rf_TPR[2], color='r', linestyle='-', label='Class 3 ROC AUC={:.4f}'.format(rf_AUC[2]), lw=0.8)

plt.plot(rf_FPR[3], rf_TPR[3], color='c', linestyle='-', label='Class 4 ROC AUC={:.4f}'.format(rf_AUC[3]), lw=0.8)

plt.plot(rf_FPR[4], rf_TPR[4], color='m', linestyle='-', label='Class 5 ROC AUC={:.4f}'.format(rf_AUC[4]), lw=0.8)

plt.plot(rf_FPR[5], rf_TPR[5], color='y', linestyle='-', label='Class 6 ROC AUC={:.4f}'.format(rf_AUC[5]), lw=0.8)

plt.plot(rf_FPR[6], rf_TPR[6], color='#FFA500', linestyle='-', label='Class 7 ROC AUC={:.4f}'.format(rf_AUC[6]), lw=0.8)

plt.plot(rf_FPR[7], rf_TPR[7], color='#9467bd', linestyle='-', label='Class 8 ROC AUC={:.4f}'.format(rf_AUC[7]), lw=0.8)

# 宏平均ROC曲线

plt.plot(rf_FPR_final, rf_TPR_final, color='#000000', linestyle='-', label='Macro Average ROC AUC={:.4f}'.format(rf_AUC_final), lw=1)

plt.plot([0, 1], [0, 1], color='gray', linestyle='--', lw=1, label='45 Degree Reference Line')

plt.tick_params(axis='both', which='major', labelsize=7)

plt.xlabel('False Positive Rate (FPR)', fontsize=7)

plt.ylabel('True Positive Rate (TPR)', fontsize=7)

plt.title('AdaBoost Classification ROC Curves and AUC', fontsize=8)

plt.legend(loc='lower right', framealpha=0.9, fontsize=5)

plt.show()

pr_AUC = {}

pr_Precision = {}

pr_Recall = {}

for i in range(ytest_one_rf.shape[1]):

pr_Recall[i], pr_Precision[i], _ = precision_recall_curve(ytest_one_rf[:, i], y_pred_proba[:, i])

pr_AUC[i] = average_precision_score(ytest_one_rf[:, i], y_pred_proba[:, i])

print("PR AUC for each class:", pr_AUC)

plt.figure(figsize=(10, 5), dpi=300)

plt.plot(pr_Recall[0], pr_Precision[0], color='b', linestyle='-',label=f'Class1 PR AUC={pr_AUC[0]:.4f}', lw=0.8)

plt.plot(pr_Recall[1], pr_Precision[1], color='g', linestyle='-',label=f'Class2 PR AUC={pr_AUC[1]:.4f}', lw=0.8)

plt.plot(pr_Recall[2], pr_Precision[2], color='r', linestyle='-',label=f'Class3 PR AUC={pr_AUC[2]:.4f}', lw=0.8)

plt.plot(pr_Recall[3], pr_Precision[3], color='c', linestyle='-',label=f'Class4 PR AUC={pr_AUC[3]:.4f}', lw=0.8)

plt.plot(pr_Recall[4], pr_Precision[4], color='m', linestyle='-',label=f'Class5 PR AUC={pr_AUC[4]:.4f}', lw=0.8)

plt.plot(pr_Recall[5], pr_Precision[5], color='y', linestyle='-',label=f'Class6 PR AUC={pr_AUC[5]:.4f}', lw=0.8)

plt.plot(pr_Recall[6], pr_Precision[6], color='k', linestyle='-',label=f'Class7 PR AUC={pr_AUC[6]:.4f}', lw=0.8)

plt.plot(pr_Recall[7], pr_Precision[7], color='#9467bd', linestyle='-',label=f'Class8 PR AUC={pr_AUC[7]:.4f}', lw=0.8)

plt.tick_params(axis='both', which='major', labelsize=7)

plt.xlabel('Recall', fontsize=7)

plt.ylabel('Precision', fontsize=7)

plt.title('AdaBoost Classification PR Curves and AUC', fontsize=8)

plt.legend(loc='lower left', framealpha=0.9, fontsize=5)

plt.show()

**MLP**

import numpy as np

import pandas as pd

import matplotlib.pyplot as plt

from sklearn.model_selection import train_test_split

from sklearn.metrics import classification_report, confusion_matrix, roc_curve, auc, precision_recall_curve, \

average_precision_score

from sklearn.neural_network import MLPClassifier

from sklearn.preprocessing import MinMaxScaler, label_binarize

import seaborn as sns

plt.rcParams['font.family'] = 'Times New Roman'

plt.rcParams['font.size'] = 10

plt.rcParams['axes.unicode_minus'] = False

data = pd.read_csv('')

X = data.iloc[:, 1:]

y = data.iloc[:, 0]

xtrain, xtest, ytrain, ytest = train_test_split(X, y, test_size=0.3, random_state=42)

scaler = MinMaxScaler()

xtrain_s = scaler.fit_transform(xtrain)

xtest_s = scaler.transform(xtest)

mlp_model = MLPClassifier()

mlp_model.fit(xtrain_s, ytrain)

y_pred = mlp_model.predict(xtest_s)

y_pred_proba = mlp_model.predict_proba(xtest_s)

print("Classification Report:")

print(classification_report(ytest, y_pred))

conf_matrix = confusion_matrix(ytest, y_pred)

plt.figure(figsize=(10, 5), dpi=300)

sns.heatmap(conf_matrix, annot=True, annot_kws={'size':4},

fmt='d', cmap='YlGnBu', cbar_kws={'shrink': 0.75})

plt.tick_params(axis='both', which='major', labelsize=7)

plt.xlabel('Predicted Label', fontsize=7)

plt.ylabel('True Label', fontsize=7)

plt.title('Confusion matrix heat map', fontsize=8)

plt.show()

num_classes = conf_matrix.shape[0]

for i in range(num_classes):

tp = conf_matrix[i, i] # True Positive for class i

fp = conf_matrix[:, i].sum() - tp # False Positive for class i

fn = conf_matrix[i, :].sum() - tp # False Negative for class i

tn = conf_matrix.sum() - (tp + fp + fn) # True Negative for class i

specificity = tn / (tn + fp) if (tn + fp) != 0 else 0

positive_predictive_value = tp / (tp + fp) if (tp + fp) != 0 else 0

negative_predictive_value = tn / (tn + fn) if (tn + fn) != 0 else 0

sensitivity = recall = tp / (tp + fn) if (tp + fn) != 0 else 0

print(f"Class {i + 1}:")

print(f"Specificity: {specificity:.4f}")

print(f"Positive Predictive Value: {positive_predictive_value:.4f}")

print(f"Negative Predictive Value: {negative_predictive_value:.4f}")

print(f"Sensitivity (Recall for class {i + 1}): {sensitivity:.4f}")

ytest_one_rf = label_binarize(ytest, classes=np.unique(y))

mlp_AUC = {}

mlp_FPR = {}

mlp_TPR = {}

for i in range(ytest_one_rf.shape[1]):

mlp_FPR[i], mlp_TPR[i], _ = roc_curve(ytest_one_rf[:, i], y_pred_proba[:, i])

mlp_AUC[i] = auc(mlp_FPR[i], mlp_TPR[i])

print("ROC AUC for each class:", mlp_AUC)

mlp_FPR_final = np.unique(np.concatenate([mlp_FPR[i] for i in range(ytest_one_rf.shape[1])]))

mlp_TPR_all = np.zeros_like(mlp_FPR_final)

for i in range(ytest_one_rf.shape[1]):

mlp_TPR_all += np.interp(mlp_FPR_final, mlp_FPR[i], mlp_TPR[i])

mlp_TPR_final = mlp_TPR_all / ytest_one_rf.shape[1]

mlp_AUC_final = auc(mlp_FPR_final, mlp_TPR_final)

print(f"Macro Average AUC with MLP: {mlp_AUC_final}")

plt.figure(figsize=(10, 5), dpi=300)

plt.plot(mlp_FPR[0], mlp_TPR[0], color='b', linestyle='-', label='Class 1 ROC AUC={:.4f}'.format(mlp_AUC[0]), lw=0.8)

plt.plot(mlp_FPR[1], mlp_TPR[1], color='g', linestyle='-', label='Class 2 ROC AUC={:.4f}'.format(mlp_AUC[1]), lw=0.8)

plt.plot(mlp_FPR[2], mlp_TPR[2], color='r', linestyle='-', label='Class 3 ROC AUC={:.4f}'.format(mlp_AUC[2]), lw=0.8)

plt.plot(mlp_FPR[3], mlp_TPR[3], color='c', linestyle='-', label='Class 4 ROC AUC={:.4f}'.format(mlp_AUC[3]), lw=0.8)

plt.plot(mlp_FPR[4], mlp_TPR[4], color='m', linestyle='-', label='Class 5 ROC AUC={:.4f}'.format(mlp_AUC[4]), lw=0.8)

plt.plot(mlp_FPR[5], mlp_TPR[5], color='y', linestyle='-', label='Class 6 ROC AUC={:.4f}'.format(mlp_AUC[5]), lw=0.8)

plt.plot(mlp_FPR[6], mlp_TPR[6], color='#FFA500', linestyle='-', label='Class 7 ROC AUC={:.4f}'.format(mlp_AUC[6]), lw=0.8)

plt.plot(mlp_FPR[7], mlp_TPR[7], color='#9467bd', linestyle='-', label='Class 8 ROC AUC={:.4f}'.format(mlp_AUC[7]), lw=0.8)

plt.plot(mlp_FPR_final, mlp_TPR_final, color='#000000', linestyle='-', label='Macro Average ROC AUC={:.4f}'.format(mlp_AUC_final), lw=1)

plt.plot([0, 1], [0, 1], color='gray', linestyle='--', lw=1, label='45 Degree Reference Line')

plt.tick_params(axis='both', which='major', labelsize=7)

plt.xlabel('False Positive Rate (FPR)', fontsize=7)

plt.ylabel('True Positive Rate (TPR)', fontsize=7)

plt.title('MLP Classification ROC Curves and AUC', fontsize=8)

plt.legend(loc='lower right', framealpha=0.9, fontsize=5)

plt.show()

pr_AUC = {}

pr_Precision = {}

pr_Recall = {}

for i in range(ytest_one_rf.shape[1]):

pr_Recall[i], pr_Precision[i], _ = precision_recall_curve(ytest_one_rf[:, i], y_pred_proba[:, i])

pr_AUC[i] = average_precision_score(ytest_one_rf[:, i], y_pred_proba[:, i])

print("PR AUC for each class:", pr_AUC)

plt.figure(figsize=(10, 5), dpi=300)

plt.plot(pr_Recall[0], pr_Precision[0], color='b', linestyle='-',label=f'Class1 PR AUC={pr_AUC[0]:.4f}', lw=0.8)

plt.plot(pr_Recall[1], pr_Precision[1], color='g', linestyle='-',label=f'Class2 PR AUC={pr_AUC[1]:.4f}', lw=0.8)

plt.plot(pr_Recall[2], pr_Precision[2], color='r', linestyle='-',label=f'Class3 PR AUC={pr_AUC[2]:.4f}', lw=0.8)

plt.plot(pr_Recall[3], pr_Precision[3], color='c', linestyle='-',label=f'Class4 PR AUC={pr_AUC[3]:.4f}', lw=0.8)

plt.plot(pr_Recall[4], pr_Precision[4], color='m', linestyle='-',label=f'Class5 PR AUC={pr_AUC[4]:.4f}', lw=0.8)

plt.plot(pr_Recall[5], pr_Precision[5], color='y', linestyle='-',label=f'Class6 PR AUC={pr_AUC[5]:.4f}', lw=0.8)

plt.plot(pr_Recall[6], pr_Precision[6], color='#FFA500', linestyle='-',label=f'Class7 PR AUC={pr_AUC[6]:.4f}', lw=0.8)

plt.plot(pr_Recall[7], pr_Precision[7], color='#9467bd', linestyle='-',label=f'Class8 PR AUC={pr_AUC[7]:.4f}', lw=0.8)

plt.tick_params(axis='both', which='major', labelsize=7)

plt.xlabel('Recall', fontsize=7)

plt.ylabel('Precision', fontsize=7)

plt.title('MLP Classification PR Curves and AUC', fontsize=8)

plt.legend(loc='lower right', framealpha=0.9, fontsize=5)

plt.show()

DT

import numpy as np

import pandas as pd

import matplotlib.pyplot as plt

from sklearn.model_selection import train_test_split

from sklearn.metrics import classification_report, confusion_matrix, roc_curve, auc, precision_recall_curve, \

average_precision_score, roc_auc_score

from sklearn.preprocessing import MinMaxScaler, label_binarize

import seaborn as sns

from sklearn.tree import DecisionTreeClassifier

plt.rcParams['font.family'] = 'Times New Roman'

plt.rcParams['font.size'] = 10

plt.rcParams['axes.unicode_minus'] = False

data = pd.read_csv('')

X = data.iloc[:, 1:]

y = data.iloc[:, 0]

xtrain, xtest, ytrain, ytest = train_test_split(X, y, test_size=0.3, random_state=42)

scaler = MinMaxScaler()

xtrain_s = scaler.fit_transform(xtrain)

xtest_s = scaler.transform(xtest)

model_rf = DecisionTreeClassifier()

model_rf.fit(xtrain_s, ytrain)

y_pred = model_rf.predict(xtest_s)

y_pred_proba = model_rf.predict_proba(xtest_s)

print("Classification Report:")

print(classification_report(ytest, y_pred))

conf_matrix = confusion_matrix(ytest, y_pred)

plt.figure(figsize=(10, 5), dpi=300)

sns.heatmap(conf_matrix, annot=True, annot_kws={'size':4},

fmt='d', cmap='YlGnBu', cbar_kws={'shrink': 0.75})

plt.tick_params(axis='both', which='major', labelsize=7)

plt.xlabel('Predicted Label', fontsize=7)

plt.ylabel('True Label', fontsize=7)

plt.title('Confusion matrix heat map', fontsize=8)

plt.show()

num_classes = conf_matrix.shape[0]

for i in range(num_classes):

tp = conf_matrix[i, i] # True Positive for class i

fp = conf_matrix[:, i].sum() - tp # False Positive for class i

fn = conf_matrix[i, :].sum() - tp # False Negative for class i

tn = conf_matrix.sum() - (tp + fp + fn) # True Negative for class i

specificity = tn / (tn + fp) if (tn + fp) != 0 else 0

positive_predictive_value = tp / (tp + fp) if (tp + fp) != 0 else 0

negative_predictive_value = tn / (tn + fn) if (tn + fn) != 0 else 0

sensitivity = recall = tp / (tp + fn) if (tp + fn) != 0 else 0

print(f"Class {i + 1}:")

print(f"Specificity: {specificity:.4f}")

print(f"Positive Predictive Value: {positive_predictive_value:.4f}")

print(f"Negative Predictive Value: {negative_predictive_value:.4f}")

print(f"Sensitivity (Recall for class {i + 1}): {sensitivity:.4f}")

ytest_one_rf = label_binarize(ytest, classes=np.unique(y))

rf_AUC = {}

rf_FPR = {}

rf_TPR = {}

for i in range(ytest_one_rf.shape[1]):

rf_FPR[i], rf_TPR[i], _ = roc_curve(ytest_one_rf[:, i], y_pred_proba[:, i])

rf_AUC[i] = auc(rf_FPR[i], rf_TPR[i])

print("ROC AUC for each class:", rf_AUC)

rf_FPR_final = np.unique(np.concatenate([rf_FPR[i] for i in range(ytest_one_rf.shape[1])]))

rf_TPR_all = np.zeros_like(rf_FPR_final)

for i in range(ytest_one_rf.shape[1]):

rf_TPR_all += np.interp(rf_FPR_final, rf_FPR[i], rf_TPR[i])

rf_TPR_final = rf_TPR_all / ytest_one_rf.shape[1]

rf_AUC_final = auc(rf_FPR_final, rf_TPR_final)

print(f"Macro Average AUC with XGBoost: {rf_AUC_final}")

plt.figure(figsize=(10, 5), dpi=300)

plt.plot(rf_FPR[0], rf_TPR[0], color='b', linestyle='-', label='Class 1 ROC AUC={:.4f}'.format(rf_AUC[0]), lw=0.8)

plt.plot(rf_FPR[1], rf_TPR[1], color='g', linestyle='-', label='Class 2 ROC AUC={:.4f}'.format(rf_AUC[1]), lw=0.8)

plt.plot(rf_FPR[2], rf_TPR[2], color='r', linestyle='-', label='Class 3 ROC AUC={:.4f}'.format(rf_AUC[2]), lw=0.8)

plt.plot(rf_FPR[3], rf_TPR[3], color='c', linestyle='-', label='Class 4 ROC AUC={:.4f}'.format(rf_AUC[3]), lw=0.8)

plt.plot(rf_FPR[4], rf_TPR[4], color='m', linestyle='-', label='Class 5 ROC AUC={:.4f}'.format(rf_AUC[4]), lw=0.8)

plt.plot(rf_FPR[5], rf_TPR[5], color='y', linestyle='-', label='Class 6 ROC AUC={:.4f}'.format(rf_AUC[5]), lw=0.8)

plt.plot(rf_FPR[6], rf_TPR[6], color='#FFA500', linestyle='-', label='Class 7 ROC AUC={:.4f}'.format(rf_AUC[6]), lw=0.8)

plt.plot(rf_FPR[7], rf_TPR[7], color='#9467bd', linestyle='-', label='Class 8 ROC AUC={:.4f}'.format(rf_AUC[7]), lw=0.8)

plt.plot(rf_FPR_final, rf_TPR_final, color='#000000', linestyle='-', label='Macro Average ROC AUC={:.4f}'.format(rf_AUC_final), lw=1)

plt.plot([0, 1], [0, 1], color='gray', linestyle='--', lw=1, label='45 Degree Reference Line')

plt.tick_params(axis='both', which='major', labelsize=7)

plt.xlabel('False Positive Rate (FPR)', fontsize=7)

plt.ylabel('True Positive Rate (TPR)', fontsize=7)

plt.title('DecisionTree Classification ROC Curves and AUC', fontsize=8)

plt.legend(loc='lower right', framealpha=0.9, fontsize=5)

plt.show()

pr_AUC = {}

pr_Precision = {}

pr_Recall = {}

for i in range(ytest_one_rf.shape[1]):

pr_Recall[i], pr_Precision[i], _ = precision_recall_curve(ytest_one_rf[:, i], y_pred_proba[:, i])

pr_AUC[i] = average_precision_score(ytest_one_rf[:, i], y_pred_proba[:, i])

print("PR AUC for each class:", pr_AUC)

plt.figure(figsize=(10, 5), dpi=300)

plt.plot(pr_Recall[0], pr_Precision[0], color='b', linestyle='-',label=f'Class1 PR AUC={pr_AUC[0]:.4f}', lw=0.8)

plt.plot(pr_Recall[1], pr_Precision[1], color='g', linestyle='-',label=f'Class2 PR AUC={pr_AUC[1]:.4f}', lw=0.8)

plt.plot(pr_Recall[2], pr_Precision[2], color='r', linestyle='-',label=f'Class3 PR AUC={pr_AUC[2]:.4f}', lw=0.8)

plt.plot(pr_Recall[3], pr_Precision[3], color='c', linestyle='-',label=f'Class4 PR AUC={pr_AUC[3]:.4f}', lw=0.8)

plt.plot(pr_Recall[4], pr_Precision[4], color='m', linestyle='-',label=f'Class5 PR AUC={pr_AUC[4]:.4f}', lw=0.8)

plt.plot(pr_Recall[5], pr_Precision[5], color='y', linestyle='-',label=f'Class6 PR AUC={pr_AUC[5]:.4f}', lw=0.8)

plt.plot(pr_Recall[6], pr_Precision[6], color='k', linestyle='-',label=f'Class7 PR AUC={pr_AUC[6]:.4f}', lw=0.8)

plt.plot(pr_Recall[7], pr_Precision[7], color='#9467bd', linestyle='-',label=f'Class8 PR AUC={pr_AUC[7]:.4f}', lw=0.8)

plt.tick_params(axis='both', which='major', labelsize=7)

plt.xlabel('Recall', fontsize=7)

plt.ylabel('Precision', fontsize=7)

plt.title('DecisionTree Classification PR Curves and AUC', fontsize=8)

plt.legend(loc='lower left', framealpha=0.9, fontsize=5)

plt.show()

**LGB**

import numpy as np

import pandas as pd

import matplotlib.pyplot as plt

from sklearn.model_selection import train_test_split

from sklearn.metrics import classification_report, confusion_matrix, roc_curve, auc, precision_recall_curve, \

average_precision_score, roc_auc_score

from sklearn.preprocessing import MinMaxScaler, label_binarize

import seaborn as sns

import lightgbm as lgb

plt.rcParams['font.family'] = 'Times New Roman'

plt.rcParams['font.size'] = 10

plt.rcParams['axes.unicode_minus'] = False

data = pd.read_csv('')

X = data.iloc[:, 1:]

y = data.iloc[:, 0]

xtrain, xtest, ytrain, ytest = train_test_split(X, y, test_size=0.3, random_state=42)

scaler = MinMaxScaler()

xtrain_s = scaler.fit_transform(xtrain)

xtest_s = scaler.transform(xtest)

model_rf = lgb.LGBMClassifier()

model_rf.fit(xtrain_s, ytrain)

y_pred = model_rf.predict(xtest_s)

y_pred_proba = model_rf.predict_proba(xtest_s)

print("Classification Report:")

print(classification_report(ytest, y_pred))

conf_matrix = confusion_matrix(ytest, y_pred)

plt.figure(figsize=(10, 5), dpi=300)

sns.heatmap(conf_matrix, annot=True, annot_kws={'size':4},

fmt='d', cmap='YlGnBu', cbar_kws={'shrink': 0.75})

plt.tick_params(axis='both', which='major', labelsize=7)

plt.xlabel('Predicted Label', fontsize=7)

plt.ylabel('True Label', fontsize=7)

plt.title('Confusion matrix heat map', fontsize=8)

plt.show()

num_classes = conf_matrix.shape[0]

for i in range(num_classes):

tp = conf_matrix[i, i] # True Positive for class i

fp = conf_matrix[:, i].sum() - tp # False Positive for class i

fn = conf_matrix[i, :].sum() - tp # False Negative for class i

tn = conf_matrix.sum() - (tp + fp + fn) # True Negative for class i

specificity = tn / (tn + fp) if (tn + fp) != 0 else 0

positive_predictive_value = tp / (tp + fp) if (tp + fp) != 0 else 0

negative_predictive_value = tn / (tn + fn) if (tn + fn) != 0 else 0

sensitivity = recall = tp / (tp + fn) if (tp + fn) != 0 else 0

print(f"Class {i + 1}:")

print(f"Specificity: {specificity:.4f}")

print(f"Positive Predictive Value: {positive_predictive_value:.4f}")

print(f"Negative Predictive Value: {negative_predictive_value:.4f}")

print(f"Sensitivity (Recall for class {i + 1}): {sensitivity:.4f}")

ytest_one_rf = label_binarize(ytest, classes=np.unique(y))

rf_AUC = {}

rf_FPR = {}

rf_TPR = {}

for i in range(ytest_one_rf.shape[1]):

rf_FPR[i], rf_TPR[i], _ = roc_curve(ytest_one_rf[:, i], y_pred_proba[:, i])

rf_AUC[i] = auc(rf_FPR[i], rf_TPR[i])

print("ROC AUC for each class:", rf_AUC)

rf_FPR_final = np.unique(np.concatenate([rf_FPR[i] for i in range(ytest_one_rf.shape[1])]))

rf_TPR_all = np.zeros_like(rf_FPR_final)

for i in range(ytest_one_rf.shape[1]):

rf_TPR_all += np.interp(rf_FPR_final, rf_FPR[i], rf_TPR[i])

rf_TPR_final = rf_TPR_all / ytest_one_rf.shape[1]

rf_AUC_final = auc(rf_FPR_final, rf_TPR_final)

print(f"Macro Average AUC with XGBoost: {rf_AUC_final}")

plt.figure(figsize=(10, 5), dpi=300)

plt.plot(rf_FPR[0], rf_TPR[0], color='b', linestyle='-', label='Class 1 ROC AUC={:.4f}'.format(rf_AUC[0]), lw=0.8)

plt.plot(rf_FPR[1], rf_TPR[1], color='g', linestyle='-', label='Class 2 ROC AUC={:.4f}'.format(rf_AUC[1]), lw=0.8)

plt.plot(rf_FPR[2], rf_TPR[2], color='r', linestyle='-', label='Class 3 ROC AUC={:.4f}'.format(rf_AUC[2]), lw=0.8)

plt.plot(rf_FPR[3], rf_TPR[3], color='c', linestyle='-', label='Class 4 ROC AUC={:.4f}'.format(rf_AUC[3]), lw=0.8)

plt.plot(rf_FPR[4], rf_TPR[4], color='m', linestyle='-', label='Class 5 ROC AUC={:.4f}'.format(rf_AUC[4]), lw=0.8)

plt.plot(rf_FPR[5], rf_TPR[5], color='y', linestyle='-', label='Class 6 ROC AUC={:.4f}'.format(rf_AUC[5]), lw=0.8)

plt.plot(rf_FPR[6], rf_TPR[6], color='#FFA500', linestyle='-', label='Class 7 ROC AUC={:.4f}'.format(rf_AUC[6]), lw=0.8)

plt.plot(rf_FPR[7], rf_TPR[7], color='#9467bd', linestyle='-', label='Class 8 ROC AUC={:.4f}'.format(rf_AUC[7]), lw=0.8)

plt.plot(rf_FPR_final, rf_TPR_final, color='#000000', linestyle='-', label='Macro Average ROC AUC={:.4f}'.format(rf_AUC_final), lw=1)

plt.plot([0, 1], [0, 1], color='gray', linestyle='--', lw=1, label='45 Degree Reference Line')

plt.tick_params(axis='both', which='major', labelsize=7)

plt.xlabel('False Positive Rate (FPR)', fontsize=7)

plt.ylabel('True Positive Rate (TPR)', fontsize=7)

plt.title('LGBM Classification ROC Curves and AUC', fontsize=8)

plt.legend(loc='lower right', framealpha=0.9, fontsize=5)

plt.show()

pr_AUC = {}

pr_Precision = {}

pr_Recall = {}

for i in range(ytest_one_rf.shape[1]):

pr_Recall[i], pr_Precision[i], _ = precision_recall_curve(ytest_one_rf[:, i], y_pred_proba[:, i])

pr_AUC[i] = average_precision_score(ytest_one_rf[:, i], y_pred_proba[:, i])

print("PR AUC for each class:", pr_AUC)

plt.figure(figsize=(10, 5), dpi=300)

plt.plot(pr_Recall[0], pr_Precision[0], color='b', linestyle='-',label=f'Class1 PR AUC={pr_AUC[0]:.4f}', lw=0.8)

plt.plot(pr_Recall[1], pr_Precision[1], color='g', linestyle='-',label=f'Class2 PR AUC={pr_AUC[1]:.4f}', lw=0.8)

plt.plot(pr_Recall[2], pr_Precision[2], color='r', linestyle='-',label=f'Class3 PR AUC={pr_AUC[2]:.4f}', lw=0.8)

plt.plot(pr_Recall[3], pr_Precision[3], color='c', linestyle='-',label=f'Class4 PR AUC={pr_AUC[3]:.4f}', lw=0.8)

plt.plot(pr_Recall[4], pr_Precision[4], color='m', linestyle='-',label=f'Class5 PR AUC={pr_AUC[4]:.4f}', lw=0.8)

plt.plot(pr_Recall[5], pr_Precision[5], color='y', linestyle='-',label=f'Class6 PR AUC={pr_AUC[5]:.4f}', lw=0.8)

plt.plot(pr_Recall[6], pr_Precision[6], color='k', linestyle='-',label=f'Class7 PR AUC={pr_AUC[6]:.4f}', lw=0.8)

plt.plot(pr_Recall[7], pr_Precision[7], color='#9467bd', linestyle='-',label=f'Class8 PR AUC={pr_AUC[7]:.4f}', lw=0.8)

plt.tick_params(axis='both', which='major', labelsize=7)

plt.xlabel('Recall', fontsize=7)

plt.ylabel('Precision', fontsize=7)

plt.title('LGBM Classification PR Curves and AUC', fontsize=8)

plt.legend(loc='lower left', framealpha=0.9, fontsize=5)

plt.show()

**KNN**

import numpy as np

import pandas as pd

import matplotlib.pyplot as plt

from sklearn.model_selection import train_test_split

from sklearn.metrics import classification_report, confusion_matrix, roc_curve, auc, precision_recall_curve, \

average_precision_score

from sklearn.neighbors import KNeighborsClassifier

from sklearn.preprocessing import MinMaxScaler, label_binarize

import seaborn as sns

plt.rcParams['font.family'] = 'Times New Roman'

plt.rcParams['font.size'] = 10

plt.rcParams['axes.unicode_minus'] = False

data = pd.read_csv('')

X = data.iloc[:, 1:]

y = data.iloc[:, 0]

xtrain, xtest, ytrain, ytest = train_test_split(X, y, test_size=0.3, random_state=42)

scaler = MinMaxScaler()

xtrain_s = scaler.fit_transform(xtrain)

xtest_s = scaler.transform(xtest)

model_rf = KNeighborsClassifier()

model_rf.fit(xtrain_s, ytrain)

y_pred = model_rf.predict(xtest_s)

y_pred_proba = model_rf.predict_proba(xtest_s)

print("Classification Report:")

print(classification_report(ytest, y_pred))

conf_matrix = confusion_matrix(ytest, y_pred)

plt.figure(figsize=(10, 5), dpi=300)

sns.heatmap(conf_matrix, annot=True, annot_kws={'size':4},

fmt='d', cmap='YlGnBu', cbar_kws={'shrink': 0.75})

plt.tick_params(axis='both', which='major', labelsize=7)

plt.xlabel('Predicted Label', fontsize=7)

plt.ylabel('True Label', fontsize=7)

plt.title('Confusion matrix heat map', fontsize=8)

plt.show()

num_classes = conf_matrix.shape[0]

for i in range(num_classes):

tp = conf_matrix[i, i] # True Positive for class i

fp = conf_matrix[:, i].sum() - tp # False Positive for class i

fn = conf_matrix[i, :].sum() - tp # False Negative for class i

tn = conf_matrix.sum() - (tp + fp + fn) # True Negative for class i

specificity = tn / (tn + fp) if (tn + fp) != 0 else 0

positive_predictive_value = tp / (tp + fp) if (tp + fp) != 0 else 0

negative_predictive_value = tn / (tn + fn) if (tn + fn) != 0 else 0

sensitivity = recall = tp / (tp + fn) if (tp + fn) != 0 else 0

print(f"Class {i + 1}:")

print(f"Specificity: {specificity:.4f}")

print(f"Positive Predictive Value: {positive_predictive_value:.4f}")

print(f"Negative Predictive Value: {negative_predictive_value:.4f}")

print(f"Sensitivity (Recall for class {i + 1}): {sensitivity:.4f}")

ytest_one_rf = label_binarize(ytest, classes=np.unique(y))

rf_AUC = {}

rf_FPR = {}

rf_TPR = {}

for i in range(ytest_one_rf.shape[1]):

rf_FPR[i], rf_TPR[i], _ = roc_curve(ytest_one_rf[:, i], y_pred_proba[:, i])

rf_AUC[i] = auc(rf_FPR[i], rf_TPR[i])

print("ROC AUC for each class:", rf_AUC)

rf_FPR_final = np.unique(np.concatenate([rf_FPR[i] for i in range(ytest_one_rf.shape[1])]))

rf_TPR_all = np.zeros_like(rf_FPR_final)

for i in range(ytest_one_rf.shape[1]):

rf_TPR_all += np.interp(rf_FPR_final, rf_FPR[i], rf_TPR[i])

rf_TPR_final = rf_TPR_all / ytest_one_rf.shape[1]

rf_AUC_final = auc(rf_FPR_final, rf_TPR_final)

print(f"Macro Average AUC with KNN: {rf_AUC_final}")

plt.figure(figsize=(10, 5), dpi=300)

plt.plot(rf_FPR[0], rf_TPR[0], color='b', linestyle='-', label='Class 1 ROC AUC={:.4f}'.format(rf_AUC[0]), lw=0.8)

plt.plot(rf_FPR[1], rf_TPR[1], color='g', linestyle='-', label='Class 2 ROC AUC={:.4f}'.format(rf_AUC[1]), lw=0.8)

plt.plot(rf_FPR[2], rf_TPR[2], color='r', linestyle='-', label='Class 3 ROC AUC={:.4f}'.format(rf_AUC[2]), lw=0.8)

plt.plot(rf_FPR[3], rf_TPR[3], color='c', linestyle='-', label='Class 4 ROC AUC={:.4f}'.format(rf_AUC[3]), lw=0.8)

plt.plot(rf_FPR[4], rf_TPR[4], color='m', linestyle='-', label='Class 5 ROC AUC={:.4f}'.format(rf_AUC[4]), lw=0.8)

plt.plot(rf_FPR[5], rf_TPR[5], color='y', linestyle='-', label='Class 6 ROC AUC={:.4f}'.format(rf_AUC[5]), lw=0.8)

plt.plot(rf_FPR[6], rf_TPR[6], color='#FFA500', linestyle='-', label='Class 7 ROC AUC={:.4f}'.format(rf_AUC[6]), lw=0.8)

plt.plot(rf_FPR[7], rf_TPR[7], color='#9467bd', linestyle='-', label='Class 8 ROC AUC={:.4f}'.format(rf_AUC[7]), lw=0.8)

plt.plot(rf_FPR_final, rf_TPR_final, color='#000000', linestyle='-', label='Macro Average ROC AUC={:.4f}'.format(rf_AUC_final), lw=1)

plt.plot([0, 1], [0, 1], color='gray', linestyle='--', lw=1, label='45 Degree Reference Line')

plt.tick_params(axis='both', which='major', labelsize=7)

plt.xlabel('False Positive Rate (FPR)', fontsize=7)

plt.ylabel('True Positive Rate (TPR)', fontsize=7)

plt.title('KNN Classification ROC Curves and AUC', fontsize=8)

plt.legend(loc='lower right', framealpha=0.9, fontsize=5)

plt.show()

pr_AUC = {}

pr_Precision = {}

pr_Recall = {}

for i in range(ytest_one_rf.shape[1]):

pr_Recall[i], pr_Precision[i], _ = precision_recall_curve(ytest_one_rf[:, i], y_pred_proba[:, i])

pr_AUC[i] = average_precision_score(ytest_one_rf[:, i], y_pred_proba[:, i])

print("PR AUC for each class:", pr_AUC)

plt.figure(figsize=(10, 5), dpi=300)

plt.plot(pr_Recall[0], pr_Precision[0], color='b', linestyle='-',label=f'Class1 PR AUC={pr_AUC[0]:.4f}', lw=0.8)

plt.plot(pr_Recall[1], pr_Precision[1], color='g', linestyle='-',label=f'Class2 PR AUC={pr_AUC[1]:.4f}', lw=0.8)

plt.plot(pr_Recall[2], pr_Precision[2], color='r', linestyle='-',label=f'Class3 PR AUC={pr_AUC[2]:.4f}', lw=0.8)

plt.plot(pr_Recall[3], pr_Precision[3], color='c', linestyle='-',label=f'Class4 PR AUC={pr_AUC[3]:.4f}', lw=0.8)

plt.plot(pr_Recall[4], pr_Precision[4], color='m', linestyle='-',label=f'Class5 PR AUC={pr_AUC[4]:.4f}', lw=0.8)

plt.plot(pr_Recall[5], pr_Precision[5], color='y', linestyle='-',label=f'Class6 PR AUC={pr_AUC[5]:.4f}', lw=0.8)

plt.plot(pr_Recall[6], pr_Precision[6], color='k', linestyle='-',label=f'Class7 PR AUC={pr_AUC[6]:.4f}', lw=0.8)

plt.plot(pr_Recall[7], pr_Precision[7], color='#9467bd', linestyle='-',label=f'Class8 PR AUC={pr_AUC[7]:.4f}', lw=0.8)

plt.tick_params(axis='both', which='major', labelsize=7)

plt.xlabel('Recall', fontsize=7)

plt.ylabel('Precision', fontsize=7)

plt.title('KNN Classification PR Curves and AUC', fontsize=8)

plt.legend(loc='lower left', framealpha=0.9, fontsize=5)

plt.show()

**GB**

import numpy as np

import pandas as pd

import matplotlib.pyplot as plt

from sklearn.ensemble import GradientBoostingClassifier

from sklearn.model_selection import train_test_split

from sklearn.metrics import classification_report, confusion_matrix, roc_curve, auc, precision_recall_curve, \

average_precision_score, roc_auc_score

from sklearn.preprocessing import MinMaxScaler, label_binarize

import seaborn as sns

plt.rcParams['font.family'] = 'Times New Roman'

plt.rcParams['font.size'] = 10

plt.rcParams['axes.unicode_minus'] = False

data = pd.read_csv('')

X = data.iloc[:, 1:]

y = data.iloc[:, 0]

xtrain, xtest, ytrain, ytest = train_test_split(X, y, test_size=0.3, random_state=42)

scaler = MinMaxScaler()

xtrain_s = scaler.fit_transform(xtrain)

xtest_s = scaler.transform(xtest)

model_rf = GradientBoostingClassifier()

model_rf.fit(xtrain_s, ytrain)

y_pred = model_rf.predict(xtest_s)

y_pred_proba = model_rf.predict_proba(xtest_s)

print("Classification Report:")

print(classification_report(ytest, y_pred))

conf_matrix = confusion_matrix(ytest, y_pred)

plt.figure(figsize=(10, 5), dpi=300)

sns.heatmap(conf_matrix, annot=True, annot_kws={'size':4},

fmt='d', cmap='YlGnBu', cbar_kws={'shrink': 0.75})

plt.tick_params(axis='both', which='major', labelsize=7)

plt.xlabel('Predicted Label', fontsize=7)

plt.ylabel('True Label', fontsize=7)

plt.title('Confusion matrix heat map', fontsize=8)

plt.show()

num_classes = conf_matrix.shape[0]

for i in range(num_classes):

tp = conf_matrix[i, i] # True Positive for class i

fp = conf_matrix[:, i].sum() - tp # False Positive for class i

fn = conf_matrix[i, :].sum() - tp # False Negative for class i

tn = conf_matrix.sum() - (tp + fp + fn) # True Negative for class i

specificity = tn / (tn + fp) if (tn + fp) != 0 else 0

positive_predictive_value = tp / (tp + fp) if (tp + fp) != 0 else 0

negative_predictive_value = tn / (tn + fn) if (tn + fn) != 0 else 0

sensitivity = recall = tp / (tp + fn) if (tp + fn) != 0 else 0

print(f"Class {i + 1}:")

print(f"Specificity: {specificity:.4f}")

print(f"Positive Predictive Value: {positive_predictive_value:.4f}")

print(f"Negative Predictive Value: {negative_predictive_value:.4f}")

print(f"Sensitivity (Recall for class {i + 1}): {sensitivity:.4f}")

ytest_one_rf = label_binarize(ytest, classes=np.unique(y))

rf_AUC = {}

rf_FPR = {}

rf_TPR = {}

for i in range(ytest_one_rf.shape[1]):

rf_FPR[i], rf_TPR[i], _ = roc_curve(ytest_one_rf[:, i], y_pred_proba[:, i])

rf_AUC[i] = auc(rf_FPR[i], rf_TPR[i])

print("ROC AUC for each class:", rf_AUC)

rf_FPR_final = np.unique(np.concatenate([rf_FPR[i] for i in range(ytest_one_rf.shape[1])]))

rf_TPR_all = np.zeros_like(rf_FPR_final)

for i in range(ytest_one_rf.shape[1]):

rf_TPR_all += np.interp(rf_FPR_final, rf_FPR[i], rf_TPR[i])

rf_TPR_final = rf_TPR_all / ytest_one_rf.shape[1]

rf_AUC_final = auc(rf_FPR_final, rf_TPR_final)

print(f"Macro Average AUC with GradientBoosting: {rf_AUC_final}")

plt.figure(figsize=(10, 5), dpi=300)

plt.plot(rf_FPR[0], rf_TPR[0], color='b', linestyle='-', label='Class 1 ROC AUC={:.4f}'.format(rf_AUC[0]), lw=0.8)

plt.plot(rf_FPR[1], rf_TPR[1], color='g', linestyle='-', label='Class 2 ROC AUC={:.4f}'.format(rf_AUC[1]), lw=0.8)

plt.plot(rf_FPR[2], rf_TPR[2], color='r', linestyle='-', label='Class 3 ROC AUC={:.4f}'.format(rf_AUC[2]), lw=0.8)

plt.plot(rf_FPR[3], rf_TPR[3], color='c', linestyle='-', label='Class 4 ROC AUC={:.4f}'.format(rf_AUC[3]), lw=0.8)

plt.plot(rf_FPR[4], rf_TPR[4], color='m', linestyle='-', label='Class 5 ROC AUC={:.4f}'.format(rf_AUC[4]), lw=0.8)

plt.plot(rf_FPR[5], rf_TPR[5], color='y', linestyle='-', label='Class 6 ROC AUC={:.4f}'.format(rf_AUC[5]), lw=0.8)

plt.plot(rf_FPR[6], rf_TPR[6], color='#FFA500', linestyle='-', label='Class 7 ROC AUC={:.4f}'.format(rf_AUC[6]), lw=0.8)

plt.plot(rf_FPR[7], rf_TPR[7], color='#9467bd', linestyle='-', label='Class 8 ROC AUC={:.4f}'.format(rf_AUC[7]), lw=0.8)

plt.plot(rf_FPR_final, rf_TPR_final, color='#000000', linestyle='-', label='Macro Average ROC AUC={:.4f}'.format(rf_AUC_final), lw=1)

plt.plot([0, 1], [0, 1], color='gray', linestyle='--', lw=1, label='45 Degree Reference Line')

plt.tick_params(axis='both', which='major', labelsize=7)

plt.xlabel('False Positive Rate (FPR)', fontsize=7)

plt.ylabel('True Positive Rate (TPR)', fontsize=7)

plt.title('GradientBoosting Classification ROC Curves and AUC', fontsize=8)

plt.legend(loc='lower right', framealpha=0.9, fontsize=5)

plt.show()

pr_AUC = {}

pr_Precision = {}

pr_Recall = {}

for i in range(ytest_one_rf.shape[1]):

pr_Recall[i], pr_Precision[i], _ = precision_recall_curve(ytest_one_rf[:, i], y_pred_proba[:, i])

pr_AUC[i] = average_precision_score(ytest_one_rf[:, i], y_pred_proba[:, i])

print("PR AUC for each class:", pr_AUC)

plt.figure(figsize=(10, 5), dpi=300)

plt.plot(pr_Recall[0], pr_Precision[0], color='b', linestyle='-',label=f'Class1 PR AUC={pr_AUC[0]:.4f}', lw=0.8)

plt.plot(pr_Recall[1], pr_Precision[1], color='g', linestyle='-',label=f'Class2 PR AUC={pr_AUC[1]:.4f}', lw=0.8)

plt.plot(pr_Recall[2], pr_Precision[2], color='r', linestyle='-',label=f'Class3 PR AUC={pr_AUC[2]:.4f}', lw=0.8)

plt.plot(pr_Recall[3], pr_Precision[3], color='c', linestyle='-',label=f'Class4 PR AUC={pr_AUC[3]:.4f}', lw=0.8)

plt.plot(pr_Recall[4], pr_Precision[4], color='m', linestyle='-',label=f'Class5 PR AUC={pr_AUC[4]:.4f}', lw=0.8)

plt.plot(pr_Recall[5], pr_Precision[5], color='y', linestyle='-',label=f'Class6 PR AUC={pr_AUC[5]:.4f}', lw=0.8)

plt.plot(pr_Recall[6], pr_Precision[6], color='k', linestyle='-',label=f'Class7 PR AUC={pr_AUC[6]:.4f}', lw=0.8)

plt.plot(pr_Recall[7], pr_Precision[7], color='#9467bd', linestyle='-',label=f'Class8 PR AUC={pr_AUC[7]:.4f}', lw=0.8)

plt.tick_params(axis='both', which='major', labelsize=7)

plt.xlabel('Recall', fontsize=7)

plt.ylabel('Precision', fontsize=7)

plt.title('GradientBoosting Classification PR Curves and AUC', fontsize=8)

plt.legend(loc='lower left', framealpha=0.9, fontsize=5)

plt.show()

**RF**

import numpy as np

import pandas as pd

import matplotlib.pyplot as plt

from sklearn.model_selection import train_test_split

from sklearn.ensemble import RandomForestClassifier

from sklearn.metrics import classification_report, confusion_matrix, roc_curve, auc, precision_recall_curve, \

average_precision_score, roc_auc_score

from sklearn.preprocessing import MinMaxScaler, label_binarize

import seaborn as sns

plt.rcParams['font.family'] = 'Times New Roman'

plt.rcParams['font.size'] = 10

plt.rcParams['axes.unicode_minus'] = False

data = pd.read_csv('')

X = data.iloc[:, 1:]

y = data.iloc[:, 0]

xtrain, xtest, ytrain, ytest = train_test_split(X, y, test_size=0.3, random_state=42)

scaler = MinMaxScaler()

xtrain_s = scaler.fit_transform(xtrain)

xtest_s = scaler.transform(xtest)

model_rf = RandomForestClassifier()

model_rf.fit(xtrain_s, ytrain)

y_pred = model_rf.predict(xtest_s)

y_pred_proba = model_rf.predict_proba(xtest_s)

print("Classification Report:")

print(classification_report(ytest, y_pred))

conf_matrix = confusion_matrix(ytest, y_pred)

plt.figure(figsize=(10, 5), dpi=300)

sns.heatmap(conf_matrix, annot=True, annot_kws={'size':4},

fmt='d', cmap='YlGnBu', cbar_kws={'shrink': 0.75})

plt.tick_params(axis='both', which='major', labelsize=7)

plt.xlabel('Predicted Label', fontsize=7)

plt.ylabel('True Label', fontsize=7)

plt.title('Confusion matrix heat map', fontsize=8)

plt.show()

num_classes = conf_matrix.shape[0]

for i in range(num_classes):

tp = conf_matrix[i, i] # True Positive for class i

fp = conf_matrix[:, i].sum() - tp # False Positive for class i

fn = conf_matrix[i, :].sum() - tp # False Negative for class i

tn = conf_matrix.sum() - (tp + fp + fn) # True Negative for class i

specificity = tn / (tn + fp) if (tn + fp) != 0 else 0

positive_predictive_value = tp / (tp + fp) if (tp + fp) != 0 else 0

negative_predictive_value = tn / (tn + fn) if (tn + fn) != 0 else 0

sensitivity = recall = tp / (tp + fn) if (tp + fn) != 0 else 0

print(f"Class {i + 1}:")

print(f"Specificity: {specificity:.4f}")

print(f"Positive Predictive Value: {positive_predictive_value:.4f}")

print(f"Negative Predictive Value: {negative_predictive_value:.4f}")

print(f"Sensitivity (Recall for class {i + 1}): {sensitivity:.4f}")

ytest_one_rf = label_binarize(ytest, classes=np.unique(y))

rf_AUC = {}

rf_FPR = {}

rf_TPR = {}

for i in range(ytest_one_rf.shape[1]):

rf_FPR[i], rf_TPR[i], _ = roc_curve(ytest_one_rf[:, i], y_pred_proba[:, i])

rf_AUC[i] = auc(rf_FPR[i], rf_TPR[i])

print("ROC AUC for each class:", rf_AUC)

rf_FPR_final = np.unique(np.concatenate([rf_FPR[i] for i in range(ytest_one_rf.shape[1])]))

rf_TPR_all = np.zeros_like(rf_FPR_final)

for i in range(ytest_one_rf.shape[1]):

rf_TPR_all += np.interp(rf_FPR_final, rf_FPR[i], rf_TPR[i])

rf_TPR_final = rf_TPR_all / ytest_one_rf.shape[1]

rf_AUC_final = auc(rf_FPR_final, rf_TPR_final)

print(f"Macro Average AUC with Random Forest: {rf_AUC_final}")

plt.figure(figsize=(10, 5), dpi=300)

plt.plot(rf_FPR[0], rf_TPR[0], color='b', linestyle='-', label='Class 1 ROC AUC={:.4f}'.format(rf_AUC[0]), lw=0.8)

plt.plot(rf_FPR[1], rf_TPR[1], color='g', linestyle='-', label='Class 2 ROC AUC={:.4f}'.format(rf_AUC[1]), lw=0.8)

plt.plot(rf_FPR[2], rf_TPR[2], color='r', linestyle='-', label='Class 3 ROC AUC={:.4f}'.format(rf_AUC[2]), lw=0.8)

plt.plot(rf_FPR[3], rf_TPR[3], color='c', linestyle='-', label='Class 4 ROC AUC={:.4f}'.format(rf_AUC[3]), lw=0.8)

plt.plot(rf_FPR[4], rf_TPR[4], color='m', linestyle='-', label='Class 5 ROC AUC={:.4f}'.format(rf_AUC[4]), lw=0.8)

plt.plot(rf_FPR[5], rf_TPR[5], color='y', linestyle='-', label='Class 6 ROC AUC={:.4f}'.format(rf_AUC[5]), lw=0.8)

plt.plot(rf_FPR[6], rf_TPR[6], color='#FFA500', linestyle='-', label='Class 7 ROC AUC={:.4f}'.format(rf_AUC[6]), lw=0.8)

plt.plot(rf_FPR[7], rf_TPR[7], color='#9467bd', linestyle='-', label='Class 8 ROC AUC={:.4f}'.format(rf_AUC[7]), lw=0.8)

plt.plot(rf_FPR_final, rf_TPR_final, color='#000000', linestyle='-', label='Macro Average ROC AUC={:.4f}'.format(rf_AUC_final), lw=1)

plt.plot([0, 1], [0, 1], color='gray', linestyle='--', lw=1, label='45 Degree Reference Line')

plt.tick_params(axis='both', which='major', labelsize=7)

plt.xlabel('False Positive Rate (FPR)', fontsize=7)

plt.ylabel('True Positive Rate (TPR)', fontsize=7)

plt.title('Random Forest Classification ROC Curves and AUC', fontsize=8)

plt.legend(loc='lower right', framealpha=0.9, fontsize=5)

plt.savefig('RF_optimized.pdf', format='pdf', bbox_inches='tight')

plt.show()

pr_AUC = {}

pr_Precision = {}

pr_Recall = {}

for i in range(ytest_one_rf.shape[1]):

pr_Recall[i], pr_Precision[i], _ = precision_recall_curve(ytest_one_rf[:, i], y_pred_proba[:, i])

pr_AUC[i] = average_precision_score(ytest_one_rf[:, i], y_pred_proba[:, i])

print("PR AUC for each class:", pr_AUC)

plt.figure(figsize=(10, 5), dpi=300)

plt.plot(pr_Recall[0], pr_Precision[0], color='b', linestyle='-',label=f'Class1 PR AUC={pr_AUC[0]:.4f}', lw=0.8)

plt.plot(pr_Recall[1], pr_Precision[1], color='g', linestyle='-',label=f'Class2 PR AUC={pr_AUC[1]:.4f}', lw=0.8)

plt.plot(pr_Recall[2], pr_Precision[2], color='r', linestyle='-',label=f'Class3 PR AUC={pr_AUC[2]:.4f}', lw=0.8)

plt.plot(pr_Recall[3], pr_Precision[3], color='c', linestyle='-',label=f'Class4 PR AUC={pr_AUC[3]:.4f}', lw=0.8)

plt.plot(pr_Recall[4], pr_Precision[4], color='m', linestyle='-',label=f'Class5 PR AUC={pr_AUC[4]:.4f}', lw=0.8)

plt.plot(pr_Recall[5], pr_Precision[5], color='y', linestyle='-',label=f'Class6 PR AUC={pr_AUC[5]:.4f}', lw=0.8)

plt.plot(pr_Recall[6], pr_Precision[6], color='k', linestyle='-',label=f'Class7 PR AUC={pr_AUC[6]:.4f}', lw=0.8)

plt.plot(pr_Recall[7], pr_Precision[7], color='#9467bd', linestyle='-',label=f'Class8 PR AUC={pr_AUC[7]:.4f}', lw=0.8)

plt.tick_params(axis='both', which='major', labelsize=7)

plt.xlabel('Recall', fontsize=7)

plt.ylabel('Precision', fontsize=7)

plt.title('Random Forest Classification PR Curves and AUC', fontsize=8)

plt.legend(loc='lower left', framealpha=0.9, fontsize=5)

plt.savefig('RF_PR_curves.pdf', format='pdf', bbox_inches='tight')

plt.show()

**SVM**

import numpy as np

import pandas as pd

import matplotlib.pyplot as plt

from sklearn.model_selection import train_test_split

from sklearn.metrics import classification_report, confusion_matrix, roc_curve, auc, precision_recall_curve, \

average_precision_score

from sklearn.preprocessing import MinMaxScaler, label_binarize

import seaborn as sns

from sklearn.svm import SVC

plt.rcParams['font.family'] = 'Times New Roman'

plt.rcParams['font.size'] = 10

plt.rcParams['axes.unicode_minus'] = False

data = pd.read_csv('')

X = data.iloc[:, 1:]

y = data.iloc[:, 0]

xtrain, xtest, ytrain, ytest = train_test_split(X, y, test_size=0.3, random_state=42)

scaler = MinMaxScaler()

xtrain_s = scaler.fit_transform(xtrain)

xtest_s = scaler.transform(xtest)

model_rf = SVC()

model_rf.fit(xtrain_s, ytrain)

y_pred = model_rf.predict(xtest_s)

y_pred_proba = model_rf.predict_proba(xtest_s)

print("Classification Report:")

print(classification_report(ytest, y_pred))

conf_matrix = confusion_matrix(ytest, y_pred)

plt.figure(figsize=(10, 5), dpi=300)

sns.heatmap(conf_matrix, annot=True, annot_kws={'size':4},

fmt='d', cmap='YlGnBu', cbar_kws={'shrink': 0.75})

plt.tick_params(axis='both', which='major', labelsize=7)

plt.xlabel('Predicted Label', fontsize=7)

plt.ylabel('True Label', fontsize=7)

plt.title('Confusion matrix heat map', fontsize=8)

plt.show()

num_classes = conf_matrix.shape[0]

for i in range(num_classes):

tp = conf_matrix[i, i] # True Positive for class i

fp = conf_matrix[:, i].sum() - tp # False Positive for class i

fn = conf_matrix[i, :].sum() - tp # False Negative for class i

tn = conf_matrix.sum() - (tp + fp + fn) # True Negative for class i

specificity = tn / (tn + fp) if (tn + fp) != 0 else 0

positive_predictive_value = tp / (tp + fp) if (tp + fp) != 0 else 0

negative_predictive_value = tn / (tn + fn) if (tn + fn) != 0 else 0

sensitivity = recall = tp / (tp + fn) if (tp + fn) != 0 else 0

print(f"Class {i + 1}:")

print(f"Specificity: {specificity:.4f}")

print(f"Positive Predictive Value: {positive_predictive_value:.4f}")

print(f"Negative Predictive Value: {negative_predictive_value:.4f}")

print(f"Sensitivity (Recall for class {i + 1}): {sensitivity:.4f}")

ytest_one_rf = label_binarize(ytest, classes=np.unique(y))

rf_AUC = {}

rf_FPR = {}

rf_TPR = {}

for i in range(ytest_one_rf.shape[1]):

rf_FPR[i], rf_TPR[i], _ = roc_curve(ytest_one_rf[:, i], y_pred_proba[:, i])

rf_AUC[i] = auc(rf_FPR[i], rf_TPR[i])

print("ROC AUC for each class:", rf_AUC)

rf_FPR_final = np.unique(np.concatenate([rf_FPR[i] for i in range(ytest_one_rf.shape[1])]))

rf_TPR_all = np.zeros_like(rf_FPR_final)

for i in range(ytest_one_rf.shape[1]):

rf_TPR_all += np.interp(rf_FPR_final, rf_FPR[i], rf_TPR[i])

rf_TPR_final = rf_TPR_all / ytest_one_rf.shape[1]

rf_AUC_final = auc(rf_FPR_final, rf_TPR_final)

print(f"Macro Average AUC with SVM: {rf_AUC_final}")

plt.figure(figsize=(10, 5), dpi=300)

plt.plot(rf_FPR[0], rf_TPR[0], color='b', linestyle='-', label='Class 1 ROC AUC={:.4f}'.format(rf_AUC[0]), lw=0.8)

plt.plot(rf_FPR[1], rf_TPR[1], color='g', linestyle='-', label='Class 2 ROC AUC={:.4f}'.format(rf_AUC[1]), lw=0.8)

plt.plot(rf_FPR[2], rf_TPR[2], color='r', linestyle='-', label='Class 3 ROC AUC={:.4f}'.format(rf_AUC[2]), lw=0.8)

plt.plot(rf_FPR[3], rf_TPR[3], color='c', linestyle='-', label='Class 4 ROC AUC={:.4f}'.format(rf_AUC[3]), lw=0.8)

plt.plot(rf_FPR[4], rf_TPR[4], color='m', linestyle='-', label='Class 5 ROC AUC={:.4f}'.format(rf_AUC[4]), lw=0.8)

plt.plot(rf_FPR[5], rf_TPR[5], color='y', linestyle='-', label='Class 6 ROC AUC={:.4f}'.format(rf_AUC[5]), lw=0.8)

plt.plot(rf_FPR[6], rf_TPR[6], color='#FFA500', linestyle='-', label='Class 7 ROC AUC={:.4f}'.format(rf_AUC[6]), lw=0.8)

plt.plot(rf_FPR[7], rf_TPR[7], color='#9467bd', linestyle='-', label='Class 8 ROC AUC={:.4f}'.format(rf_AUC[7]), lw=0.8)

plt.plot(rf_FPR_final, rf_TPR_final, color='#000000', linestyle='-', label='Macro Average ROC AUC={:.4f}'.format(rf_AUC_final), lw=1)

plt.plot([0, 1], [0, 1], color='gray', linestyle='--', lw=1, label='45 Degree Reference Line')

plt.tick_params(axis='both', which='major', labelsize=7)

plt.xlabel('False Positive Rate (FPR)', fontsize=7)

plt.ylabel('True Positive Rate (TPR)', fontsize=7)

plt.title('SVM Classification ROC Curves and AUC', fontsize=8)

plt.legend(loc='lower right', framealpha=0.9, fontsize=5)

plt.show()

pr_AUC = {}

pr_Precision = {}

pr_Recall = {}

for i in range(ytest_one_rf.shape[1]):

pr_Recall[i], pr_Precision[i], _ = precision_recall_curve(ytest_one_rf[:, i], y_pred_proba[:, i])

pr_AUC[i] = average_precision_score(ytest_one_rf[:, i], y_pred_proba[:, i])

print("PR AUC for each class:", pr_AUC)

plt.figure(figsize=(10, 5), dpi=300)

plt.plot(pr_Recall[0], pr_Precision[0], color='b', linestyle='-',label=f'Class1 PR AUC={pr_AUC[0]:.4f}', lw=0.8)

plt.plot(pr_Recall[1], pr_Precision[1], color='g', linestyle='-',label=f'Class2 PR AUC={pr_AUC[1]:.4f}', lw=0.8)

plt.plot(pr_Recall[2], pr_Precision[2], color='r', linestyle='-',label=f'Class3 PR AUC={pr_AUC[2]:.4f}', lw=0.8)

plt.plot(pr_Recall[3], pr_Precision[3], color='c', linestyle='-',label=f'Class4 PR AUC={pr_AUC[3]:.4f}', lw=0.8)

plt.plot(pr_Recall[4], pr_Precision[4], color='m', linestyle='-',label=f'Class5 PR AUC={pr_AUC[4]:.4f}', lw=0.8)

plt.plot(pr_Recall[5], pr_Precision[5], color='y', linestyle='-',label=f'Class6 PR AUC={pr_AUC[5]:.4f}', lw=0.8)

plt.plot(pr_Recall[6], pr_Precision[6], color='k', linestyle='-',label=f'Class7 PR AUC={pr_AUC[6]:.4f}', lw=0.8)

plt.plot(pr_Recall[7], pr_Precision[7], color='#9467bd', linestyle='-',label=f'Class8 PR AUC={pr_AUC[7]:.4f}', lw=0.8)

plt.tick_params(axis='both', which='major', labelsize=7)

plt.xlabel('Recall', fontsize=7)

plt.ylabel('Precision', fontsize=7)

plt.title('SVM Classification PR Curves and AUC', fontsize=8)

plt.legend(loc='lower left', framealpha=0.9, fontsize=5)

plt.show()

**XGB**

import numpy as np

import pandas as pd

import matplotlib.pyplot as plt

from sklearn.model_selection import train_test_split

from sklearn.metrics import classification_report, confusion_matrix, roc_curve, auc, precision_recall_curve, \

average_precision_score

from sklearn.preprocessing import MinMaxScaler, label_binarize

import seaborn as sns

import xgboost as xgb

plt.rcParams['font.family'] = 'Times New Roman'

plt.rcParams['font.size'] = 10

plt.rcParams['axes.unicode_minus'] = False

data = pd.read_csv('E:/RS/sm/ALL-Feature/XGBoost/final/XGBoost-16.csv')

X = data.iloc[:, 1:]

y = data.iloc[:, 0]

xtrain, xtest, ytrain, ytest = train_test_split(X, y, test_size=0.3, random_state=42)

scaler = MinMaxScaler()

xtrain_s = scaler.fit_transform(xtrain)

xtest_s = scaler.transform(xtest)

model_rf = xgb.XGBClassifier(colsample_bytree=0.5,learning_rate=0.2,max_depth=3,n_estimators=100)

model_rf.fit(xtrain_s, ytrain)

y_pred = model_rf.predict(xtest_s)

y_pred_proba = model_rf.predict_proba(xtest_s)

print("Classification Report:")

print(classification_report(ytest, y_pred))

conf_matrix = confusion_matrix(ytest, y_pred)

plt.figure(figsize=(10, 5), dpi=300)

sns.heatmap(conf_matrix, annot=True, annot_kws={'size':8},

fmt='d', cmap='YlGnBu', cbar_kws={'shrink': 0.75})

plt.tick_params(axis='both', which='major', labelsize=7)

plt.xlabel('Predicted Label', fontsize=7)

plt.ylabel('True Label', fontsize=7)

plt.title('Confusion matrix heat map', fontsize=8)

plt.show()

num_classes = conf_matrix.shape[0]

for i in range(num_classes):

tp = conf_matrix[i, i] # True Positive for class i

fp = conf_matrix[:, i].sum() - tp # False Positive for class i

fn = conf_matrix[i, :].sum() - tp # False Negative for class i

tn = conf_matrix.sum() - (tp + fp + fn) # True Negative for class i

specificity = tn / (tn + fp) if (tn + fp) != 0 else 0

positive_predictive_value = tp / (tp + fp) if (tp + fp) != 0 else 0

negative_predictive_value = tn / (tn + fn) if (tn + fn) != 0 else 0

sensitivity = recall = tp / (tp + fn) if (tp + fn) != 0 else 0

print(f"Class {i + 1}:")

print(f"Specificity: {specificity:.4f}")

print(f"Positive Predictive Value: {positive_predictive_value:.4f}")

print(f"Negative Predictive Value: {negative_predictive_value:.4f}")

print(f"Sensitivity (Recall for class {i + 1}): {sensitivity:.4f}")

ytest_one_rf = label_binarize(ytest, classes=np.unique(y))

rf_AUC = {}

rf_FPR = {}

rf_TPR = {}

for i in range(ytest_one_rf.shape[1]):

rf_FPR[i], rf_TPR[i], _ = roc_curve(ytest_one_rf[:, i], y_pred_proba[:, i])

rf_AUC[i] = auc(rf_FPR[i], rf_TPR[i])

print("ROC AUC for each class:", rf_AUC)

rf_FPR_final = np.unique(np.concatenate([rf_FPR[i] for i in range(ytest_one_rf.shape[1])]))

rf_TPR_all = np.zeros_like(rf_FPR_final)

for i in range(ytest_one_rf.shape[1]):

rf_TPR_all += np.interp(rf_FPR_final, rf_FPR[i], rf_TPR[i])

rf_TPR_final = rf_TPR_all / ytest_one_rf.shape[1]

rf_AUC_final = auc(rf_FPR_final, rf_TPR_final)

print(f"Macro Average AUC with XGBoost: {rf_AUC_final}")

plt.figure(figsize=(10, 5), dpi=300)

plt.plot(rf_FPR[0], rf_TPR[0], color='b', linestyle='-', label='Class 1 ROC AUC={:.4f}'.format(rf_AUC[0]), lw=0.8)

plt.plot(rf_FPR[1], rf_TPR[1], color='g', linestyle='-', label='Class 2 ROC AUC={:.4f}'.format(rf_AUC[1]), lw=0.8)

plt.plot(rf_FPR[2], rf_TPR[2], color='r', linestyle='-', label='Class 3 ROC AUC={:.4f}'.format(rf_AUC[2]), lw=0.8)

plt.plot(rf_FPR[3], rf_TPR[3], color='c', linestyle='-', label='Class 4 ROC AUC={:.4f}'.format(rf_AUC[3]), lw=0.8)

plt.plot(rf_FPR[4], rf_TPR[4], color='m', linestyle='-', label='Class 5 ROC AUC={:.4f}'.format(rf_AUC[4]), lw=0.8)

plt.plot(rf_FPR[5], rf_TPR[5], color='y', linestyle='-', label='Class 6 ROC AUC={:.4f}'.format(rf_AUC[5]), lw=0.8)

plt.plot(rf_FPR[6], rf_TPR[6], color='#FFA500', linestyle='-', label='Class 7 ROC AUC={:.4f}'.format(rf_AUC[6]), lw=0.8)

plt.plot(rf_FPR[7], rf_TPR[7], color='#9467bd', linestyle='-', label='Class 8 ROC AUC={:.4f}'.format(rf_AUC[7]), lw=0.8)

plt.plot(rf_FPR_final, rf_TPR_final, color='#000000', linestyle='-', label='Macro Average ROC AUC={:.4f}'.format(rf_AUC_final), lw=1)

plt.plot([0, 1], [0, 1], color='gray', linestyle='--', lw=1, label='45 Degree Reference Line')

plt.tick_params(axis='both', which='major', labelsize=7)

plt.xlabel('False Positive Rate (FPR)', fontsize=7)

plt.ylabel('True Positive Rate (TPR)', fontsize=7)

plt.title('XGBoost Classification ROC Curves and AUC', fontsize=8)

plt.legend(loc='lower right', framealpha=0.9, fontsize=5)

plt.show()

pr_AUC = {}

pr_Precision = {}

pr_Recall = {}

for i in range(ytest_one_rf.shape[1]):

pr_Recall[i], pr_Precision[i], _ = precision_recall_curve(ytest_one_rf[:, i], y_pred_proba[:, i])

pr_AUC[i] = average_precision_score(ytest_one_rf[:, i], y_pred_proba[:, i])

print("PR AUC for each class:", pr_AUC)

plt.figure(figsize=(10, 5), dpi=300)

plt.plot(pr_Recall[0], pr_Precision[0], color='b', linestyle='-',label=f'Class1 PR AUC={pr_AUC[0]:.4f}', lw=0.8)

plt.plot(pr_Recall[1], pr_Precision[1], color='g', linestyle='-',label=f'Class2 PR AUC={pr_AUC[1]:.4f}', lw=0.8)

plt.plot(pr_Recall[2], pr_Precision[2], color='r', linestyle='-',label=f'Class3 PR AUC={pr_AUC[2]:.4f}', lw=0.8)

plt.plot(pr_Recall[3], pr_Precision[3], color='c', linestyle='-',label=f'Class4 PR AUC={pr_AUC[3]:.4f}', lw=0.8)

plt.plot(pr_Recall[4], pr_Precision[4], color='m', linestyle='-',label=f'Class5 PR AUC={pr_AUC[4]:.4f}', lw=0.8)

plt.plot(pr_Recall[5], pr_Precision[5], color='y', linestyle='-',label=f'Class6 PR AUC={pr_AUC[5]:.4f}', lw=0.8)

plt.plot(pr_Recall[6], pr_Precision[6], color='k', linestyle='-',label=f'Class7 PR AUC={pr_AUC[6]:.4f}', lw=0.8)

plt.plot(pr_Recall[7], pr_Precision[7], color='#9467bd', linestyle='-',label=f'Class8 PR AUC={pr_AUC[7]:.4f}', lw=0.8)

plt.tick_params(axis='both', which='major', labelsize=7)

plt.xlabel('Recall', fontsize=7)

plt.ylabel('Precision', fontsize=7)

plt.title('XGBoost Classification PR Curves and AUC', fontsize=8)

plt.legend(loc='lower left', framealpha=0.9, fontsize=5)

plt.show()

**GNB**

import numpy as np

import pandas as pd

import matplotlib.pyplot as plt

from sklearn.model_selection import train_test_split

from sklearn.metrics import classification_report, confusion_matrix, roc_curve, auc, precision_recall_curve, \

average_precision_score

from sklearn.naive_bayes import GaussianNB

from sklearn.preprocessing import MinMaxScaler, label_binarize

import seaborn as sns

plt.rcParams['font.family'] = 'Times New Roman'

plt.rcParams['font.size'] = 10

plt.rcParams['axes.unicode_minus'] = False

data = pd.read_csv('')

X = data.iloc[:, 1:]

y = data.iloc[:, 0]

xtrain, xtest, ytrain, ytest = train_test_split(X, y, test_size=0.3, random_state=42)

scaler = MinMaxScaler()

xtrain_s = scaler.fit_transform(xtrain)

xtest_s = scaler.transform(xtest)

model_rf = GaussianNB(var_smoothing = 1e-07)

model_rf.fit(xtrain_s, ytrain)

y_pred = model_rf.predict(xtest_s)

y_pred_proba = model_rf.predict_proba(xtest_s)

print("Classification Report:")

print(classification_report(ytest, y_pred))

conf_matrix = confusion_matrix(ytest, y_pred)

plt.figure(figsize=(10, 5), dpi=300)

sns.heatmap(conf_matrix, annot=True, annot_kws={'size':4},

fmt='d', cmap='YlGnBu', cbar_kws={'shrink': 0.75})

plt.tick_params(axis='both', which='major', labelsize=7)

plt.xlabel('Predicted Label', fontsize=7)

plt.ylabel('True Label', fontsize=7)

plt.title('Confusion matrix heat map', fontsize=8)

plt.show()

num_classes = conf_matrix.shape[0]

for i in range(num_classes):

tp = conf_matrix[i, i] # True Positive for class i

fp = conf_matrix[:, i].sum() - tp # False Positive for class i

fn = conf_matrix[i, :].sum() - tp # False Negative for class i

tn = conf_matrix.sum() - (tp + fp + fn) # True Negative for class i

specificity = tn / (tn + fp) if (tn + fp) != 0 else 0

positive_predictive_value = tp / (tp + fp) if (tp + fp) != 0 else 0

negative_predictive_value = tn / (tn + fn) if (tn + fn) != 0 else 0

sensitivity = recall = tp / (tp + fn) if (tp + fn) != 0 else 0

print(f"Class {i + 1}:")

print(f"Specificity: {specificity:.4f}")

print(f"Positive Predictive Value: {positive_predictive_value:.4f}")

print(f"Negative Predictive Value: {negative_predictive_value:.4f}")

print(f"Sensitivity (Recall for class {i + 1}): {sensitivity:.4f}")

ytest_one_rf = label_binarize(ytest, classes=np.unique(y))

rf_AUC = {}

rf_FPR = {}

rf_TPR = {}

for i in range(ytest_one_rf.shape[1]):

rf_FPR[i], rf_TPR[i], _ = roc_curve(ytest_one_rf[:, i], y_pred_proba[:, i])

rf_AUC[i] = auc(rf_FPR[i], rf_TPR[i])

print("ROC AUC for each class:", rf_AUC)

rf_FPR_final = np.unique(np.concatenate([rf_FPR[i] for i in range(ytest_one_rf.shape[1])]))

rf_TPR_all = np.zeros_like(rf_FPR_final)

for i in range(ytest_one_rf.shape[1]):

rf_TPR_all += np.interp(rf_FPR_final, rf_FPR[i], rf_TPR[i])

rf_TPR_final = rf_TPR_all / ytest_one_rf.shape[1]

rf_AUC_final = auc(rf_FPR_final, rf_TPR_final)

print(f"Macro Average AUC with GaussianNB: {rf_AUC_final}")

plt.figure(figsize=(10, 5), dpi=300)

plt.plot(rf_FPR[0], rf_TPR[0], color='b', linestyle='-', label='Class 1 ROC AUC={:.4f}'.format(rf_AUC[0]), lw=0.8)

plt.plot(rf_FPR[1], rf_TPR[1], color='g', linestyle='-', label='Class 2 ROC AUC={:.4f}'.format(rf_AUC[1]), lw=0.8)

plt.plot(rf_FPR[2], rf_TPR[2], color='r', linestyle='-', label='Class 3 ROC AUC={:.4f}'.format(rf_AUC[2]), lw=0.8)

plt.plot(rf_FPR[3], rf_TPR[3], color='c', linestyle='-', label='Class 4 ROC AUC={:.4f}'.format(rf_AUC[3]), lw=0.8)

plt.plot(rf_FPR[4], rf_TPR[4], color='m', linestyle='-', label='Class 5 ROC AUC={:.4f}'.format(rf_AUC[4]), lw=0.8)

plt.plot(rf_FPR[5], rf_TPR[5], color='y', linestyle='-', label='Class 6 ROC AUC={:.4f}'.format(rf_AUC[5]), lw=0.8)

plt.plot(rf_FPR[6], rf_TPR[6], color='#FFA500', linestyle='-', label='Class 7 ROC AUC={:.4f}'.format(rf_AUC[6]), lw=0.8)

plt.plot(rf_FPR[7], rf_TPR[7], color='#9467bd', linestyle='-', label='Class 8 ROC AUC={:.4f}'.format(rf_AUC[7]), lw=0.8)

plt.plot(rf_FPR_final, rf_TPR_final, color='#000000', linestyle='-', label='Macro Average ROC AUC={:.4f}'.format(rf_AUC_final), lw=1)

plt.plot([0, 1], [0, 1], color='gray', linestyle='--', lw=1, label='45 Degree Reference Line')

plt.tick_params(axis='both', which='major', labelsize=7)

plt.xlabel('False Positive Rate (FPR)', fontsize=7)

plt.ylabel('True Positive Rate (TPR)', fontsize=7)

plt.title('GaussianNB Classification ROC Curves and AUC', fontsize=8)

plt.legend(loc='lower right', framealpha=0.9, fontsize=5)

plt.show()

pr_AUC = {}

pr_Precision = {}

pr_Recall = {}

for i in range(ytest_one_rf.shape[1]):

pr_Recall[i], pr_Precision[i], _ = precision_recall_curve(ytest_one_rf[:, i], y_pred_proba[:, i])

pr_AUC[i] = average_precision_score(ytest_one_rf[:, i], y_pred_proba[:, i])

print("PR AUC for each class:", pr_AUC)

plt.figure(figsize=(10, 5), dpi=300)

plt.plot(pr_Recall[0], pr_Precision[0], color='b', linestyle='-',label=f'Class1 PR AUC={pr_AUC[0]:.4f}', lw=0.8)

plt.plot(pr_Recall[1], pr_Precision[1], color='g', linestyle='-',label=f'Class2 PR AUC={pr_AUC[1]:.4f}', lw=0.8)

plt.plot(pr_Recall[2], pr_Precision[2], color='r', linestyle='-',label=f'Class3 PR AUC={pr_AUC[2]:.4f}', lw=0.8)

plt.plot(pr_Recall[3], pr_Precision[3], color='c', linestyle='-',label=f'Class4 PR AUC={pr_AUC[3]:.4f}', lw=0.8)

plt.plot(pr_Recall[4], pr_Precision[4], color='m', linestyle='-',label=f'Class5 PR AUC={pr_AUC[4]:.4f}', lw=0.8)

plt.plot(pr_Recall[5], pr_Precision[5], color='y', linestyle='-',label=f'Class6 PR AUC={pr_AUC[5]:.4f}', lw=0.8)

plt.plot(pr_Recall[6], pr_Precision[6], color='k', linestyle='-',label=f'Class7 PR AUC={pr_AUC[6]:.4f}', lw=0.8)

plt.plot(pr_Recall[7], pr_Precision[7], color='#9467bd', linestyle='-',label=f'Class8 PR AUC={pr_AUC[7]:.4f}', lw=0.8)

plt.tick_params(axis='both', which='major', labelsize=7)

plt.xlabel('Recall', fontsize=7)

plt.ylabel('Precision', fontsize=7)

plt.title('GaussianNB Classification PR Curves and AUC', fontsize=8)

plt.legend(loc='lower left', framealpha=0.9, fontsize=5)

plt.show()

(4) Model interpretability

Import shap

explainer = shap.Explainer(model.predict_proba, X_train)

shap_values = explainer(X_test)

# shap.summary_plot(shap_values, X_test, feature_names=iris.feature_names)

labels = []

list_of_2d_arrays = [shap_values.values[:, :, i] for i in range()]

shap.summary_plot(list_of_2d_arrays, X_test, class_names=labels, max_display=20)
